# Digital twin predicting diet response before and after long-term fasting

**DOI:** 10.1371/journal.pcbi.1010469

**Published:** 2022-09-12

**Authors:** Oscar Silfvergren, Christian Simonsson, Mattias Ekstedt, Peter Lundberg, Peter Gennemark, Gunnar Cedersund

**Affiliations:** 1 Department of Biomedical Engineering, IMT, Linköping University, Linköping, Sweden; 2 Center for Medical Image Science and Visualisation, Linköping University, Linköping, Sweden; 3 Department of Health, Medicine, and Caring Sciences, Linköping University, Linköping, Sweden; 4 Department of Medical Radiation Physics, and Department of Health, Medicine and Caring Sciences, Linköping University, Linköping, Sweden; 5 Drug Metabolism and Pharmacokinetics, Research and Early Development, Cardiovascular, Renal and Metabolism (CVRM), BioPharmaceuticals R&D, AstraZeneca, Gothenburg, Sweden; Ecole Polytechnique Fédérale de Lausanne, SWITZERLAND

## Abstract

Today, there is great interest in diets proposing new combinations of macronutrient compositions and fasting schedules. Unfortunately, there is little consensus regarding the impact of these different diets, since available studies measure different sets of variables in different populations, thus only providing partial, non-connected insights. We lack an approach for integrating all such partial insights into a useful and interconnected big picture. Herein, we present such an integrating tool. The tool uses a novel mathematical model that describes mechanisms regulating diet response and fasting metabolic fluxes, both for organ-organ crosstalk, and inside the liver. The tool can mechanistically explain and integrate data from several clinical studies, and correctly predict new independent data, including data from a new study. Using this model, we can predict non-measured variables, *e*.*g*. hepatic glycogen and gluconeogenesis, in response to fasting and different diets. Furthermore, we exemplify how such metabolic responses can be successfully adapted to a specific individual’s sex, weight, height, as well as to the individual’s historical data on metabolite dynamics. This tool enables an offline digital twin technology.

## Author summary

Fasting and diet are central components of prevention against cardiovascular disease. Unfortunately, there is little consensus regarding which diet schemes are optimal. This is partially because different clinical studies contribute with different non-connected pieces of knowledge, which have not been fully integrated into a useful and interconnected big picture. In principle, mathematical models describing meal responses could be used for such an integration. However, today’s models still lack critical mechanisms, such as protein metabolism and a dynamic glycogen regulation. Herein, we present a) a new expanded model structure including these mechanisms; b) a set of parameters which can simultaneously describe a wide array of complementary estimation data, in both healthy and diabetic populations; c) a personalisation-script, which allows these generic parameters to be tuned to an individual/sub-population, using demographics (age, weight, height, diabetes status) and historic metabolic data. We exemplify how this personalisation can be used to predict new independent data, including a new clinical study, where a qualitatively new prediction is validated: that an oral protein tolerance test gives a clear response in plasma glucose, after, but not before, a 48h fasting period. Our combined model, parameters, and fitting script lay the foundation for an offline digital twin.

## Introduction

Metabolic dysregulation and obesity lead to many of our most serious diseases: type 2 diabetes, non-alcoholic steatohepatitis (NASH), and cardiovascular diseases. Prevention of these diseases involves proper diet and exercise. Proper dieting involves two central steps: i) figuring out which diet scheme is the best for each person, and ii) making people follow these schemes. Unfortunately, we have still not succeeded with either of these two steps. There is no consensus regarding which of the many proposed diet and fasting schemes is optimal for each person: some restrict fat while allowing for high carbohydrates (HCLF); some do the opposite, i.e. restrict carbohydrates and allow fat (LCHF); some advocate fasting two days a week (5:2); some 16 hours each day (intermediate fasting, IF); and some suggest small, frequent meals (SFM) [1–8]. The reason for this lack of consensus from long-term studies may partially be due to a variety of confounding factors that are hard to monitor in large-scale long-term studies. Well-designed short-term studies show clearer effects. However, in these short-term studies, different studies measure different sets of variables, in different conditions, and in different populations. Such disparate datasets cannot naturally be integrated using traditional methods for data analysis. Finally, there are also many problems regarding compliance to these diets: patients may not believe that the prescribed diet works for them, and/or have problems with motivation and long-term adherence [9–11].

**Fig 1:**
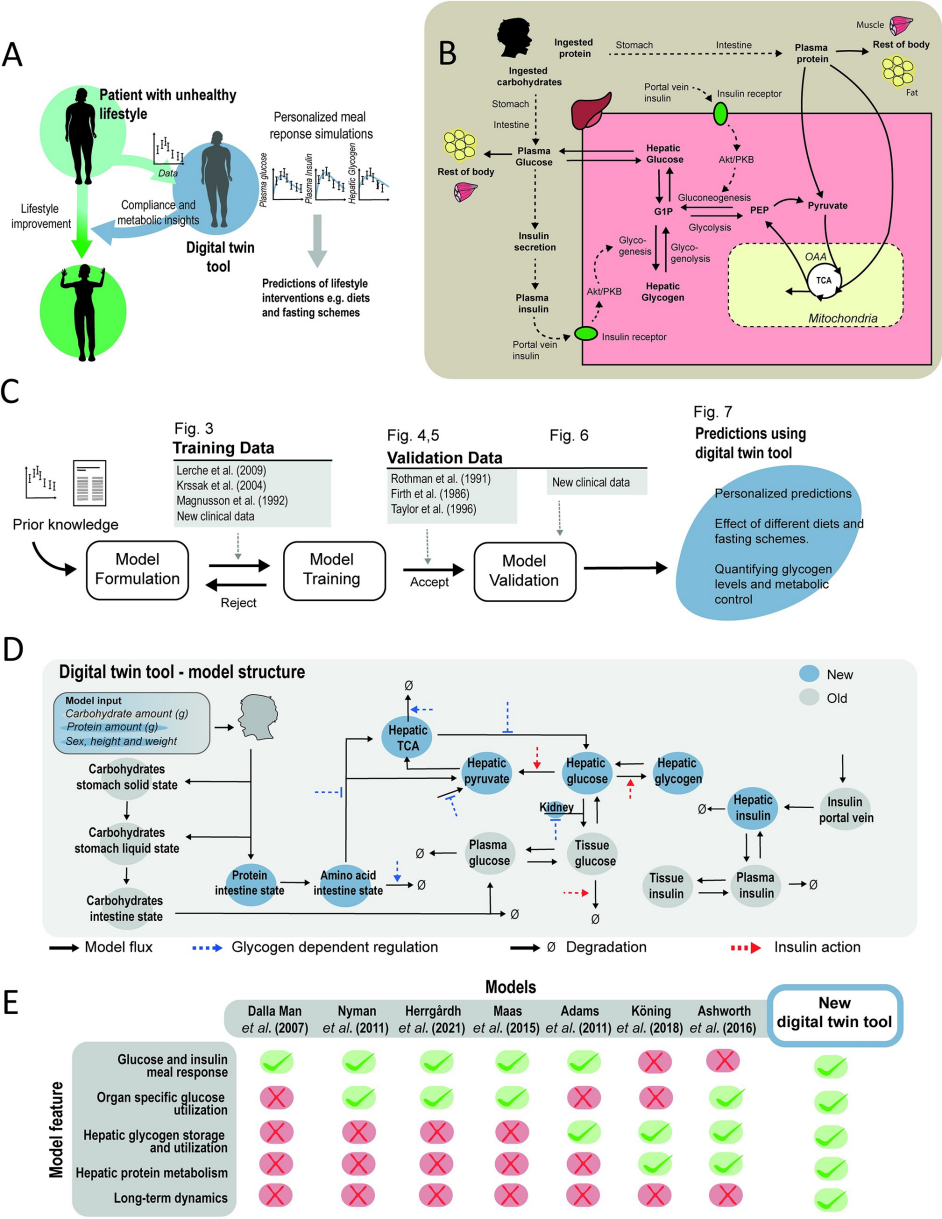
Overview of digital twin tool. (A) Illustration of the idea of a digital twin: use personalised predictions and visualisations to increase the metabolic insights and compliance of a patient to prescribed life-style changes. (B) The physiological and biochemical processes considered herein. (C) Workflow and steps taken in this work. (D) Overview of all reactions and regulations included in the model. (E) Comparison between new digital twin tool and previous physiologically based meal simulation models.

Lately, a new technology has been advocated that potentially can solve both these problems: *digital twins* (Fig 1A) [12–14]. Digital twins come in different categories, e.g. *online twins*, which are updated in real time by connecting with a sensor, and *offline twins*, which are only occasionally updated using new data [15]. Another distinction is between black box twins, utilising machine learning and statistical models, and physiologically based twins. A physiologically based digital twin is a personalised computer model that describes the underlying physiology in a specific person or patient (Fig 1B). An important potential with physiologically based twins is that you can give them a meal or a fasting schedule, and see how they respond, both acutely to each meal, and etiologically over longer periods of time. The physiological nature of such twins implies that they can deal with the first problem mentioned above: lack of consensus. Specific aspects of each study can be incorporated into the appropriate parts of the digital twin (Fig 1C) [16]. For instance, a study that measures the interplay between glucose, insulin, and glycogen, will inform and update those aspects of the twin. Another study that measures other variables will update other aspects of the same twin technology. Finally, a digital twin technology can potentially also help with the second problem: lack of compliance. It is likely that seeing your own digital twin (Fig 1D) improve, maintain, or lose its metabolic function by following different diets, will increase the motivation to follow the right diet [12].

**Fig 2:**
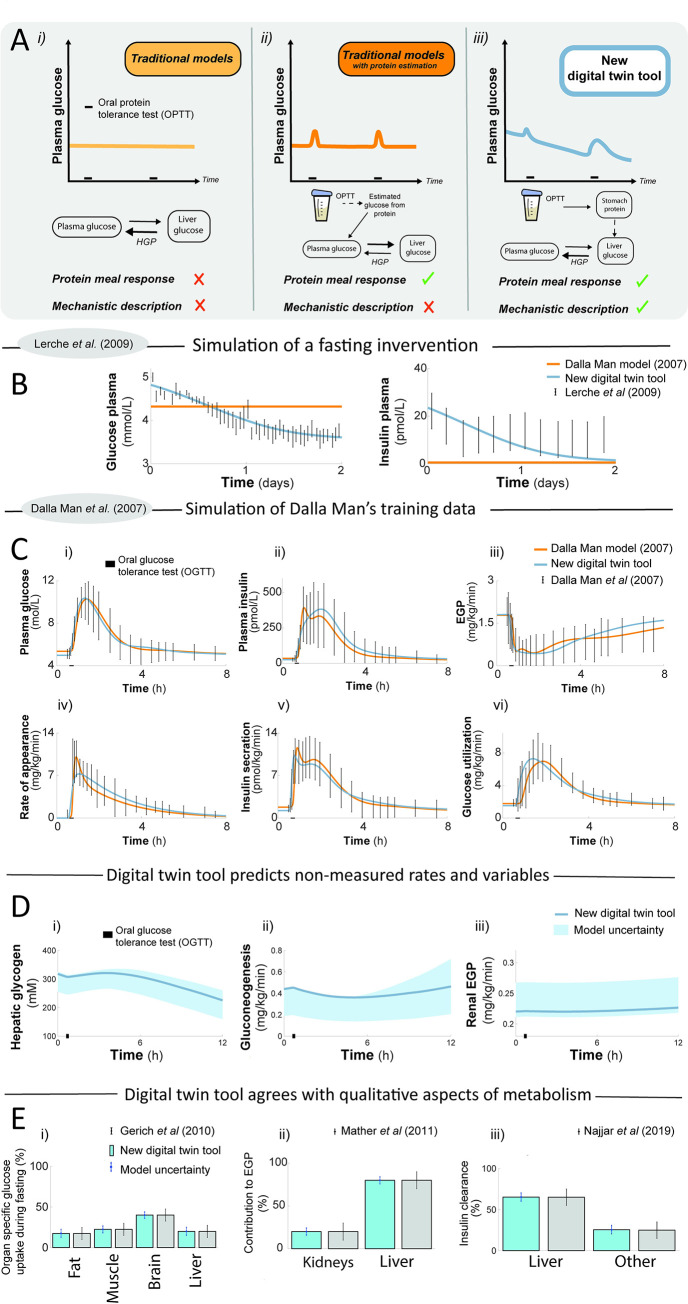
Model features compared to previous model. (A) Overview of the qualitative predictions and improvements in new model, compared to previous models. Previous models can only either i) ignore protein ingestion in glucose simulations, or ii) have a static formula for a phenomenological conversion between protein and plasma glucose, which incorrectly increases the hepatic glucose uptake. In contrast, iii) in the new model, we can have a protein-induced increase in gluconeogenesis and hepatic glucose production, which is much larger after fasting than in a fed state. The arrows and their sizes represent the qualitative relationships between the fluxes between plasma glucose and plasma liver during the second OPTT response, for the three model alternatives. (B) Experimental data (error bars) validating another key difference between the new (blue line) and old model simulations (orange line): the decrease of plasma glucose during fasting. (C) New model (blue line) can describe all of the mechanistic flux data (error bars) that led to the ‘Dalla Man model’ [21], equally as well as that model and its subsequent improvements (orange line). The data shows responses to a mixed meal at 0.5 h (black bar). (D) Examples of predictions of key mechanistic variables that new model can produce, which the original Dalla Man model and its subsequent improvements (including [23]) cannot produce. (E) Qualitative aspects of metabolism compared to simulations, where predictions (blue filled bars) are compared to data (grey filled bars) for organ-specific glucose uptake (i, [32]), organ-specific endogenous glucose production (EGP) (ii,[35]), and insulin clearance (iii, [33]). Model uncertainty is represented as error bars on blue filled bars, and error bars on grey filled bars are qualitative reasonable intervals of each respective metabolic flow or reaction.

**Fig 3:**
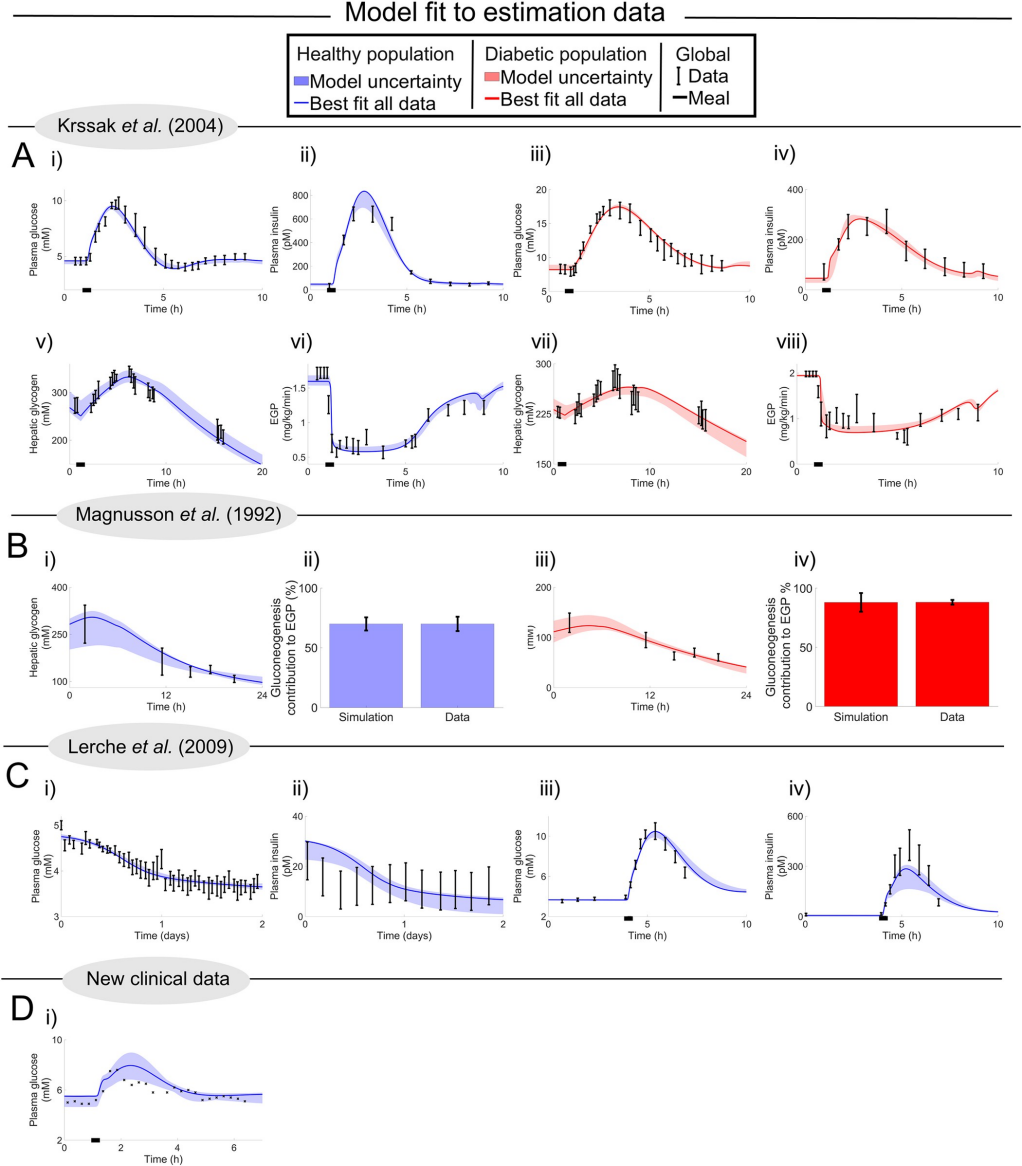
Model simulations of the four clinical studies used for parameter estimations. In all plots, data points are represented as the centre of error bars (SEM) or crosses. Simulations with uncertainty are represented by areas, and the best agreeing simulation is the line, for both healthy populations (blue) and T2D population (red). Quantitative details regarding all studies are summarised in Table D in S1 Text. All of these data are mean responses, and simulations are made for a 175 cm, 75 kg male person. (A) Krssak data [26], which describes a mixed meal response (87 g carbohydrates and 23 g protein) happening at 1 h (black bar), whereafter plasma glucose, insulin, and EGP and hepatic glycogen are measured. (B) Magnusson data [27], which describes a fasting response, following a mixed meal (98.2 g carbohydrates and 26 g protein) which happens 4 h before t = 0. (C) Lerche data [25], which describes a 48 h fast, which starts at t = 0, which is preceded by an overnight fast, and which is followed by an OGTT of 1 g carbohydrate/total bodyweight (kg). In iii) and iv), the time has been shifted, so that the meal occurs at t = 3.5 h. (D) Individual meal from new clinical study, which in this training data describes the glucose response to a mixed meal (81 g carbohydrates and 41 g protein, in total 940 kcal) at t = 1 h (black bar).

**Fig 4:**
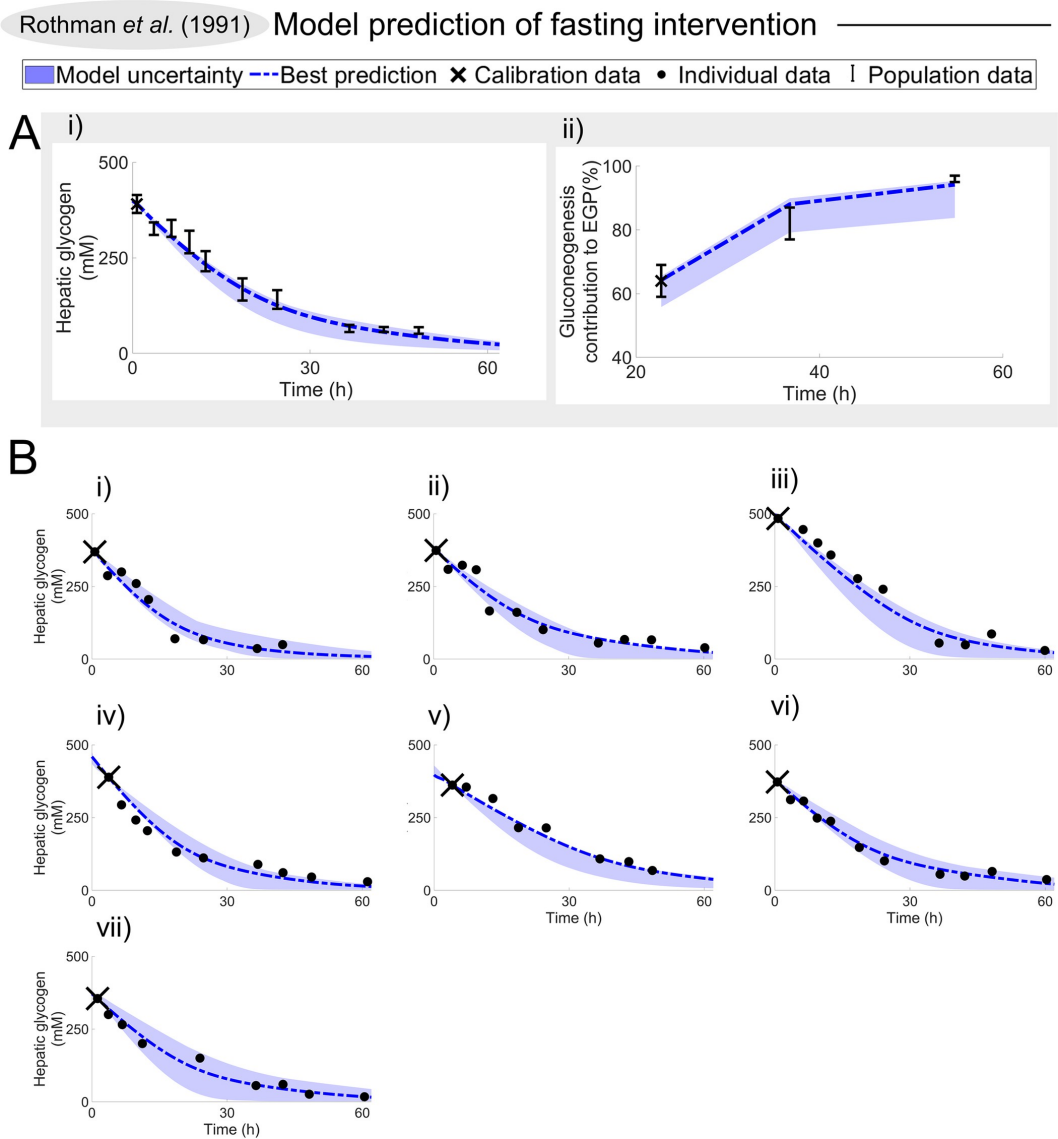
The first validation test done with the model, using the Rothman data [29]. In all plots, data values are depicted with a dot, SEM uncertainty by the error bar, simulation uncertainties are depicted by the areas, and a simulation using the model parameters that best agree with the data is depicted with the line. The first data point (also depicted with an X) is used for personalising the model, and the rest are used for validation. The study monitors the response of hepatic glycogen, and of the contribution of glycogenolysis to EGP, during a 68 h fast. (A) Prediction of hepatic glycogen levels and gluconeogenesis contribution to the endogenous glucose production on the full population. (B) Prediction of hepatic glycogen levels on an individual level.

**Fig 5:**
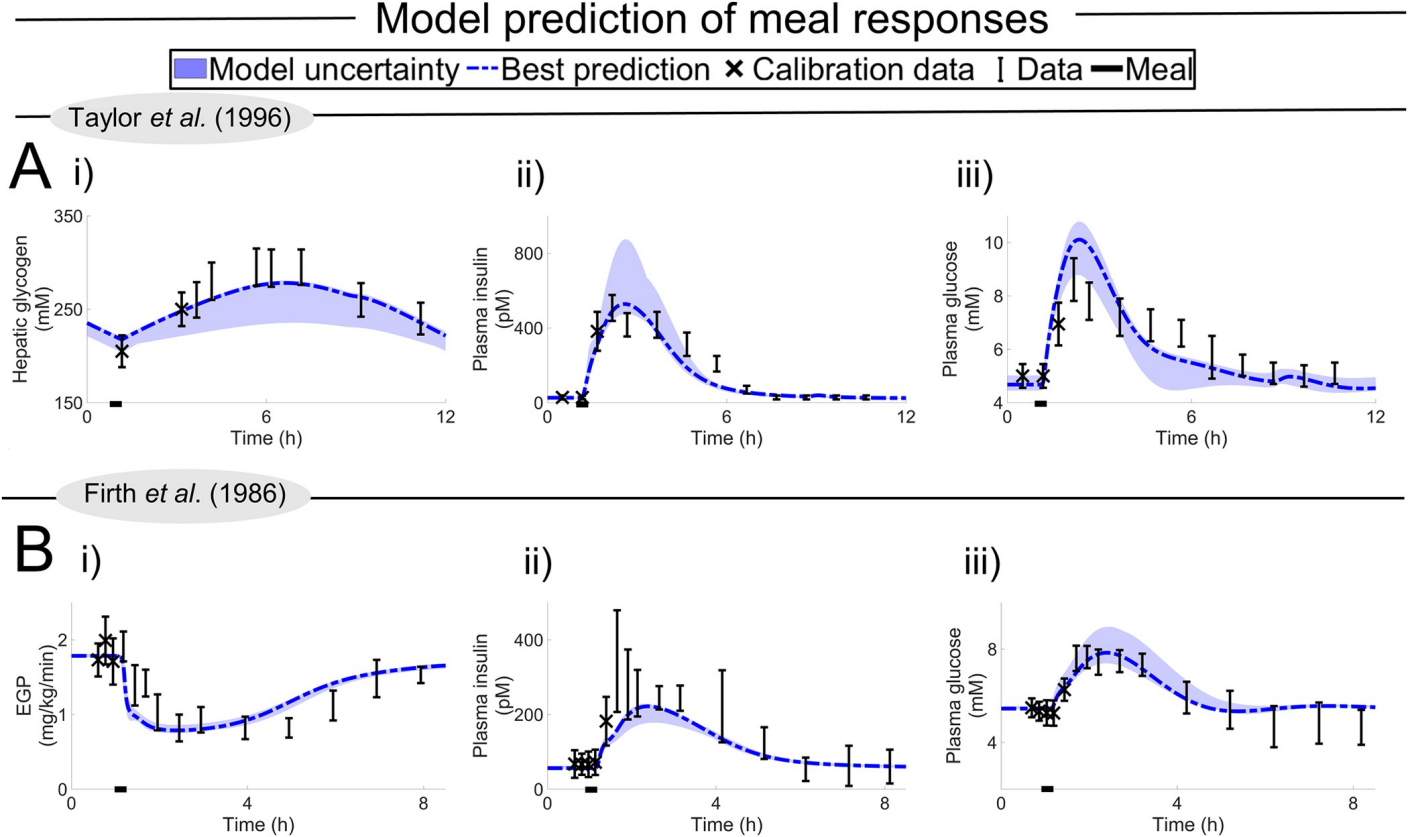
Second validation test with the model, using two clinical studies not used for training the model [30,31]. In all plots, data values are depicted with error bars (SEM), simulation uncertainties are depicted by the areas, and the simulation that best agrees with the data is depicted with the dash-dotted line. The first couple of data points (depicted with an X) are used for personalising the model, and the remaining data points are used for validation. (A) Prediction of mixed meal (black bar) from Taylor data [30], i) hepatic glycogen ii) plasma insulin iii) plasma glucose. (B) Prediction of OGTT (black bar) from Firth data [31], i) endogenous glucose production ii) plasma insulin iii) plasma glucose.

**Fig 6:**
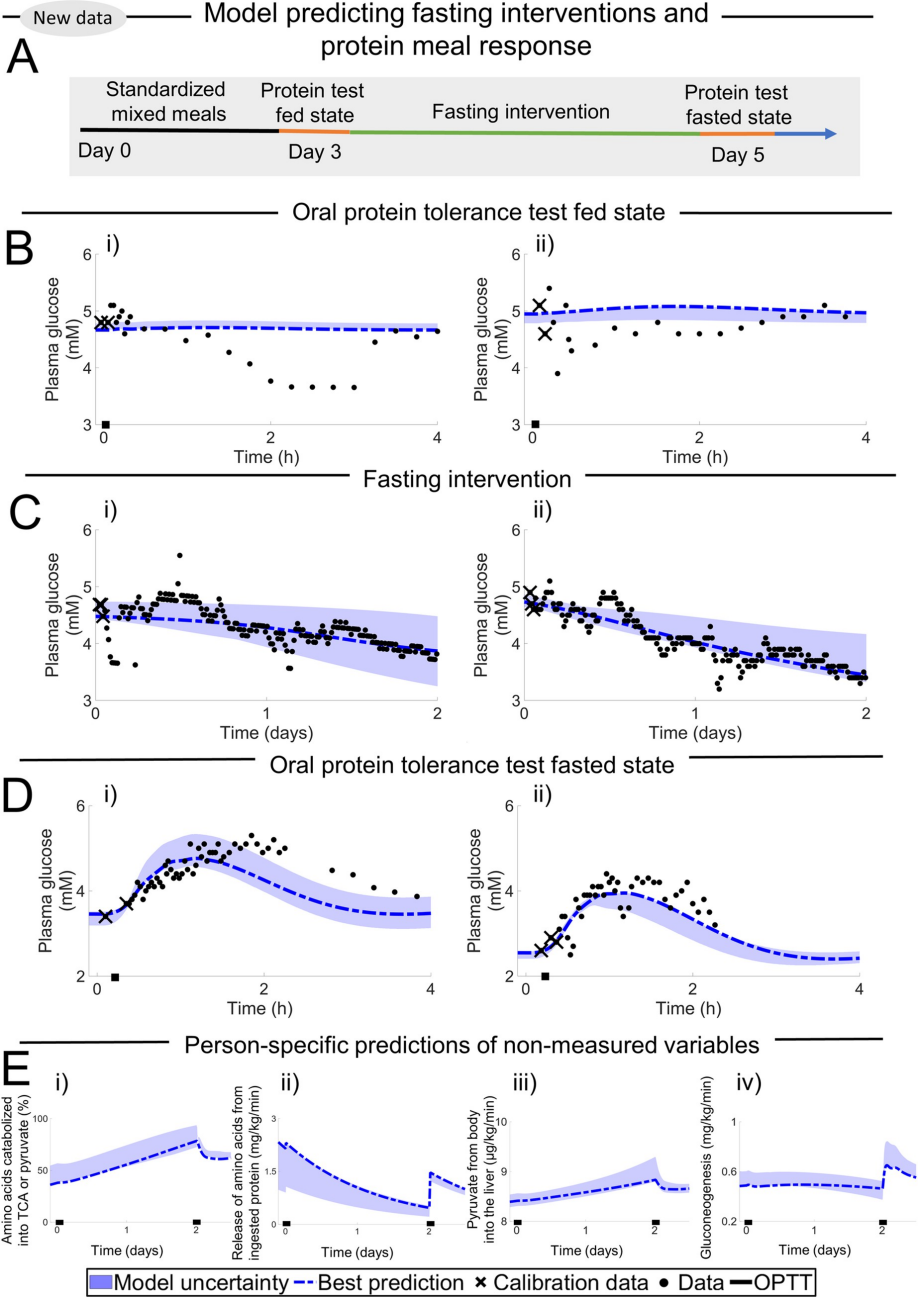
Third validation test of new data testing the model’s ability in predicting fasting intervention and metabolism of proteins. Model uncertainty is depicted with the area, the calibration data is depicted with an X, the calibrated CGM data are depicted with a dot, and OPTT event is depicted with a black line. (A) Study design. (B) OPTT response during the fed state, before the fast started. (C) Glucose levels during the 48 h fast. (D) OPTT response during the fasting state, after the 48 h fast. (E) Person-specific predictions of non-measured variables connected to protein metabolism.

**Fig 7:**
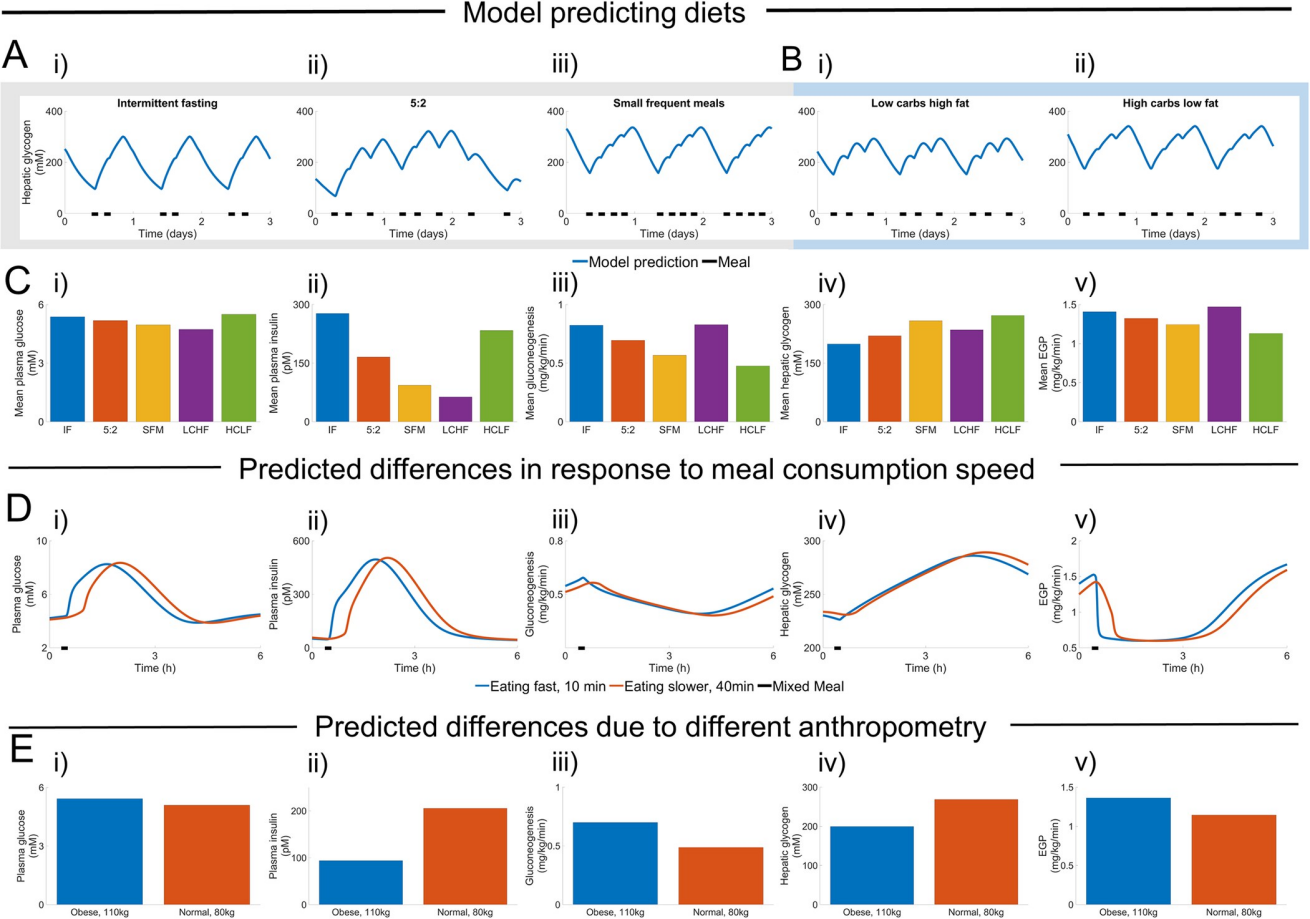
Simulations of five common diet schemes and varying different personalized variables. (A) The model considers diets with different meal frequencies, ranging from Intermediate Fasting (IF) with two meals (depicted as black bars), to 5:2 with 2–3 meals, and even SFM (with three smaller meals). These three diets are isocaloric. (B) The model can also to some extent simulate different compositions, such as LCHF (low carbohydrate, high fat diet), and HCLF (high carbohydrate, low fat diet). (C) Mean values of key variables during the second week after the diet has started. (D) Impact of changing the rate of consumption. E) Impact of changing the body weight by 30 kg, while keeping all other variables constant. All simulations in A-D were done using a male who was 180 cm, 80 kg, and in E that male either weighed 110 or 80 kg.

There are some well-established physiological models in use for specific types of clinical applications, but no available model can deal with changes between different diet compositions and fasting schemes (Fig 1E). While mathematical modelling of meal responses already started in the 1970s [17–20], a big breakthrough came with the so-called ‘Dalla Man model’ in 2007 [21]. This model is based on data from a triple-tracer experiment, where different glucose and insulin fluxes in the body were measured. A version of this model was approved by the Food and Drug Administration (FDA) in the US, as a replacement for animal experiments when certifying insulin pumps used to treat type 1 diabetes [22]. However, while there has been much work improving such models, there is still no model available that: i) can describe the physiological response to different diet compositions, *e*.*g*. containing different amounts of proteins and carbohydrates, or ii) can correctly predict the response to fasting. For these reasons, the potential of a digital twin mentioned above cannot yet be realised. In this paper, we address both of these shortcomings (Fig 2). Using four different clinical studies (Fig 3), we develop and train a new and significantly extended model. The model can predict new situations and variables, personalised responses (Figs 4–6) from both previous studies, and from a new study involving fasting and oral protein tolerance tests (OPTT) before and after 48 h fasting (Fig 6). Finally, we illustrate that the combined knowledge extracted from all of these studies can be used to predict the patient-specific impact of a variety of dietary compositions and fasting schedules (Fig 7).

## Results

### We have substantially improved and expanded the capabilities of the previous meal models

We have extended our most recent improved version of the original ‘Dalla Man model’ [23] to include a series of new features: i) intracellular metabolism in the liver, ii) long-term energy regulation via the new states for liver and kidney glycogen, iii) protein metabolism, and iv) hepatic interconversion between glucose and amino acids. Because of these changes, the new model can incorporate data and insights from a wide variety of studies, measuring *e*.*g*. fasting, glycogen, gluconeogenesis, and both hepatic and kidney endogenous glucose production. The new model has been iteratively developed and modified (Fig 1C) to be able to describe the variety of studies used in the estimation data (Fig 3). The resulting model is fully described in detail (see S1 Text), and all model files are available on GitHub (see Material and Method). The reliability of the model has been tested both quantitively and qualitatively using new data that has not been used for parameter estimation (Figs 4–6). Both estimation and validation data include various aspects of a new protein metabolism and fasting oriented study which has been designed to generate new responses not present in any of the other data: the estimation data include responses to large meals (Fig 3D), and the validation data include OPTT responses before and after 48h fasting (Fig 6). Finally, because of these new model improvements, the model can simulate physiologically-based predictions of diet responses (Fig 7), which both constitutes an additional qualitative validation, and which illustrates how the model could eventually be used in clinical applications to serve as a digital twin that provides personalised diet related metabolic insights, and thus could be used as a tool to help with metabolic dysregulation and obesity.

### The new digital twin tool can describe all original data used to develop the original ‘Dalla Man model’, while adding several qualitatively new features

The first and arguably most striking qualitative improvement in the new digital twin tool is illustrated in Fig 2A and 2B, and it concerns the ability to simulate OPTTs before and after fasting. Previous models are glucose-centric, and can only interpret OPTTs as either no input (Fig 2Ai), or using a static conversion, where the new glucose is assumed to enter directly into the bloodstream (Fig 2Aii). The new digital twin tool instead includes the biochemical description, conversion in the liver followed by endogenous glucose production (EGP), and has a negligible glucose response in fed conditions, but a clear response in fasting conditions. This changing OPTT response is a qualitatively new type of response, not seen in any of the training data, and it is quantitatively confirmed in our new clinical study (Fig 6).

Second, the new digital twin tool has not lost any critical ability compared to its predecessors. To check this, the new model was fitted to the original data used to develop the ‘Dalla Man model’ [21] (Fig 2C). In that study, healthy subjects were subjected to a meal response at t = 0.5 h (black line), and a triple-tracer protocol was used to measure a variety of variables: plasma glucose, plasma insulin, EGP, glucose rate of appearance, glucose utilisation, and insulin secretion. Compared to the previous model (orange line), the new simulations (blue line) agree at least as well with the data (error bars). The exact parameters used in the original model publication of the previous ‘Dalla Man model’ are not publicly available, and the parameters used to simulate the ‘Dalla Man model’ is estimated to fit the data in the same figure, similarly done as in the previous ‘Dalla man model’ publications [23, 24]. Finally, that the agreement between data and simulation is acceptable and supported by both visual assessment and by a *χ*^2^-test (cost = 13 < 153 = $T_{\chi 2}^{o}=F_{\chi 2}^{cdf-inv}(0.95,126)$, Material and Method).

A third important improvement to qualitative behavior to take under consideration when comparing the new model with the older ‘Dalla man model’, is the underlying mechanisms for some of the simulated dynamics, *e*.*g*. EGP. In the original ‘Dalla Man model’, the drop in EGP following a meal is explained by a phenomenological expression, which simply subtracts the rate of appearance flux from a constant value. When simulating the new model, simulations show the same drop in EGP, but instead via mechanistic mechanistic processes, e.g. involving insulin inhibition of gluconeogenesis and glycogenolysis (Fig 1D).

Finally, because of our new improved model structure and estimation procedure, the model can predict a variety of new physiological variables, not present in previous models. These newly supported physiological variables, include hepatic glycogen levels (Fig 2Di), hepatic gluconeogenesis (Fig 2Dii), and renal EGP (Fig 2Diii). None of these variables existed in either the previous ‘Dalla Man model’ or in its later developed extensions. Note that these new predictions are determined from qualitative constraints in the cost function (see Material and Method - Qualitative constraints in the cost function), and that predictions of glycogen have a smaller uncertainty after the training to the estimation data has been done (e.g. Fig 4).

### The new digital twin tool can describe estimation data for short and intermediate-term dynamics in glucose, insulin, EGP, glycogen, and gluconeogenesis, in both healthy and T2D populations

The model parameters were estimated in order to fit model simulations to data from four different clinical studies (Fig 3), three already published [25–27] (Fig 3A–3C) and a meal from our new study (Fig 3D). These studies include various variables measured in both healthy and diabetic populations, and the model can successfully describe all these studies. The data from the four different clinical studies is of four different populations and hence have a natural variance in metabolic variables such as insulin response, clearance, and resistance. These natural variances are taken into consideration in the model parameter estimation, see "Model Development" in Material and Method for details regarding parameter estimation. The agreement between data (error bars) and simulations (lines) are acceptable according to both visual assessment and a *χ*^2^-test, for both healthy data (cost = 175 < 223 = $T_{\chi 2}^{o}=F_{\chi 2}^{cdf-inv}(0.95,190)$) and for diabetes data (cost = 61 < 105 = $T_{\chi 2}^{o}=F_{\chi 2}^{cdf-inv}(0.95,83)$, Material and Method). This means that the model provides an acceptable mechanistic explanation for all estimation data [28].

The first study [26] (Fig 3A) shows mixed-meal responses for hepatic glycogen, glucose, EGP, and insulin, in a healthy (blue) and a diabetic (red) population. The meal is given at t = 1 h, represented with a black bar along the x-axis. Glucose and insulin reach a peak around 1 h, and return back to a baseline within 5 h. In contrast, the dynamics of glycogen was slower, with a peak around 2.5 h, and the concentration is still declining after 9 h. As can be seen, the model simulations (areas) replicate these qualitative features, and in general lie close to experimental data (error bars) for all variables, which is also supported by the *χ*^2^-test.

The second study [27] (Fig 3B) looks at two things—the role of gluconeogenesis and the amount of hepatic glycogen—in both healthy and diabetic populations. The subjects ate a standardised meal 4 h before the first sampling point, after which they fasted for 23 h. The contribution of gluconeogenesis to the EGP was then obtained by subtracting measured EGP from the decrease in glycogen concentration. Due to the glucose production assumption made in the study, the calculated contribution of gluconeogenesis in the model is the sum of gluconeogenesis in both the liver and kidneys divided by the total EGP. As can be seen, the contribution of gluconeogenesis is around 70% in healthy individuals, and around 88% in diabetic individuals, in both the data and model. Additionally, the glycogen levels are lower in diabetic individuals, which most likely are due to insulin resistance and a reduced insulin production. All these differences are seen both in the data and in the model.

The third study [25] (Fig 3C) again considers a fasting intervention, this time 48h, following an overnight fast. Both the model (area) and data (error bars) displayed a drop in glucose during these 48 h (Fig 3Ci). During this time, the plasma insulin concentration dropped from around 30 to around 10 pM in the model. While this drop in plasma insulin lies within the experimental uncertainty, the drop as such is not as apparent in the data. This possible discrepancy has to do with the insulin production part of the model, which is partly dependent on the current glucose concentrations in plasma. This part of the model dates back to the original ‘Dalla Man model’ [21]. After this fasting period, an oral glucose tolerance test (OGTT) of 300 kcal was performed. The OGTT was ingested at 3.5 h (Fig 3Ciii), and gave rise to a plasma glucose peak of around 10 mM, which is higher than normal in a healthy population because of the prior long fasting period. As can be seen, the model describes this unusually large OGTT response for both glucose (Fig 3Ciii) and insulin (Fig 3Civ).

The fourth and final study concerns data on a meal response, taken from a new clinical study conducted by us (Fig 3D). The reason for this addition is that all other estimation data concern a relatively small meal size (<450 kcal). Since we want the model to be able to describe meal responses during normal life, which often goes up to ~1000 kcal, we included one of the meals from the first five days in our clinical validation study (Fig 6) also in the estimation data. This additional training data describes glucose dynamics in a mixed meal of ~940 kcal. As can be seen, the plasma glucose peak is around 8 mM in both the data and model.

In summary, all of these simulation results show that the model has the ability to find a mechanistic explanation for both short- and intermediate-term dynamics in glucose, insulin, EGP, glycogen, and gluconeogenesis, in both healthy (blue) and T2D populations (red).

### The new digital twin tool can correctly predict validation data for subject-specific hepatic glycogen levels and gluconeogenesis contribution to endogenous glucose production

The first validation test done with the trained model was with the Rothman data [29] (Fig 4). In this study, they investigated hepatic glycogen (Fig 4A) and gluconeogenesis contribution to the EGP (Fig 4B) during a 68 h fast in a healthy population. The method of estimating the gluconeogenesis contribution to EGP was performed in accordance with the Magnusson study (Fig 3B). However, in the Magnusson study, the fast only lasted 23 h, while in the Rothman study the fast lasted more than twice as long. Furthermore, in the Rothman study, individual hepatic glycogen responses were reported for the seven individual subjects (Fig 4B). As can be seen, the model (area) agrees well with the experimental data (error bars) for all these observations. Here, the personalisation of each digital twin was done in two ways: i) the covariates age, weight, height, sex, and diabetes status were set to reported values, ii) the meals during the days leading up to the start of the study were optimised to agree with the initial data points, marked with an X (Material and Method). This visual assessment of the acceptable agreement was formally supported by a *χ*^2^-test (cost = 16 < 24 = $T_{\chi 2}^{o}=F_{\chi 2}^{cdf-inv}(0.95,14)$).

### The new digital twin tool can correctly predict validation data including plasma glucose, insulin, and EGP from independent studies

The second validation test was conducted in order to test the model’s ability to predict meal responses (Fig 5) [30,31]. The data represented in Fig 5 includes two different studies: one mixed meal (Fig 5A) and one OGTT (Fig 5B). These two studies were chosen because they measure not only plasma glucose and insulin, but also one additional variable that has to do with the new additions to the model: hepatic glycogen (Fig 5A) and EGP (Fig 5B). Considering that the size and meal content are different in both studies, the response in glucose and insulin is qualitatively similar in both studies. EGP declines in response to the OGTT and then slowly goes back to normal, on a timescale that is comparable to the glucose and insulin meal responses. In contrast, the hepatic glycogen increases in response to the meal with a peak after about 5–7 h, and hence works on a slower timescale than the other variables. All these observations are correctly predicted by the model, both qualitatively and quantitatively. As before, this visual assessment is supported by a *χ*^2^-test, both for the mixed meal (cost = 47 < 50 = $T_{\chi 2}^{o}=F_{\chi 2}^{cdf-inv}(0.95,35)$) (Fig 5A) and for the OGTT (cost = 39 < 60 = $T_{\chi 2}^{o}=F_{\chi 2}^{cdf-inv}(0.95,44)$) (Fig 5B). Nevertheless, the digital twin tool does predict a slightly lower synthesis of hepatic glycogen from the mixed meal (Fig 5Ai) compared to the experimental data. This slight discrepancy may be a product of underlying differences between the population used in the training of the model and the population used in the Taylor study. Note that personalisation of the models is done in both Figs 4 and 5, and that the digital twin can use all variables available for this because of its physiological nature. The data used for personalisation are marked with an "X", and all other data points are used for validation (see Material and Method –Model personalisation for more info).

### The new digital twin tool can predict fasting interventions and protein metabolism both in a fed and unfed state as validated by a new study

To further test the capabilities of the model, a new dataset was collected that tested a new type of prediction, not present in any of the other datasets. More specifically, the new study examined the response to an OPTT before and after a 48-h fasting (Material and Method). These combined OPTT and fasting responses are highly central predictions for the new additions to the digital twin model. The reason for this centrality is that these predictions concern the interplay between glucose and protein metabolism and how this interplay is impacted by glycogen and fasting. These interplays and regulations were not present in previous models (Figs 1E and 2A).

Three different predictions were central to the design of the clinical study (Fig 6A). Firstly, plasma glucose will decrease during the 48-h fasting (Fig 2Aiii). This prediction is different compared to the corresponding prediction of the prior meal response models (Fig 2Ai and 2Aii), and it is supported by data (Fig 6C) for both subjects. In contrast, the second and third predictions concern the OPTT responses before and after this fasting period, and these predictions have not been seen in any training data. The second prediction is that the first OPTT response, before the fasting, will not give rise to any noticeable response in plasma glucose concentration, and this is what is seen in the data (Fig 6B). In contrast, the third and final prediction is that the same OPTT will give rise to at least 1 mM increase in plasma glucose after the fasting, and that this rise will remain above the glucose values before the OPTT for at least 2 h (Fig 6D). As can be seen, these observations are clearly fulfilled by the data. Since we are now in a digital twin setting, with subject-specific responses, each data point comes without an associated uncertainty, and a direct *χ*^2^-test is not possible.

To exemplify the model’s ability to predict non-measured variables, four different person-specific predictions were made concerning the following: the percentage of released amino acids from the digestive system that are metabolised into tricarboxylic acid cycle (TCA) cycle components or pyruvate (Fig 6Ei), total release of amino acids from ingested protein (Fig 6Eii), the rate of appearance of pyruvate from internal sources (Fig 6Eiii), and gluconeogenesis (Fig 6Eiv). The model predicts an increasing ratio of amino acids used for energy regulation as the fasting intervention continues and a decreasing ratio when consuming the OPTT in a fed state. Furthermore, the model predicts a slow release of amino acids from the stomach in order to balance the need for energy over time. During the fasting intervention, the internal supply of pyruvate from the body increases, which is mechanistically explained by muscle degradation, and a decrease of the internal supply of pyruvate when consuming protein in a fasting state (t = 2). Most importantly, the model predicts differences in protein metabolism in a fed state and a fasting state, where the spikes of gluconeogenesis in fasting and fed states (t = 0 and t = 2, Fig 6Eiv) have significantly different magnitudes.

In summary, our new clinical study validates a dynamic and qualitatively novel prediction made by the model.

### The new digital twin tool illustrates physiological accurate behaviour of the human metabolism including insulin degradation from the liver and plasma, organ-specific glucose uptake, and renal glucose production

Unlike the original ‘Dalla Man model’, the new model can produce organ-specific predictions (Fig 2E), such as organ-specific glucose uptake; contribution of renal and liver glucose production to EGP; and the ratio of how much insulin is cleared by the liver compared to clearance by the rest of the body. The model is able to simulate reasonable organ-specific glucose uptake both in a post-adsorptive state and a postprandial state. In a post-adsorptive state, such as overnight fasting, organ-specific glucose uptake ratios have been measured to be approximately 40–50% in the brain, 15–20% in muscle, 10–15% in the liver, and 5–10% in the kidneys [32]. These data are in good alignment with the model simulations, which lie within these uncertainty regions for all organs (Fig 2Ei). The model can also describe renal glucose production (RGP) in qualitatively accurate proportions compared to hepatic glucose production (HGP). RGP produces approximately 5–10% of all endogenous glucose in a post-adsorptive state and 10–15% in a postprandial state [32], which is well described by the model (Fig 2Eii). Finally, the model is also able to simulate a realistic insulin rate of disappearance produced by the liver, compared to the rest of the body (Fig 2Eiii). Note that these experimental data have a high degree of variation, with values ranging from 80% [33] to 50% [34]. Also note that all organ proportion data referred to come from a variety of different studies, most of which differ to the model simulations considered herein.

### The new digital twin tool predicts short and long-term responses to common diet schemes

To illustrate the potential of our digital twin tool, we simulated the impact of changing the meal frequencies (Fig 7A and 7C), meal compositions (Fig 7B and 7C), ingestion speed (Fig 7D), and body weight (Fig 7E). The change in diet was performed using a three-week simulation protocol. The first week is used to reach a steady state using a standardised diet. In the second week, a new diet is introduced, which eventually reaches a new steady state behaviour. The third week is the week plotted and analysed in Fig 7C. The plots in Fig 7A and 7B depict three days during this third week, to illustrate the daily variations. Note that one of the diets, the 5:2 diet, has different numbers of meals and different meal sizes during the five normal days (2560 kcal/day), compared to the two restricted days (600 kcal/day). Note also that all diets in Fig 7A are isocaloric, consuming an average of 2000 kcal/day. This is to some extent also the case in Fig 7B, comparing LCHF and HCLF (see Limitations in Discussion). In all simulated isocaloric diets, *i*.*e*. i) intermittent fasting, ii) 5:2, and iii) SFM, macronutrient profiles were 45% carbohydrates, 27% protein, and 27% lipids of the total energy intake. The HCLF diet was simulated with its energy content derived from 60% carbohydrates, 25% protein and 15% fat, while the LCHF diet was simulated with half the amount of carbohydrates in comparison. In the comparison of all diets, five key variables were compared (Fig 7C). In all simulations of Fig 7 the macronutrients were chosen to emulate popular diets while simulating meals close to the individual meals in the estimation data, and close to the situations validated in Figs 4–6.

Finally, variables such as meal consumption speed (Fig 7D) and different anthropometry (Fig 7E) were changed in between simulations and then compared with each other. The model predicts that a high ingestion speed will lead to a faster response, but there was no noticeable impact on amplitude of the key variables. In the simulations, a lower body weight implies a higher insulin response, because the meal size is higher per body weight. This change in insulin dynamics implies higher glycogen levels, and lower EGP, which in turn inhibits gluconeogenesis. Note that our new model does not have an insulin resistance term that depends on body weight, and that all other model parameters are kept the same in Fig 7E. In a real weight-loss study, it is likely that there will be an impact on insulin resistance. However, our simulations illustrate how one can isolate the function of the different components in a way that is not possible *in vivo*.

## Discussion

We present a novel offline digital twin technology that integrates many different studies—which all contain different, non-connected, and complementary information about human metabolism—into a single, quantitative, robust, and coherent picture. The foundation for this new twin technology is a multi-timescale, mechanistic, mathematical model (Fig 1A and 1D, and Fig A in S1 Text), which describes the diet response of both the intracellular liver metabolism and the organ-organ crosstalk, and which can be personalised and updated when new data becomes available. The newly added and improved features in the model are centred on protein dynamics, intracellular liver metabolism, and the long-term regulations involving glycogen (Fig 1C, blue; Fig A in S1 Text, blue). Two qualitative improvements (Fig 2A) illustrate the functionality of the new model mechanisms, and they were quantitatively tested in our new clinical study (Fig 6). The improvements are: i) glucose decreases over long-term fasting, since EGP comes from finite intracellular sources (Figs 2Aiii, 2B, and 6A), and ii) protein metabolism differs between a fed and a fasting state, since the energy status in the body regulates whether protein should be used for gluconeogenesis or anabolic processes (Figs 2A, 6B and 6C). The quantitative performance of the model is shown by *χ*^2^-test, which the model passes both for the training data (Fig 3), and the independent validation data (Figs 4–6). Finally, we demonstrated the potential future usefulness of our new digital twin, by simulating the impact that a variety of different diets is expected to have on key variables, such as mean plasma glucose, plasma insulin, hepatic glycogen, gluconeogenesis, and EGP. This demonstration provides a basis for understanding the consequences of these different diets (Fig 7). Many of these data and predictions (Figs 3D, 4B, 6 and 7) are subject-specific while others are population-specific (Figs 3A–3C, 4A, and 5). We believe that this twin could become useful, not only for personalised diet design, but also to improve patient understanding, motivation, compliance, and outcome [12].

### The new model may be used to estimate non-measured variables in existing and future studies, and thereby deepen our understanding of physiological processes and regulations

The new novel digital twin model can predict the response of both measured and non-measured variables. Measured variables correspond to known data and can be used to assess the model’s descriptive (Fig 3) or predictive (Figs 4–6) ability, depending on whether the data is used for training (Fig 3) or testing (Figs 4–6) the model, respectively. Non-measured variables in a specific study setting show the behaviour of the model in variables for which we have no data. In other words, non-measured variables are predictions with the model. Examples of such non-measured variables are shown in Fig 2D, where we see gluconeogenesis, hepatic glycogen, and renal EGP dynamics; in Fig 2E, where we see the relative contributions of the different organs to glucose and insulin clearance and production; in Fig 6E, where we predict the release and usage of amino acids from the digestive system, the rate of appearance of pyruvate from internal sources and gluconeogenesis; and in Fig 7, where we predict the response to different diets. Such predictions are possible and interesting to consider because the new model has already demonstrated its ability to predict new variables (Figs 4–6), and because it utilises information from other similar studies, where these variables have been measured. Finally, the model structure as such also provides valuable information, since it includes a sufficient set of mechanisms that can describe all these studied dynamics. For instance, in this model, the only long-term regulation variable is glycogen, which together with the short-term insulin regulation, provides all the regulations included in the model. This means that, at least for these meal responses, the high complexity in metabolic regulation seen using gene expression, bioinformatics, and other omics technologies, in the end, boils down to one major short-term and one major long-term regulation, which correlate with insulin and glycogen levels, respectively.

### The predictive ability of the new model has been tested both quantitatively and qualitatively in a variety of ways

The quantitative assessments of the model predictions are shown in Figs 4–6, where the data (error bars) and simulations (lines and areas) are plotted together. Fig 4 shows such comparisons on an individual level, in a study where the subjects fasted 60+ h. This is impressive because the model has only been trained on up to 23 h fasting, and only on average population data. Fig 5 shows validation tests using a mixed-meal and a OGTT study, which examines plasma glucose, plasma insulin, hepatic glycogen, and EGP dynamics [30,31]. Fig 6 shows the new clinical study, which validates the qualitatively new prediction (Fig 2A) that an OPTT gives a negligible response in plasma glucose levels before fasting, but a 1–2 mM response after 48 h of fasting. This last prediction is important because this combination of OPTT and fasting is a fundamentally new type of observation, not present in any of the existing data used for either training or validation. The prediction is also reasonable, given basic biochemical knowledge: when you have fasted long enough, the liver metabolism switches to produce glucose from proteins, and when new proteins become available, hepatic glucose production goes up. However, while this strengthens the intuition behind why the prediction is reasonable, a quantitative model is needed to be able to predict how long one must wait, and how big the OPTT must be to produce a plasma glucose response of a certain magnitude. Note also that the organ proportions for glucose uptake in fasting conditions (Fig 2Ei), EGP production (Fig 2Eii), and insulin degradation (Fig 2Eiii) are all in good agreement with the data [32,33,35]. Finally, all the quantitative assessments above are clear both from a visual comparison between simulations and data, as well as from formal and statistical analysis using a *χ*^2^-test.

### The new model quantitatively predicts short- and intermediate-term responses to different diets

To illustrate an additional usage of our new digital twin model, and also to provide additional validations of model predictions, we simulated short- (hours) and intermediate-term (up to three weeks) responses to various dietary and fasting strategies (Fig 7). To isolate the impact of the dietary strategy as such, all compared options are isocaloric, seen over a week, to the extent that the model can obtain this. Using the mathematical model, we could for example compare the effect of diets with higher (SFM) and lower (Intermittent fasting and 5:2) meal frequencies (Fig 7A). We found that higher meal frequencies led to clear reductions in mean plasma insulin levels, but to small changes in mean plasma glucose, which is in agreement with several previous studies [36–38]. Note that this and the other diet-change comparisons with literature are only qualitative, *i*.*e*. not quantitative. We can also predict additional impacts, not measured in those previous studies, for example that higher meal frequency diets give higher mean hepatic glycogen levels, and that less of the ingested protein is converted into glucose. This prediction is reasonable, since less frequent meals lead to more extended periods of fasting when glycogen is depleted, and gluconeogenesis is turned on. Similarly, diets containing high carbohydrates and lower protein (HCLF) resulted in higher hepatic glycogen, plasma insulin, and plasma glucose levels, which agrees with existing clinical studies [39, 40]. Given our results, one can also argue that diets such as LCHF, which lowers both average insulin and glucose levels, may be beneficial for treating diabetes and cardiovascular diseases, and short-term clinical studies indicate that LCHF does indeed lower fat levels in both the liver and blood [41]. However, such clinical predictions should be made with great care, since several critical factors are still missing from the model. One such important missing factor is crosstalk between lipid and glucose/protein metabolism, which is currently not available in the proposed digital twin, or in any other similar data-driven model. Furthermore, the model does not consider important long-term effects of prescribing the diets described here in clinical practice, since real clinical effects also involve impact on adherence and relapse risk, impact on appetite, etc., which are not included in the model. It may be because of such complicating factors, that long-term studies of different diets show few clear results regarding which diet is best, and that the effects of more well-controlled shorter studies show clearer effects. In any case, to be able to simulate the short- and intermediate-term impact of following a diet may be useful to explain to a patient why we believe that the prescribed diet may be beneficial, and such simulations could increase patient understanding, and motivation to adhere to the diet.

### Limitations, strengths, and key assumptions in the new model compared to other available meal simulations models

Meal simulation models have been developed since the 1970s [17,20], but even though many developments have taken place since then, there does not exist a previous model that can incorporate and predict/describe the data in the studies we have utilised herein (Fig 1E). An important step forward was the ‘Dalla Man model’ [21], which made use of triple-tracer data to construct a more physiological model, based on measurements estimating fluxes such as EGP, glucose uptake, and rate of appearance from the intestines. The new model can describe all of those data (Fig 2C, grey). Later models by us [23,42] and others [43,44] have produced more refined versions, which also describe glucose uptake subdivided into the different organs. Our model maintains this capacity and describes organ-specific insulin clearance and EGP contributions (Fig 2E). Some other related previous models have instead focused on the detailed metabolism of the liver [45–47], but such models have had no realistic meal response part. There are also various detailed models which have had little to no quantitative agreement with data, especially independent validation data [44,48]. Also, neither of these meal response models has described more long-term changes, such as the build-up or breakdown of glycogen over days. There are more long-term models involving glycogen [49], but these models do not describe meal responses.

There are many shortcomings and assumptions in the model, many of which are due to a lack of the needed clinical data. For instance, the model does not include metabolism and crosstalk with lipids. This is a shortcoming in the context of simulation of LCHF, since the ingested fat is just assumed to be consumed, with no consequence for other dynamics. This shortcoming also means that an extension of these dynamics to more long-term dynamics, involving factors such as weight change, is not yet possible. There is currently no other model that describes such a crosstalk, and to develop such a glucose-protein-fat metabolism model is an important next step for the field, and it will bring many of the benefits hinted at herein to an even more realised potential. Also, many of the intracellular metabolic reactions are highly simplified. To add more detailed intracellular models, which also incorporate *in vitro* experimental data, is another important future research direction. We have previously carried out such multi-level expansions for insulin signalling in the adipose tissue [23, 50], and those more detailed adipose tissue models can also be included in this model. Furthermore, the meals in the clinical studies used as estimation data are small (< 1000 kcal) and we have experienced that the model has problems scaling between meal size used in the estimation data and larger meals (> 1000 kcal). Non-physiological behaviours such as oscillations have been observed when simulating non-supported situations that are not a part of the estimation data. Herein, we advise users to simulate situations close to estimation data. In future work we will aim to further expand these supported situations. Finally, the personalisation of the digital twin model has only been demonstrated on a small number of individuals (Figs 4B and 6) and on a limited number of different sub-populations (Figs 4A and 5). These data thus only cover a small subset of the total variation seen in an entire population. It is likely that there are individuals and sub-populations with behaviours that our current model cannot correctly predict.

Despite these and other shortcomings with the presented model, it shows the way towards a more all-encompassing meal simulation paradigm that goes beyond glucose-insulin dynamics, towards more complex meal responses and long-term dynamics, and which lays the basis for a new type of physiological digital twin technology. Such digital twin technologies have previously been developed for other processes, such as blood pressure and flow [13,14]. For complex meal responses involving a variety of metabolites, only machine learning models have previously been available [12]. Using our new type of physiological models, we can go beyond such ‘black box’ machine learning models to explainable artificial intelligence. This opens the door to conveying physiologically based metabolic insights, which in turn could lead to increased patient motivation and compliance with dietary prescriptions, which in turn could help prevent metabolic and cardiovascular diseases.

## Material and method

### Ethics statement

The study was conducted with the approval of the Swedish Ethical Review Authority (Etikprövningsmyndigheten), DNR-2021-02668.

### Mathematical modelling

The general mathematical modelling methodology used is similar to many of our previous papers [51, 52], and below a short method summary is outlined. The mathematical analysis, model simulation, numerical optimisation of model parameters, was carried out using MATLAB 2019b and the IQM toolbox [53]. All code is available at our GitHub repository, see: https://github.com/OscarSilfvergren/Digital-twin-predicting-diet-response-before-and-after-long-term-fasting.git.

The model is based on *ordinary differential equations* (ODEs) with the general form:

$$\frac{dX}{dt}=f(X,t,q,u),$$ (1A)

$$X(t_{0})=X_{0}(q)$$ (1B)

ŷ=g(X,t,q,u), (Eq 1C)

where *X* denotes a vector of state variables usually corresponding to concentrations of given system components; the functions *f* and *g* are non-linear smooth functions; *q* is a vector of model parameters (rate constants, scaling constants, etc.); *u* is the input signal corresponding to the meals ingested, as specified in the experimental data; *X*(*t*_0_) denotes the initial condition value *X*_0_(*q*), which are dependent on the model parameters *q*; and ŷ is the simulated model output. Parameter estimation was done by quantifying the model performance, using the model output ŷ to calculate the traditional weighed least squares cost function written as

$$V(q)=\sum_{i,j}{\left(\frac{y_{i}(t_{j})-{ŷ}_{i}(t_{j,}q).}{SEM_{i}(t_{j})}\right)}^{2}$$ (2A)

$$SEM=\frac{\sigma_{i}(t_{j})}{\sqrt{n_{i}(t_{j})}}$$ (2B)

where *y*_*i*_(*t*_*j*_) denotes the measured datapoint, for a specific experimental setup and variable *i*, at time point *j*; where ŷ_*i*_(*t*_*j*_,*q*) denotes the corresponding simulation value; and SEM is the standard error of the mean, which is the sample standard deviation, *σ*_*i*_(*t*_*j*_), divided by the square root of the number of repeats, *n*_*i*_(*t*_*j*_) at each time point. The value of the cost function, *V*(*q*), is then minimised by tuning the values of the parameters, typically referred to as parameter estimation. The parameter estimation was done using the particle swarm optimisation algorithm from the MATLAB global optimisation toolbox. The global optimisation was done using an initial swarm size of 2000 and the stopping criteria of the optimisation was determined through a combination of maximum stall iterations [20] and a tolerance limit (10^−6^). The global optimisation search was done several times to increase parameter variability (Table E in S1 Text).

In order to evaluate the new model, we performed a *χ*^2^-test for the size of the residuals, with the null hypothesis that the experimental data had been generated by the model, and that the experimental noise was additive, independent, and normally distributed [28]. We did not explicitly test these assumptions regarding distribution and nature of the noise. In practice, the cost function value was compared to a *χ*^2^ test statistic, $T_{\chi 2}^{o}$. The test statistic value is given by the inverse *χ*^2^ cumulative density function,

$$T_{\chi 2}^{o}=F_{\chi 2}^{cdf-inv}(1-\alpha ,v)$$ (3)

where $F_{\chi 2}^{cdf-inv}$ is the inverse density function; α is the significance level (α = 0.05, was used); and *v* is the degrees of freedom, which was equal to the number of data points in the training dataset (273 in total, all time points over all experiments). In practice, the model is rejected if the model cost is larger than the *χ*^2^-threshold ($T_{\chi 2}^{o}$).

### Qualitative constraints to the optimisation

To avoid non-physiological behaviours, we also added several *ad hoc* requirements to the optimisation. These requirements, if not fully filled, would increase the cost function (Eq 2A) with a constant term, which is present during the optimisation, but not during the *χ*^2^-test. These *ad hoc* requirements were based on prior knowledge represented in Fig 2E:

- No negative reaction flows, state values, or variables.
- No plasma insulin above 3000 pmol/L.
- Renal glucose production is not permitted to exceed 40% of the total glucose production [35].
- Insulin degradation in the liver is not permitted to decrease below 40% the total degradation [33].
- Glucose uptake ratios to the different organs, both during postprandial and overnight fasting, should be reasonably proportionate (Fig 2Ei) [32].
- Gluconeogenesis contribution to the EGP is not allowed to exceed 100%.
- A population in a fed state has hepatic glycogen between 200 and 350 mmol/L.
- A population in an unfed state has hepatic glycogen below 100 mmol/L.

### Seven existing clinical studies used for estimation and validation

A total of seven existing clinical studies [21, 25–27, 29–31] were collected to train (Fig 3) and validate (Figs 4 and 5) the model. The studies used are presented in Fig 1C. The basic features of these studies are described in the legends to Figs 3–5, and more details are summarised in Table C in S1 Text. For a full description of the methodologies used, we refer to the individual papers. Most of the data used was available in the supplementary material of [45], and the remaining data were extracted from the figures in the papers. In the data of Lerche *et al*. (2009) [25] the SEM of insulin in two data points was particularly small and to avoid too high a weight being given to those data points, their SEM was thus replaced with the mean SEM during the fasting intervention.

### New study: OPTT before and after two days of fasting

While there are many highly informative studies available in the literature, we found a critical lack of data for certain combinations of diets, meal sizes, and fasting schedules. Therefore, a new study was conducted (Fig 6A), to provide some critical information needed to train the model, and to allow for a completely independent validation of critical predictions with the model (Figs 2iii, and 6). Since our model is a digital twin technology, with individual models for each subject, it is more important that N is high for the number of data points, and an N = 1 study is in principle sufficient regarding the number of subjects. For this reason, we performed a study with only three non-diabetic subjects (N = 3, ages = [24,29,42] years, weights = [80,87,85] kg, heights = [1.78,1.79, 1.85] m). To remedy the need for a high time-resolution, we combined a wearable continuous glucose monitor (CGM) (Abbott, *FreeStyle Libre 2*), with a regular sampling of blood glucose values using the associated Abbott kit, to allow for calibration of the CGM values. The CGM sampled every 15 minutes throughout the entire 14-day study, and during the critical OPTT response, we collected blood samples every two to four minutes. During the first three days of the study, a normal non-controlled diet was followed, but some meals were carefully measured, to allow for calibrations of the digital twins. Importantly, at least one of these meals was large, ~1000 kcal, since such large meal responses are lacking from all other clinical studies included in the analysed data. For this reason, one such large meal response was included in the estimation data (Fig 3D). At the end of day three, a regular dinner was ingested at 19.00, and the first OPTT was taken at 23.00 (132.5 kcal, 25.55 g proteins, 2.6 g carbohydrates). After this, a 48 h fast followed (only water and black tea allowed), and at 23.00 on day five, a new OPTT was taken, using the same protocol. After this second OPTT, CGM values were collected for another nine days, until the sensor expired.

### Model development

Our new model is physiologically based, and thus the process of developing and training the model to data differs from that used in machine learning applications. For instance, the digital twin model equations are structured to describe physiologically based meal responses. These descriptions include flows between organs, metabolic reactions, and how these two are regulated in humans. In order to describe these flows and reactions, the digital twin model has three different types of parameters: i) *general parameters (57 parameters)*, which do not vary from individual to individual, or population to population, but which are different between diabetics and non-diabetics; ii) *person-specific parameters (5 parameters)*, which are fitted specifically to the specific population or individual; and iii) *model inputs*, which are not optimised, but are given by the experimental setting (when specific meals are given, and what their content is) or by known demographics (sex, weight, height, diabetes status).

These three sets of parameters are treated and optimised in different ways. i) The general parameters are set through a global optimisation algorithm, used to fit the model to the estimation data (Fig 3). The parameter values obtained from the optimisation algorithm are restricted within pre-declared bounds (Table D in S1 Text) to avoid extreme values and thus reduce non-physiological model dynamics. ii) The person-specific parameters allow for variations between the populations and individuals, and describe differences in insulin production, insulin clearance, and insulin resistance. These variations are introduced as modifications to the corresponding general parameters derived from fitting to estimation data. Further details regarding the personalisation parameters are given below. iii) The model inputs are not optimised.

When the optimisation algorithm searches for model parameters that provides simulations close to data, the agreement to data was calculated in relation to the corresponding SEM and all parameters with an agreement with data below the previously explained *χ*^2^-threshold were saved. Parameter values derived from fitting simulation to the diabetic population of the estimation data (red, Fig 3) were saved separately from the healthy population (Fig 3, blue). The saved parameters will predict slightly different metabolism in response to the same diets or individual meals due to the difference in the parameter values. The corresponding differences between model simulations result in the estimated model uncertainty. The parameter estimation was done multiple times, from different starting points, to find as many different parameters as possible. This approach corresponds to Step 1 of the core prediction approach presented in [54]

### Model personalisation

The model can be personalised, from predicting the entire population of the estimation data (Fig 3) into population-specific prediction (Figs 4A and 5) or into individual specific predictions (Figs 4B and 6). The personalisation is done through a calibration that will improve prediction precision to a population or an individual. Hence, the calibration is optional with respect to simulating general metabolism (Fig 7), but usable when making predictions of specific populations or individuals. The calibrations used to personalise the predictions may be separated into three categories: i) declaration of anthropometry (gender, weight, length), and diabetes status, ii) calibration to basal values (which creates a plausible diet five days prior to the simulation start), and iii) calibration based on data (insulin production, insulin clearance and insulin resistance).

In the first category, declaration of anthropometry, the model will scale blood volumes based on the declared information (gender, weight, length). The total blood volume is estimated to be within ±30% of equations published in [55] and the blood in the liver is assumed to be 13% of the total blood volume [56]. Furthermore, in this user declaration, the user declares whether the prediction should be of a diabetic or a healthy user, where the parameters used for the prediction will either be using the parameters derived from the diabetic population in the estimation data (red, Fig 3), or the parameters derived from the healthy population in the estimation data (blue, Fig 3).

In the second category, calibration to basal values, the user may declare a specific start of the human metabolism simulation. The information provided is used partly to fit two person-specific parameters describing basal glucose and insulin levels, and partly to create a diet simulated prior to the model simulation start. The created diet will therefore provide values close to the experimentally observed initial values, before the first meal is taken (the initial data point marked as "X" in Figs 4–6).

In the third category, calibration based on data, three model parameters that allows for variation between populations are fitted to the supplied calibration data (marked as "X" in all figures). The third calibration is important because even though the model will use parameters based on the declaration of either “healthy” or “diabetic”, there are still fundamental differences between populations and individuals within that group. The model can thus be calibrated where we allow variation in insulin production, insulin clearance and insulin resistance to make better personified predictions on an individual or population-specific level.

When making model predictions, the values of the 57 general parameters that describe flows, regulation and rate of change are the same as the parameter values derived from the estimation data. The five person-specific parameters obtained from parameter fitting to estimation data (Fig 3) may be kept as they are, to provide general metabolic insights (Fig 7), or may be calibrated to a specific person’s calibration data (marked with an X), to make person-specific predictions (Figs 4–6).

## Supporting information

S1 Text **Fig A in S1 Text. Illustration of the complete model**. Rectangles represent states, text with no squares are model reactions, and flows into Ø are flows leading out of model. Red colour represents the old model and blue the new. Dotted lines represent positive dependency regulations and dotted lines ending with a perpendicular head represent inhibitions. **Table A in S1 Text. List of model states, its corresponding units, and a short description of what it represents.** Initial value of simulations is represented as **θ**_**0**_. **Table B in S1 Text. List of parameters and the starting guess, θ**_**0**_**, of each parameter in the parameter estimation. θ**_**0_Healthy**_ is the parameter starting guess for healthy populations and **θ**_**0_T2DM**_ is for diabatic populations. The categorisation observed in Table B in S1 Text is based on the location of corresponding physiological mechanism, and parameters of flows between categories are specified by the category of the start of the flow, e.g. the parameter representing glucose diffusion between blood and tissue, k_1_, is specified in the blood category. **Table C in S1 Text. Summary of clinical studies used to evaluate model. Table D in S1 Text. List of restriction bounds for optimisation. Θ**_**low_Healthy**_ is the lowest bound and **Θ**_**high_Healthy**_ is the highest bound when optimising parameters for the healthy population. **Θ**_**low_T2DM**_ is the lowest bound and **Θ**_**high_T2DM**_ is the highest bound when optimising parameters for the diabetic population. **Table E in S1 Text. Uncertainty of model parameters** *Θ*_*min_Healthy*_ is the lowest parameter value obtained in the fitting to healthy population and Θ_max_Healthy_ is the highest. Θ_min_T2DM_ is the lowest parameter value obtained in the fitting to diabetic population and Θ_max_Healthy_ is the highest.
(DOCX)

## References

[1] Ajala O, English P, Pinkney J. Systematic review and meta-analysis of different dietary approaches to the management of type 2 diabetes. The American Journal of Clinical Nutrition. 2013;97(3):505–16. doi: 10.3945/ajcn.112.042457 23364002

[2] Ge L, Sadeghirad B, Ball GDC, da Costa BR, Hitchcock CL, Svendrovski A, et al. Comparison of dietary macronutrient patterns of 14 popular named dietary programmes for weight and cardiovascular risk factor reduction in adults: systematic review and network meta-analysis of randomised trials. BMJ. 2020;369:m696. doi: 10.1136/bmj.m696 32238384 PMC7190064

[3] Ganesan K, Habboush Y, Sultan S. Intermittent Fasting: The Choice for a Healthier Lifestyle. Cureus. 2018;10(7):e2947–e. doi: 10.7759/cureus.2947 . 30202677 PMC6128599

[4] Chawla S, Tessarolo Silva F, Amaral Medeiros S, Mekary RA, Radenkovic D. The Effect of Low-Fat and Low-Carbohydrate Diets on Weight Loss and Lipid Levels: A Systematic Review and Meta-Analysis. Nutrients. 2020;12(12):3774. doi: 10.3390/nu12123774 . 33317019 PMC7763365

[5] Dashti HS, Mogensen KM. Recommending Small, Frequent Meals in the Clinical Care of Adults: A Review of the Evidence and Important Considerations. Nutrition in Clinical Practice. 2017;32(3):365–77. doi: 10.1177/0884533616662995 27589258

[6] Sylvetsky AC, Edelstein SL, Walford G, Boyko EJ, Horton ES, Ibebuogu UN, et al. A High-Carbohydrate, High-Fiber, Low-Fat Diet Results in Weight Loss among Adults at High Risk of Type 2 Diabetes. The Journal of nutrition. 2017;147(11):2060–6. Epub 2017/09/27. doi: 10.3945/jn.117.252395 . 28954840 PMC5657137

[7] Noakes TD, Windt J. Evidence that supports the prescription of low-carbohydrate high-fat diets: a narrative review. British Journal of Sports Medicine. 2017;51(2):133. doi: 10.1136/bjsports-2016-096491 28053201

[8] Scholtens EL, Krebs JD, Corley BT, Hall RM. Intermittent fasting 5:2 diet: What is the macronutrient and micronutrient intake and composition? Clinical Nutrition. 2020;39(11):3354–60. doi: 10.1016/j.clnu.2020.02.022 32199696

[9] Jaworski M, Panczyk M, Cedro M, Kucharska A. Adherence to dietary recommendations in diabetes mellitus: disease acceptance as a potential mediator. Patient Prefer Adherence. 2018;12:163–74. doi: 10.2147/PPA.S147233 . 29416318 PMC5790092

[10] Gibson AA, Sainsbury A. Strategies to Improve Adherence to Dietary Weight Loss Interventions in Research and Real-World Settings. Behav Sci (Basel). 2017;7(3):44. doi: 10.3390/bs7030044 . 28696389 PMC5618052

[11] García-Pérez L-E, Alvarez M, Dilla T, Gil-Guillén V, Orozco-Beltrán D. Adherence to therapies in patients with type 2 diabetes. Diabetes Ther. 2013;4(2):175–94. Epub 2013/08/30. doi: 10.1007/s13300-013-0034-y . 23990497 PMC3889324

[12] Shamanna P, Saboo B, Damodharan S, Mohammed J, Mohamed M, Poon T, et al. Reducing HbA1c in Type 2 Diabetes Using Digital Twin Technology-Enabled Precision Nutrition: A Retrospective Analysis. Diabetes Therapy. 2020;11(11):2703–14. doi: 10.1007/s13300-020-00931-w 32975712 PMC7547935

[13] Corral-Acero J, Margara F, Marciniak M, Rodero C, Loncaric F, Feng Y, et al. The ’Digital Twin’ to enable the vision of precision cardiology. European heart journal. 2020;41(48):4556–64. doi: 10.1093/eurheartj/ehaa159 . 32128588 PMC7774470

[14] Golse N, Joly F, Combari P, Lewin M, Nicolas Q, Audebert C, et al. Predicting the risk of post-hepatectomy portal hypertension using a digital twin: A clinical proof of concept. Journal of Hepatology. 2021;74(3):661–9. doi: 10.1016/j.jhep.2020.10.036 33212089

[15] Shao G, Helu M. Framework for a Digital Twin in Manufacturing: Scope and Requirements. Manuf Lett. 2020;24:10.1016/j.mfglet.2020.04.004. doi: 10.1016/j.mfglet.2020.04.004 . 32832379 PMC7431924

[16] Nyman E, Rozendaal YJ, Helmlinger G, Hamren B, Kjellsson MC, Stralfors P, et al. Requirements for multi-level systems pharmacology models to reach end-usage: the case of type 2 diabetes. Interface Focus. 2016;6(2):20150075. Epub 2016/04/07. doi: 10.1098/rsfs.2015.0075 ; PubMed Central PMCID: PMC4759745. 27051506 PMC4759745

[17] Bergman RN, Phillips LS, Cobelli C. Physiologic evaluation of factors controlling glucose tolerance in man: measurement of insulin sensitivity and beta-cell glucose sensitivity from the response to intravenous glucose. The Journal of clinical investigation. 1981;68(6):1456–67. doi: 10.1172/jci110398 . 7033284 PMC370948

[18] Cobelli C, Pacini G, Toffolo G, Sacca L. Estimation of insulin sensitivity and glucose clearance from minimal model: new insights from labeled IVGTT. American Journal of Physiology-Endocrinology and Metabolism. 1986;250(5):E591–E8. doi: 10.1152/ajpendo.1986.250.5.E591 3518490

[19] Cobelli C, Thomaseth K. The minimal model of glucose disappearance: optimal input studies. Mathematical Biosciences. 1987;83(2):127–55. 10.1016/0025-5564(87)90107-6.

[20] Bergman RN, Ider YZ, Bowden CR, Cobelli C. Quantitative estimation of insulin sensitivity. American Journal of Physiology-Endocrinology and Metabolism. 1979;236(6):E667. doi: 10.1152/ajpendo.1979.236.6.E667 443421

[21] Dalla Man C, Rizza RA, Cobelli C. Meal simulation model of the glucose-insulin system. IEEE Trans Biomed Eng. 2007;54(10):1740–9. Epub 2007/10/12. doi: 10.1109/TBME.2007.893506 . 17926672

[22] Kovatchev BP, Breton M, Man CD, Cobelli C. In silico preclinical trials: a proof of concept in closed-loop control of type 1 diabetes. J Diabetes Sci Technol. 2009;3(1):44–55. doi: 10.1177/193229680900300106 . 19444330 PMC2681269

[23] Herrgårdh T, Li H, Nyman E, Cedersund G. An Updated Organ-Based Multi-Level Model for Glucose Homeostasis: Organ Distributions, Timing, and Impact of Blood Flow. Front Physiol. 2021;12:619254–. doi: 10.3389/fphys.2021.619254 . 34140893 PMC8204084

[24] Sips FLP, Nyman E, Adiels M, Hilbers PAJ, Strålfors P, van Riel NAW, et al. Model-Based Quantification of the Systemic Interplay between Glucose and Fatty Acids in the Postprandial State. PLOS ONE. 2015;10(9):e0135665. doi: 10.1371/journal.pone.0135665 26356502 PMC4565650

[25] Lerche S, Soendergaard L, Rungby J, Moeller N, Holst JJ, Schmitz OE, et al. No increased risk of hypoglycaemic episodes during 48 h of subcutaneous glucagon-like-peptide-1 administration in fasting healthy subjects. Clinical Endocrinology. 2009;71(4):500–6. doi: 10.1111/j.1365-2265.2008.03510.x 19094067

[26] Krssak M, Brehm A, Bernroider E, Anderwald C, Nowotny P, Man CD, et al. Alterations in Postprandial Hepatic Glycogen Metabolism in Type 2 Diabetes. Diabetes. 2004;53(12):3048. doi: 10.2337/diabetes.53.12.3048 15561933

[27] Magnusson I, Rothman DL, Katz LD, Shulman RG, Shulman GI. Increased rate of gluconeogenesis in type II diabetes mellitus. A 13C nuclear magnetic resonance study. The Journal of clinical investigation. 1992;90(4):1323–7. doi: 10.1172/JCI115997 . 1401068 PMC443176

[28] Cedersund G, Roll J. Systems biology: model based evaluation and comparison of potential explanations for given biological data. FEBS journal. 2009;276(4):903–22. doi: 10.1111/j.1742-4658.2008.06845.x 19215297

[29] Rothman Douglas L, Magnusson I, Katz Lee D, Shulman Robert G, Shulman Gerald I. Quantitation of Hepatic Glycogenolysis And Gluconeogenesis in Fasting Humans With 13C NMR. Science. 1991;254(5031):573–6. doi: 10.1126/science.1948033 1948033

[30] Taylor R, Magnusson I, Rothman DL, Cline GW, Caumo A, Cobelli C, et al. Direct assessment of liver glycogen storage by 13C nuclear magnetic resonance spectroscopy and regulation of glucose homeostasis after a mixed meal in normal subjects. The Journal of clinical investigation. 1996;97(1):126–32. doi: 10.1172/JCI118379 . 8550823 PMC507070

[31] Firth RG, Bell PM, Marsh HM, Hansen I, Rizza RA. Postprandial hyperglycemia in patients with noninsulin-dependent diabetes mellitus. Role of hepatic and extrahepatic tissues. The Journal of clinical investigation. 1986;77(5):1525–32. doi: 10.1172/JCI112467 . 3517067 PMC424555

[32] Gerich JE. Role of the kidney in normal glucose homeostasis and in the hyperglycaemia of diabetes mellitus: therapeutic implications. Diabetic medicine: a journal of the British Diabetic. Association. 2010;27(2):136–42. doi: 10.1111/j.1464-5491.2009.02894.x . 20546255 PMC4232006

[33] Najjar SM, Perdomo G. Hepatic Insulin Clearance: Mechanism and Physiology. Physiology (Bethesda). 2019;34(3):198–215. doi: 10.1152/physiol.00048.2018 . 30968756 PMC6734066

[34] Duckworth WC, Bennett RG, Hamel FG. Insulin Degradation: Progress and Potential*. Endocrine Reviews. 1998;19(5):608–24. doi: 10.1210/edrv.19.5.0349 9793760

[35] Mather A, Pollock C. Glucose handling by the kidney. Kidney International. 2011;79:S1–S6. doi: 10.1038/ki.2010.509 21358696

[36] Holmstrup ME, Owens CM, Fairchild TJ, Kanaley JA. Effect of meal frequency on glucose and insulin excursions over the course of a day. e-SPEN, the European e-Journal of Clinical Nutrition and Metabolism. 2010;5(6):e277–e80. doi: 10.1016/j.eclnm.2010.10.001

[37] Paoli A, Tinsley G, Bianco A, Moro T. The Influence of Meal Frequency and Timing on Health in Humans: The Role of Fasting. Nutrients. 2019;11(4):719. doi: 10.3390/nu11040719 . 30925707 PMC6520689

[38] Taylor MA, Garrow JS. Compared with nibbling, neither gorging nor a morning fast affect short-term energy balance in obese patients in a chamber calorimeter. International Journal of Obesity. 2001;25(4):519–28. doi: 10.1038/sj.ijo.0801572 11319656

[39] Ahmed SR, Bellamkonda S, Zilbermint M, Wang J, Kalyani RR. Effects of the low carbohydrate, high fat diet on glycemic control and body weight in patients with type 2 diabetes: experience from a community-based cohort. BMJ open diabetes research & care. 2020;8(1):e000980. doi: 10.1136/bmjdrc-2019-000980 . 32193200 PMC7103851

[40] Shin Y, Park S, Choue R. Comparison of time course changes in blood glucose, insulin and lipids between high carbohydrate and high fat meals in healthy young women. Nutr Res Pract. 2009;3(2):128–33. Epub 2009/06/30. doi: 10.4162/nrp.2009.3.2.128 . 20016713 PMC2788176

[41] Mardinoglu A, Wu H, Bjornson E, Zhang C, Hakkarainen A, Rasanen SM, et al. An Integrated Understanding of the Rapid Metabolic Benefits of a Carbohydrate-Restricted Diet on Hepatic Steatosis in Humans. Cell Metab. 2018;27(3):559–71 e5. Epub 2018/02/20. doi: 10.1016/j.cmet.2018.01.005 ; PubMed Central PMCID: PMC6706084. 29456073 PMC6706084

[42] Nyman E, Brannmark C, Palmer R, Brugard J, Nystrom FH, Stralfors P, et al. A hierarchical whole-body modeling approach elucidates the link between in Vitro insulin signaling and in Vivo glucose homeostasis. J Biol Chem. 2011;286(29):26028–41. Epub 2011/05/17. doi: 10.1074/jbc.M110.188987 ; PubMed Central PMCID: PMC3138269. 21572040 PMC3138269

[43] Maas AH, Rozendaal YJW, van Pul C, Hilbers PAJ, Cottaar WJ, Haak HR, et al. A physiology-based model describing heterogeneity in glucose metabolism: the core of the Eindhoven Diabetes Education Simulator (E-DES). J Diabetes Sci Technol. 2015;9(2):282–92. Epub 2014/12/18. doi: 10.1177/1932296814562607 . 25526760 PMC4604593

[44] Kurata H. Virtual metabolic human dynamic model for pathological analysis and therapy design for diabetes. iScience. 2021;24(2):102101–. doi: 10.1016/j.isci.2021.102101 . 33615200 PMC7878987

[45] König M, Bulik S, Holzhütter H-G. Quantifying the Contribution of the Liver to Glucose Homeostasis: A Detailed Kinetic Model of Human Hepatic Glucose Metabolism. PLOS Computational Biology. 2012;8(6):e1002577. doi: 10.1371/journal.pcbi.1002577 22761565 PMC3383054

[46] Berndt N, Bulik S, Wallach I, Wunsch T, Konig M, Stockmann M, et al. HEPATOKIN1 is a biochemistry-based model of liver metabolism for applications in medicine and pharmacology. Nature communications. 2018;9(1):2386. doi: 10.1038/s41467-018-04720-9 ; PubMed Central PMCID: PMC6008457. 29921957 PMC6008457

[47] Ashworth WB, Davies NA, Bogle IDL. A Computational Model of Hepatic Energy Metabolism: Understanding Zonated Damage and Steatosis in NAFLD. PLOS Computational Biology. 2016;12(9):e1005105. doi: 10.1371/journal.pcbi.1005105 27632189 PMC5025084

[48] Xu K, Morgan KT, Todd Gehris A, Elston TC, Gomez SM. A Whole-Body Model for Glycogen Regulation Reveals a Critical Role for Substrate Cycling in Maintaining Blood Glucose Homeostasis. PLOS Computational Biology. 2011;7(12):e1002272. doi: 10.1371/journal.pcbi.1002272 22163177 PMC3233304

[49] Hall KD. Predicting metabolic adaptation, body weight change, and energy intake in humans. American Journal of Physiology-Endocrinology and Metabolism. 2009;298(3):E449–E66. doi: 10.1152/ajpendo.00559.2009 19934407 PMC2838532

[50] Brannmark C, Nyman E, Fagerholm S, Bergenholm L, Ekstrand EM, Cedersund G, et al. Insulin signaling in type 2 diabetes: experimental and modeling analyses reveal mechanisms of insulin resistance in human adipocytes. J Biol Chem. 2013;288(14):9867–80. doi: 10.1074/jbc.M112.432062 ; PubMed Central PMCID: PMC3617287. 23400783 PMC3617287

[51] Nyman E, Rajan MR, Fagerholm S, Brännmark C, Cedersund G, Strålfors P. A single mechanism can explain network-wide insulin resistance in adipocytes from obese patients with type 2 diabetes. The Journal of biological chemistry. 2014;289(48):33215–30. Epub 2014/10/15. doi: 10.1074/jbc.M114.608927 . 25320095 PMC4246081

[52] Rajan MR, Nyman E, Kjølhede P, Cedersund G, Strålfors P. Systems-wide Experimental and Modeling Analysis of Insulin Signaling through Forkhead Box Protein O1 (FOXO1) in Human Adipocytes, Normally and in Type 2 Diabetes. Journal of Biological Chemistry. 2016;291(30):15806–19. doi: 10.1074/jbc.M116.715763 27226562 PMC4957062

[53] Schmidt H, Jirstrand M. Systems Biology Toolbox for MATLAB: a computational platform for research in systems biology. Bioinformatics. 2006;22(4):514–5. Epub 2005/12/01. doi: 10.1093/bioinformatics/bti799 . 16317076

[54] Cedersund G. Conclusions via unique predictions obtained despite unidentifiability—new definitions and a general method. FEBS J. 2012;279(18):3513–27. Epub 2012/08/01. doi: 10.1111/j.1742-4658.2012.08725.x . 22846178

[55] Nadler SB, Hidalgo JU, Bloch T. Prediction of blood volume in normal human adults. Surgery. 1962;51(2):224–32. 21936146

[56] Eipel C, Abshagen K, Vollmar B. Regulation of hepatic blood flow: the hepatic arterial buffer response revisited. World journal of gastroenterology. 2010;16(48):6046–57. doi: 10.3748/wjg.v16.i48.6046 . 21182219 PMC3012579

