# Supplementary material for: Digital twin predicting diet response before and after long-term fasting
Source: PLoS Comput Biol. 2022 Sep 12;18(9):e1010469. doi: 10.1371/journal.pcbi.1010469 (PMC9499255; doi:10.1371/journal.pcbi.1010469)
Supplement: S1 Text — Fig A in S1 Text. Illustration of the complete model. Rectangles represent states, text with no squares are model reactions, and flows into Ø are flows leading out of model. Red colour represents the old model and blue the new. Dotted lines represent positive dependency regulations and dotted lines ending with a perpendicular head represent inhibitions. Table A in S1 Text. List of model states, its corresponding units, and a short description of what it represents. Initial value of simulations is represented as θ0. Table B in S1 Text. List of parameters and the starting guess, θ0, of each parameter in the parameter estimation. θ0_Healthy is the parameter starting guess for healthy populations and θ0_T2DM is for diabatic populations. The categorisation observed in Table B in S1 Text is based on the location of corresponding physiological mechanism, and parameters of flows between categories are specified by the category of the start of the flow, e.g. the parameter representing glucose diffusion between blood and tissue, k1, is specified in the blood category. Table C in S1 Text. Summary of clinical studies used to evaluate model. Table D in S1 Text. List of restriction bounds for optimisation. Θlow_Healthy is the lowest bound and Θhigh_Healthy is the highest bound when optimising parameters for the healthy population. Θlow_T2DM is the lowest bound and Θhigh_T2DM is the highest bound when optimising parameters for the diabetic population. Table E in S1 Text. Uncertainty of model parameters Θmin_Healthy is the lowest parameter value obtained in the fitting to healthy population and Θmax_Healthy is the highest. Θmin_T2DM is the lowest parameter value obtained in the fitting to diabetic population and Θmax_Healthy is the highest. (DOCX) [file pcbi.1010469.s001.docx]

# **Supplementary material**

Digital twin predicting diet response before and after long-term fasting

Oscar Silfvergren^a^, Christian Simonsson^a,b^, Mattias Ekstedt^b,c^, Peter Lundberg^b,d^, Peter Gennemark^a,e^, Gunnar Cedersund^a,b^

a) Department of Biomedical Engineering, IMT, Linköping University, Linköping, Sweden

b) Center for Medical Image Science and Visualization, Linköping University, Linköping, Sweden

c) Department of Health, Medicine, and Caring Sciences, Linköping University, Linköping, Sweden

d) Department of Medical Radiation Physics, and Department of Health, Medicine and Caring Sciences, Linköping University, Linköping, Sweden

e) Drug Metabolism and Pharma

d) Drug Metabolism and Pharmacokinetics, Research and Early Development, Cardiovascular, Renal and Metabolism (CVRM), BioPharmaceuticals R&D, AstraZeneca, Gothenburg, Sweden

Correspondence: Gunnar Cedersund, Email: gunnar.cedersund@liu.se, Department of Biomedical Engineering, Linköping University, 58185 Linköping, Sweden, +46-702-512323

**Table of Contents**

[**Introduction** 3](#_Toc104972988)

[**Model structure** 3](#_Toc104972989)

[**Model states** 4](#_Toc104972990)

[**Model parameters** 4](#_Toc104972991)

[**Model events** 9](#_Toc104972992)

[**Model description: ODEs and reactions** 9](#_Toc104972993)

[**Model variables** 21](#_Toc104972994)

[**Data** 22](#_Toc104972995)

[**Parameter fitting** 22](#_Toc104972996)

[**Model improvements** 26](#_Toc104972997)

[**References** 27](#_Toc104972998)

### **Introduction**

The proposed model extends a previously published short timescale model [1] and now serves on a multi-timescale with the ability to simulate metabolic flexibility during both short time periods, such as a meal, and slightly longer timer periods, up to a few days. This extension to longer timescales has required us to add a new organ to the model, the liver, and to describe some of its metabolic processes, such as glycogen synthesis and breakdown, protein synthesis and breakdown, digestion of meals containing protein, and long-term regulation of the corresponding fluxes. An overview of the old and the new model is given in Figure A, where the old model is color-coded red and new parts are color-coded blue.


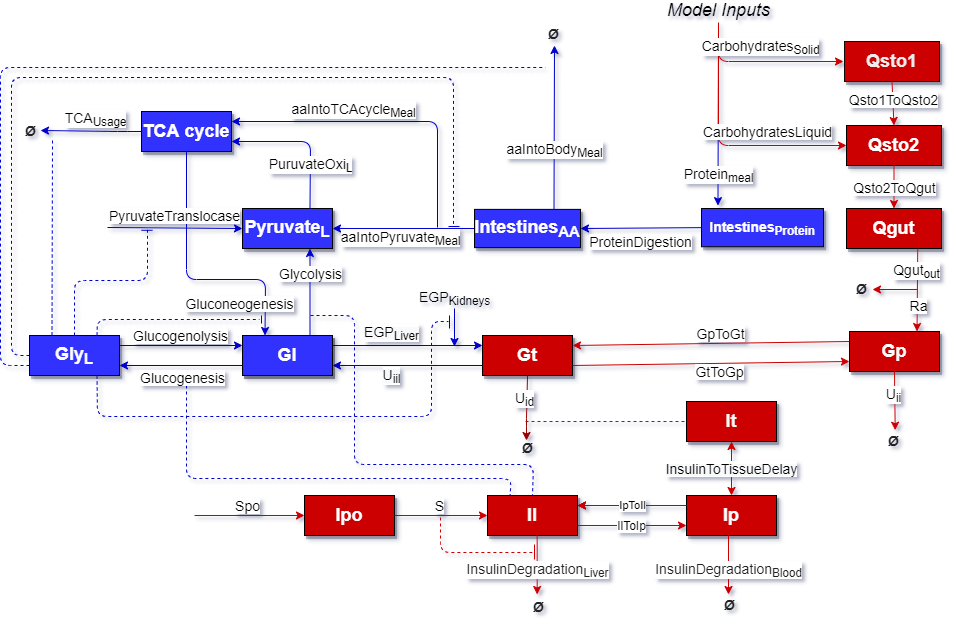


**Figure A, Illustration of the complete model.** Rectangles represent states, text with no squares are model reactions, and flows into Ø are flows leading out of model. Red colour represents the old model and blue the new. Dotted lines represent positive dependency regulations and dotted lines ending with a perpendicular head represent inhibitions.

### **Model structure**

The model consists of: i) states, ii) parameters, iii) events, and iv) the ordinary differential equations, and their constituent reactions. Each step is described separately below.

### **Model states**

The model states are dynamic quantities which were illustrated in Figure A as rectangles. The states are listed in Table A.

***Table A, List of model states, its corresponding units, and a short description of what it represents.*** *Initial value of simulations is represented as* **θ_0_***.*

| **Name** | **Unit** | **Description** | **θ_0_** |
| --- | --- | --- | --- |
| **Qsto1** | mg | Polysaccharides in stomach | 1e-50 |
| **Qsto2** | mg | Maltose/sugar/lactose in stomach | 0 |
| **Qgut** | mg | Carbohydrates in gut | 0 |
| **Intestine_Protein_** | mg/kg | Protein in intestines | 1e-50 |
| **Intestine_aa_** | mg/kg | Amino acids in intestines | 0 |
| **Gp** | mg/kg | Glucose in plasma | 80 |
| **Ip** | pmol/kg | Insulin in plasma | 1 |
| **Ipo** | pmol/kg | Insulin in portal vein | 0 |
| **Gt** | mg/kg | Glucose in tissue | 50 |
| **Gl** | mg/kg | Glucose in liver | 20 |
| **Gly_L_** | mg/kg | Hepatic glycogen | 1500 |
| **Pyruvate_L_** | mg/kg | Pyruvate in liver | 50 |
| **TCAcycle_L_** | mg/kg | Sum of TCA cycle components in liver | 50 |
| **Il** | pmol/kg | Insulin in liver | 1 |
| **InsulinStabilization** | pmol/kg | Part of insulin production calculations | 0 |
| **InsulinToTissueDelay** | pmol/L | Insulin response in tissue | 0 |

To ensure that the model is in a realistic steady state and in agreement with data at time point 0 a diet is simulated 5 days prior of every prediction. The initial values of simulation are thus individualized based on the wanted simulation start and specifically calibrated to each dataset. For this reason, the initial values of all states in a particular simulation at day 0 will generally deviate from the initial parameters of Table A.

### **Model parameters**

Three different types of parameters were used in the model: i) general parameters, which do not vary from individual to individual, or population to population, but which are different between diabetics and non-diabetics; ii) person-specific parameters, which are fitted specifically to on the specific population or individual; iii) model inputs, which are not optimized, but given by the experimental setting (when which meals are given, and what their content are) or by known demographics (sex, weight, height, diabetes status). The person-specific parameters are not parameters used in any model functions and only calibrates values of the general parameters (Table B) from a general metabolism, defined by all populations in the estimation data, to personified predictions. Please see section “Parameter fitting” for information regarding personification.

***Table B, List of parameters and the starting guess,* θ_0_, *of each parameter in the parameter estimation.***
**θ_0_Healthy_** *is the parameter starting guess for healthy populations and* **θ_0_T2DM_** *is for diabatic populations. The categorization observed in Table B is based on the location of corresponding physiological mechanism, and parameters of flows between categories are specified by the category of the start of the flow, e.g. the parameter representing glucose diffusion between blood and tissue, k_1_, is specified in the blood category.*

| **Category** | **Name** | **Unit** | **Description** | **θ_0_Healthy_** | **θ_0_T2DM_** |
| --- | --- | --- | --- | --- | --- |
| **General parameters** | **k_gri_** | 1/min | Breakdown of polysaccharides to monosaccharides | 0.06 | 0.05 |
|  | **k_min_** | 1/min | Minimum rate of gastric emptying | 0.01 | 0.01 |
|  | **k_max_** | 1/min | Maximum rate of gastric emptying | 0.12 | 0.1 |
|  | **b** | min | Time period of minimum rate of gastric emptying | 0.93 | 0.93 |
|  | **d** | min | Time period of maximum rate of gastric emptying | 0.01 | 0.03 |
|  | **k_abs_** | 1/min | Rate constant of flux from glucose in gut to glucose in blood | 0.03 | 0.02 |
|  | **ProteinBreakdown** | 1/min | Transportation rate of amino acids from intestines | 0.001 | 0.001 |
|  | **aaTransportion_K_** | 1/min | Breakdown rate of protein to amino acids in intestines | 0.01 | 0.02 |
|  | **f** | % | Percentage conversion of digested carbohydrate to glucose in blood | 0.66 | 0.96 |
|  | **k_1_** | 1/min | Glucose diffusion constant between blood and tissue | 0.06 | 0.04 |
|  | **U_ii_** | mg/kg/min | Insulin-independent glucose utilization from plasma | 0.54 | 0.73 |
|  | **m_2_** | 1/min | Insulin diffusion constant between insulin in blood and liver | 1.04 | 0.46 |
|  | **m_4_** | 1/min | Insulin degradation rate from blood | 0.07 | 0.06 |
|  | **K** | % | Pancreatic responsivity to the glucose rate of change and unit conversion | 491.26 | 407.43 |
|  | **S_b_** | pmol/kg/min | Basal insulin secretion | 0.01 | 0.01 |
|  | **gamma** | % | Pancreatic responsivity to glucose concentration | 447.91 | 299.76 |
|  | **K_m0_** | mg/kg | Rate of change of glucose utilization of muscle | 207.31 | 600 |
|  | **V_m0_** | mg/kg/min | Basal rate of glucose utilization of muscle | 3.51 | 1.1 |
|  | **V_mX_** | 1/min | Insulin dependent rate of glucose utilization of muscle | 0.12 | 0.56 |
|  | **K_f0_** | mg/kg | Rate of change of glucose utilization of adipocytyes | 290.24 | 469.5 |
|  | **V_f0_** | mg/kg/min | Basal rate of glucose utilization of adipocytes | 0.05 | 3 |
|  | **V_fX_** | 1/min | Insulin dependent rate of glucose utilization of adipocytes | 0.13 | 0.28 |
|  | **EGPLiver_diffusionMax_** | 1/min | Maximum transportation rate of glucose out of liver | 3.96 | 2.59 |
|  | **EGPLiver_diffusion0_** | mg/kg | Rate of change of glucose diffusion out of liver | 7.96 | 1.81 |
|  | **Uidl_diffusionMax_** | 1/min | Maximum transportation rate of glucose into the liver | 0.4 | 0.59 |
|  | **Uidl_diffusion0_** | mg/kg | Transportation rate of glucose into liver up to maximum rate | 0.91 | 1 |
|  | **EGP_KidneysK_** | 1/min | Non dynamic kidney glucose production | 0.62 | 0.94 |
|  | **k_2_** | 1/min | Diffusion constant of glucose between tissue and blood | 0.24 | 0.55 |
|  | **It_delayK_** | Dimensionless | Delay between rate of appearance of insulin in plasma and insulin response in tissue. | 0.35 | 1.5 |
|  | **G_b_** | mg/kg | Basal glucose concentration | 27.7 | 34.65 |
|  | **V_glyBmax_** | 1/min | Maximum rate of glycogenolysis | 4.73 | 6 |
|  | **GlyB** | mg/kg | Rate of glycogenolysis | 81.55 | 120 |
|  | **V_glySmax_** | 1/min | Maximum rate of glycogenesis | 4.74 | 1.72 |
|  | **GlyS** | mg/kg | Rate of glycogenesis | 0.8 | 0.4 |
|  | **TCAusage_K_** | 1/min | Rate of disappearance of amino acids in liver from the TCA cycle | 0.99 | 0.3 |
|  | **Gluconeogenesis_K_** | 1/min | Rate of appearance of pyruvate from the body into the liver | 1.82 | 2.5 |
|  | **PyruvateTranslocase_K_** | 1/min | Gluconeogenesis from the TCA cycle in the liver | 0.01 | 0 |
|  | **PyruvateOxi_K_** | 1/min | Oxidation of pyruvate to AcetylCoA in the liver | 0.03 | 0.05 |
|  | **Aminoprofile_K_** | % | Amino acid profile estimation from a meal | 0.76 | 0.34 |
|  | **Glycolysis_k_** | 1/min | Glycogen-independent rate constant of glycolysis | 0.1 | 0.46 |
|  | **Glycolysis_EXP_** | Dimensionless | Glycolysis dependency in relation to hepatic insulin response | 0.04 | 0 |
|  | **InsulinDep_EXP_** | Dimensionless | Exponential relation between usage of glucose and insulin | 1.8 | 1 |
|  | **m_1_** | 1/min | Insulin diffusion constant between insulin in the liver and glucose in plasma | 0.18 | 0.44 |
|  | **m_5_** | 1/min | Hepatic extraction of insulin dependent on the secretion of insulin between the portal vein and the liver | 0.13 | 0.08 |
|  | **m_6_** | 1/min | Basal hepatic extraction of insulin | 0.23 | 0.6 |
|  | **alpha** | Dimensionless | Delay between glucose signal and insulin secretion | 5.27 | 3.12 |
|  | **beta** | Dimensionless | Pancreatic response to hypoglycemia and hyperglycemia | 0.06 | 0.05 |
|  | **InsulinLiverResponseK** | Dimensionless | Hepatic insulin response | 0.11 | 0.87 |
|  | **GlyDep_Meal_** | mg/kg | Amino acid transportation dependency on glycogen as an energy regulator | 464.13 | 414.29 |
|  | **GlyDep_TCA_** | mg/kg | Glucose utilization dependent on glycogen as an energy regulator | 254.49 | 347.02 |
|  | **GlyDep_Gluconeogenesis_** | mg/kg | Rate of gluconeogenesis dependent on glycogen as an energy regulator | 943.61 | 750 |
|  | **GlyDepInFlow_K_** | mg/kg | Flow into the model dependent on glycogen as an energy regulator | 44.17 | 50.02 |
|  | **GlyDepEXP_Meal_** | Dimensionless | Amount of protein used for gluconeogenesis dependent on glycogen as an energy regulator | 1.02 | 0.65 |
|  | **GlyDepEXP_TCA_** | Dimensionless | Glucose utilization dependent on glycogen as an energy regulator | 0.87 | 0.22 |
|  | **GlyDepEXP_Gluconeogenesis_** | Dimensionless | Rate of gluconeogenesis dependent on glycogen as an energy regulator | 1.24 | 0.9 |
|  | **GlyDepIn_kEXP_** | Dimensionless | Flow into the model dependent on glycogen as an energy regulator | 0.05 | 0.15 |
| **Model inputs** | **Meal_Length_** | min | Length of meal |  |  |
|  | **Meal_Start_** | min | Start time of meal |  |  |
|  | **Carbohydrate_Amount_** | g | Total amount of carbohydrates in meal |  |  |
|  | **Protein_Amount_** | g | Total amount of protein in meal |  |  |
|  | **MealSolid_Boolean_** | True or False | Boolean to determine if meal is in solid phase |  |  |
|  | **MealLiquid_Boolean_** | True or False | Boolean to determine if meal is in liquid phase |  |  |
|  | **Female_Boolean_** | True or False | Boolean to determine gender in estimation of blood volume |  |  |
|  | **Male_Boolean_** | True or False | Boolean to determine gender in estimation of blood volume |  |  |
|  | **Height** | cm | Height of the person, used to estimate blood volume |  |  |
|  | **BW_start_** | kg | Body weight of the person |  |  |
|  | **BloodVolumeUncertainty** | % | Uncertainty in estimating total blood volume |  |  |
|  | **BloodLiverUncerteinty** | % | Uncertainty in estimating blood volume in liver |  |  |

### **Model events**

A model stimulation, such as a meal, is done through the use of model events where the event changes values of parameters at specific times. The model events are boolean functions which start is defined when the simulations $\mathbf{Time}$ vector is equal to the model input **Meal_Start_** and lasts until the vector $\mathbf{Time}$ is equal to $\mathbf{Mea}\mathbf{l}_{\mathbf{End}}$. During the event, the model input $\mathbf{Mea}\mathbf{l}_{\mathbf{Boolean}}$ is temporarily changed from a 0 to a 1. The end of the event, $\mathbf{Mea}\mathbf{l}_{\mathbf{End}}$**,** is a sum of model input $\mathbf{Mea}\mathbf{l}_{\mathbf{Length}}$ and $\mathbf{Mea}\mathbf{l}_{\mathbf{Start}}$, see **Eq 1.1**.

$\mathbf{Mea}\mathbf{l}_{\mathbf{End}}\left[ \mathbf{min} \right]\mathbf{= Mea}\mathbf{l}_{\mathbf{Start}}\left[ \mathbf{min} \right]\mathbf{+Mea}\mathbf{l}_{\mathbf{Length}}\mathbf{[min]}$ **Eq 1.1.**

The phase of the meal is defined by the booleans **MealSolid_Boolean_** and **MealSolid_Liquid_**. During a meal event, the parameter **D** is set to $\mathbf{Mea}\mathbf{l}_{\mathbf{Amount}}$, and is ultimately used to calculate the rate of the digestion of the meal in relation to its size. This meal digestion rate is further explained in **Eq 5.1.1**. The full equations for the meal events are described in **Eq 1.1.1 – Eq 1.1.3**.

$\mathbf{MealTurnOn=}\mathrm{if} \left( \mathbf{Time= Mea}\mathbf{l}_{\mathbf{Start}} \right) \mathrm{then}\mathbf{(Mea}\mathbf{l}_{\mathbf{Boolean}}\mathbf{= 1)}$ **Eq 1.1.1.**

$\mathbf{StomachActivation=}\mathrm{if} \left( \mathbf{Time= Mea}\mathbf{l}_{\mathbf{Start}} \right) \mathrm{then}\mathbf{(D= Mea}\mathbf{l}_{\mathbf{Amount}}\mathbf{)}$ **Eq 1.1.2.**

$\mathbf{MealTurnOff=}\mathrm{if} \left( \mathbf{Time= Mea}\mathbf{l}_{\mathbf{End}} \right) \mathrm{then}\mathbf{(Mea}\mathbf{l}_{\mathbf{Boolean}}\mathbf{= 0)}$ **Eq 1.1.3.**

**Meal_Amount_** in **Eq 1.1.2.** is the amount of carbohydrates in the meal. This is calculated through converting the model input $\mathbf{Carbohydrat}\mathbf{e}_{\mathbf{Amount}}$ from [g] to [mg], see **Eq 1.2**.

$\mathbf{Mea}\mathbf{l}_{\mathbf{Amount}} \left[ \mathbf{mg} \right]\mathbf{=}\mathbf{10}^{\mathbf{3}}\mathbf{*Carbohydrat}\mathbf{e}_{\mathbf{Amount}}\left[ \mathbf{g} \right]$ **Eq 1.2.**

During a meal event a flow of macronutrients is sent into the model, see **Eq 1.4.1 – Eq 1.4.3.**

$\mathbf{Carbohydrat}\mathbf{e}_{\mathbf{Solid}}\left[ \frac{\mathbf{mg}}{\mathbf{min}} \right]\mathbf{=}\frac{\mathbf{10}^{\mathbf{3}}\mathbf{*Carbohydrat}\mathbf{e}_{\mathbf{Amount}}\left[ \mathbf{g} \right]}{\mathbf{Mea}\mathbf{l}_{\mathbf{Length}}\mathbf{[min]}}\mathbf{*MealSoli}\mathbf{d}_{\mathbf{Boolean}}\mathbf{*Mea}\mathbf{l}_{\mathbf{Boolean}}$ **Eq 1.4.1.**

$\mathbf{Carbohydrat}\mathbf{e}_{\mathbf{Liquid}}\left[ \frac{\mathbf{mg}}{\mathbf{min}} \right]\mathbf{=}\frac{\mathbf{10}^{\mathbf{3}}\mathbf{*Carbohydrat}\mathbf{e}_{\mathbf{Amount}}\left[ \mathbf{g} \right]}{\mathbf{Mea}\mathbf{l}_{\mathbf{Length}}\mathbf{[min]}}\mathbf{*MealLiqui}\mathbf{d}_{\mathbf{Boolean}}\mathbf{*Mea}\mathbf{l}_{\mathbf{Boolean}}$ **Eq 1.4.2.**

$\mathbf{Protei}\mathbf{n}_{\mathbf{Meal}}\left[ \frac{\mathbf{mg}}{\mathbf{min}} \right]\mathbf{=}\frac{\mathbf{10}^{\mathbf{3}}\mathbf{* Protei}\mathbf{n}_{\mathbf{Amount}}\mathbf{[g]}}{\mathbf{Mea}\mathbf{l}_{\mathbf{Length}}\mathbf{[min]}}\mathbf{*Mea}\mathbf{l}_{\mathbf{boolean}}$ **Eq 1.4.3.**

where $\mathbf{Mea}\mathbf{l}_{\mathbf{boolean}}$ is the declared boolean that turns on and off the meal, where $\mathbf{MealSoli}\mathbf{d}_{\mathbf{Boolean}}$ and $\mathbf{MealLiqui}\mathbf{d}_{\mathbf{Boolean}}$ declares the phase of the meal, and where $\mathbf{Carbohydrat}\mathbf{e}_{\mathbf{Amount}}$ and $\mathbf{Protei}\mathbf{n}_{\mathbf{Amount}}$ are the total amount of respective nutrients in the meal. Both **Eq 1.4.1 – Eq 1.4.3** thus calculate a flow of respective macronutrients during the meal and assumes that the meal is consumed at a constant rate.

### **Model description: ODEs and reactions**

A model reaction corresponds to a chemical reaction or flow of material. The reactions, together with the inputs, events, and parameters, define how the model states change over time. Each ODE describes the time derivative of a specific state (Table A). All ODEs will henceforth be defined one by one, together with their underlying assumptions. Each ODE is assigned a unique identifier, and all auxiliary equations used in that ODE are assigned identifiers that extend the identifier of the corresponding ODE. For example, the third equations corresponding to ODE numbered X is numbered as X.3.

The first ODE describes the amount of protein in the intestines, and is a new equation not present in previous models. The state $\mathbf{Intestines}_{\mathbf{Protein}}$ describes the pool of protein from a meal in the intestines. The ODE of the state $\mathbf{Intestines}_{\mathbf{Protein}}$ is dependent on **Protein_Meal_** (**Eq 1.4.3**), and function $\mathbf{ProteinDigestion}$, which is the rate of digestion from protein into amino acids, see **Eq 2**.

$\frac{\mathbf{d}}{\mathbf{dt}}\left( \mathbf{Intestines}_{\mathbf{Protein}} \right)\left[ \frac{\mathbf{mg}}{\mathbf{kg}} \right]\mathbf{=}\left( \frac{\mathbf{Protei}\mathbf{n}_{\mathbf{Meal}}\left[ \frac{\mathbf{mg}}{\mathbf{min}} \right]}{\mathbf{BW}\left[ \mathbf{kg} \right]} \right)\mathbf{-}\left( \mathbf{ProteinDigestion}\left[ \frac{\mathbf{mg/kg}}{\mathbf{min}} \right] \right)$ **Eq 2.**

where the parameter $\mathbf{BW}$is the total bodyweight of the simulated person in kg. The function $\mathbf{ProteinDigestion}$ is the product of the parameter $\mathbf{ProteinBreakdown}$ and the amount of protein in the intestines, state $\mathbf{Intestines}_{\mathbf{Protein}}$, see **Eq 2.1**.

$\mathbf{ProteinDigestion}\left[ \frac{\mathbf{mg/kg}}{\mathbf{min}} \right]\mathbf{=ProteinBreakdown}\left[ \frac{\mathbf{1}}{\mathbf{min}} \right]\mathbf{*}\mathbf{Intestines}_{\mathbf{Protein}}\left[ \frac{\mathbf{mg}}{\mathbf{kg}} \right]$ **Eq 2.1.**

$\mathbf{ProteinDigestion}$ affects the state $\mathbf{Intestines}_{\mathbf{aa}}$ which is the amount of amino acids in the intestines from food intake. The amino acids in the intestines get transported out to the body through the function $\mathbf{aaTransportatio}\mathbf{n}_{\mathbf{Meal}}$, see **Eq 3**.

$\frac{\mathbf{d}}{\mathbf{dt}}\left( \mathbf{Intestines}_{\mathbf{aa}} \right)\left[ \frac{\mathbf{mg}}{\mathbf{kg}} \right]\mathbf{=ProteinDigestion -}\left( \mathbf{aaTransportatio}\mathbf{n}_{\mathbf{Meal}} \right)$ **Eq 3.**

The transportation of amino acids from intestines, $\mathbf{aaTransportatio}\mathbf{n}_{\mathbf{Meal}}$, is separated into two different flows;
i) $\mathbf{aaIntoLive}\mathbf{r}_{\mathbf{Meal}}$ and ii) $\mathbf{aaIntoBod}\mathbf{y}_{\mathbf{Meal}}$. The first of the flows, $\mathbf{aaIntoLive}\mathbf{r}_{\mathbf{Meal}}$**,** is the total flow of amino acids from meals into the liver. The second flow, $\mathbf{aaIntoBod}\mathbf{y}_{\mathbf{Meal}}$, represents the flow of amino acids from the meal that is not sent into the liver, see **Eq 3.1**.

$\mathbf{aaTransportatio}\mathbf{n}_{\mathbf{Meal}}\left[ \frac{\mathbf{mg/kg}}{\mathbf{min}} \right]\mathbf{= aaIntoLive}\mathbf{r}_{\mathbf{Meal}}\boldsymbol{+}\mathbf{aaIntoBod}\mathbf{y}_{\mathbf{Meal}}$ **Eq 3.1.**

The model flow $\mathbf{aaIntoBod}\mathbf{y}_{\mathbf{Meal}}$ is the product of the parameter $\mathbf{ProteinMea}\mathbf{l}_{\mathbf{K}}$, which is a general amino acid transportation constant, the total amount of amino acids in the intestines, state $\mathbf{Intestines}_{\mathbf{aa}}$, and the function $\mathbf{GlyDe}\mathbf{p}_{\mathbf{MealPositive}}$. $\mathbf{GlyDe}\mathbf{p}_{\mathbf{MealPositive}}$ is in a series of similarly structured functions that uses glycogen in the liver as a global homeostatic regulator. $\mathbf{GlyDe}\mathbf{p}_{\mathbf{MealPositive}}$upregulates the flow of amino acids out to the body when there is no shortage of stored energy, hence decreasing the relative flow of amino acids to the liver when gluconeogenesis is downregulated. This is further motivated by the increased use of amino acids for anabolic processes outside of the liver when the body is in an anabolic state, see **Eq 3.1.1.** for flow of amino acids to non-liver organs.

$\mathbf{aaIntoBod}\mathbf{y}_{\mathbf{Meal}} \left[ \frac{\mathbf{mg/kg}}{\mathbf{min}} \right]\mathbf{= ProteinMea}\mathbf{l}_{\mathbf{K}}\left[ \frac{\mathbf{1}}{\mathbf{min}} \right]\mathbf{*}\mathbf{Intestines}_{\mathbf{aa}}\left[ \frac{\mathbf{mg}}{\mathbf{kg}} \right]\mathbf{*GlyDe}\mathbf{p}_{\mathbf{MealPositive}}$ **Eq 3.1.1.**

The flow $\mathbf{aaIntoBod}\mathbf{y}_{\mathbf{Meal}}$ is upregulated during high energy levels through the function $\mathbf{GlyDe}\mathbf{p}_{\mathbf{MealPositive}}$. This function is defined by the quotient of the amount of hepatic glycogen, state $\mathbf{Gl}\mathbf{y}_{\mathbf{L}}$, and the parameter $\mathbf{GlyDe}\mathbf{p}_{\mathbf{Meal}}$. The dependency between the flow of amino acids and the energy levels in the liver does not necessarily need to be 1:1, and therefore the parameter $\mathbf{GlyDepEXP}_{\mathbf{Meal}}$ is introduced to ensure sufficient flexibility of the model, see **Eq 3.1.1.1.**

${\mathbf{GlyDe}\mathbf{p}_{\mathbf{Meal}\mathbf{Positive}}\mathbf{=}\left( \frac{\mathbf{Gl}\mathbf{y}_{\mathbf{L}}}{\mathbf{GlyDe}\mathbf{p}_{\mathbf{Meal}}} \right)}^{\mathbf{GlyDepEXP}_{\mathbf{Meal}}}$ **Eq 3.1.1.1.**

As described in **Eq** **3.1**, a fraction of the amino acids from ingested food is transported to the liver. The model assumes that these amino acids will either be metabolized to pyruvate or enter the TCA cycle. The amino acid profile of the meal determines which fraction enters each of the two processes and will either be sent into the liver as pyruvate, $\mathbf{aaInto}\mathbf{Pyruvate}_{\mathbf{Meal}}$, or into the TCA cycle, $\mathbf{aaInto}\mathbf{TCAcycle}_{\mathbf{Meal}}$, see **Eq 3.1.2.**

$\mathbf{aaIntoLive}\mathbf{r}_{\mathbf{Meal}}\left[ \frac{\mathbf{mg/kg}}{\mathbf{min}} \right]\mathbf{=}\mathbf{aaInto}\mathbf{Pyruvate}_{\mathbf{Meal}}\mathbf{+}\mathbf{aaInto}\mathbf{TCAcycle}_{\mathbf{Meal}}$ **Eq 3.1.2.**

The transportation rate of the amino acids into the liver is assumed to be at the same rate as to the rest of the body, hence both flows in **Eq** **3.1.2.** are dependent on the same parameter $\mathbf{ProteinMea}\mathbf{l}_{\mathbf{K}}$. The flow of amino acids from the intestines is also dependent on the total amount of amino acids in the intestines, state $\mathbf{Intestines}_{\mathbf{aa}}$. The separation of amino acids into the two different flows $\mathbf{aaInto}\mathbf{Pyruvate}_{\mathbf{Meal}}$ and $\mathbf{aaInto}\mathbf{TCAcycle}_{\mathbf{Meal}}$, is controlled by the parameter $\mathbf{Aminoprofil}\mathbf{e}_{\mathbf{K}}$, see **Eq 3.1.2.1** and **Eq 3.1.2.2.**

$\mathbf{aaInto}\mathbf{Pyruvate}_{\mathbf{Meal}} \left[ \frac{\mathbf{mg/kg}}{\mathbf{min}} \right]\mathbf{=Aminoprofil}\mathbf{e}_{\mathbf{K}} \left[ \boldsymbol{\%} \right]\mathbf{* ProteinMea}\mathbf{l}_{\mathbf{K}}\left[ \frac{\mathbf{1}}{\mathbf{min}} \right]$ $\mathbf{*}\mathbf{Intestines}_{\mathbf{aa}}\left[ \frac{\mathbf{mg}}{\mathbf{kg}} \right]\mathbf{*GlyDe}\mathbf{p}_{\mathbf{MealNegative}}$ **Eq 3.1.2.1.**

$\mathbf{aaInto}\mathbf{TCAcycle}_{\mathbf{Meal}}\left[ \frac{\mathbf{mg/kg}}{\mathbf{min}} \right]\mathbf{=}\left( \mathbf{1-Aminoprofil}\mathbf{e}_{\mathbf{K}} \right) \left[ \boldsymbol{\%} \right]\mathbf{*}\mathbf{ProteinMea}\mathbf{l}_{\mathbf{K}}\left[ \frac{\mathbf{1}}{\mathbf{min}} \right]$ $\mathbf{*}\mathbf{Intestines}_{\mathbf{aa}}\left[ \frac{\mathbf{mg}}{\mathbf{kg}} \right]\mathbf{*GlyDe}\mathbf{p}_{\mathbf{Meal}\mathbf{Negative}}$ **Eq 3.1.2.2.**

where the function $\mathbf{GlyDe}\mathbf{p}_{\mathbf{MealNegative}}$ makes the flow dependent on the fed state of the individual. This is motivated by the upregulation of gluconeogenesis during an unfed state, with an increased need for amino acids in the catabolic processes. The function $\mathbf{GlyDe}\mathbf{p}_{\mathbf{Meal}\mathbf{Negative}}$ thus introduces a negative dependency on energy levels, and when glycogen is high the flow becomes low. This is modelled using the previously defined parameter $\mathbf{GlyDep}_{\mathbf{Meal}}$ (in **Eq** **3.1.1.1**.) which is divided by the amount of glycogen in the liver, state $\mathbf{Gl}\mathbf{y}_{\mathbf{L}}$. However, this dependency does not necessarily need to be a ratio of 1:1, and the exponent $\mathbf{GlyDepEXP}_{\mathbf{Meal}}$ is introduced to ensure sufficient flexibility of the model. A high value of $\mathbf{GlyDepEXP}_{\mathbf{Meal}}$ makes the flow highly dependent on glycogen concentration and vice versa, see **Eq 3.1.2.3.**

$\mathbf{GlyDe}\mathbf{p}_{\mathbf{Meal}\mathbf{Negative}}\mathbf{=} \left( \frac{\mathbf{GlyDep}_{\mathbf{Meal}}}{\mathbf{Gl}\mathbf{y}_{\mathbf{L}}} \right)^{\mathbf{GlyDepEXP}_{\mathbf{Meal}}}$ **Eq 3.1.2.3.**

The next ODE describes the start of the carbohydrate metabolism from a solid meal in the stomach, state $\mathbf{Qsto1}$**,** and is kept intact from the Dalla Man model *et al*. (2007), see **Eq 4.**

$\frac{\mathbf{d}}{\mathbf{dt}}\left( \mathbf{Qsto1} \right)\left[ \mathbf{mg} \right]\mathbf{=}\left( \mathbf{Carbohydrat}\mathbf{e}_{\mathbf{Solid}} \right)\mathbf{-}\left( \mathbf{Qsto1toQsto2} \right)$ **Eq 4.**

The function $\mathbf{Qsto1ToQsto2}$describes the change of the state of ingested carbohydrates. As stated in the original Dalla Man model [1] there is a difference between ingested carbohydrates from food and from a liquid. The carbohydrates from a liquid meal goes directly to the next state, $\mathbf{Qsto2}$, and are hence not subjected to the time delay introduced by the ODE of $\mathbf{Qsto1}$. The time delay is calculated through multiplying the parameter $\mathbf{K}_{\mathbf{gri}}$, with the amount of carbohydrates in the stomach from ingested food. This step is therefore only necessary in the digestion of solid meals, flow $\mathbf{Carbohydrat}\mathbf{e}_{\mathbf{Solid}}$, and not liquids. The function $\mathbf{Qsto1ToQsto2}$represents the rate of change from this metabolic state and depends on the amount of carbohydrates in the state $\mathbf{Qsto1}$, see **Eq 4.1.**

$\mathbf{Qsto1ToQsto2}\left[ \frac{\mathbf{mg}}{\mathbf{min}} \right]\mathbf{=}\mathbf{K}_{\mathbf{gri}}\left[ \frac{\mathbf{1}}{\mathbf{min}} \right]\mathbf{*Qsto1}\left[ \mathbf{mg} \right]$ **Eq 4.1.**

The carbohydrates of the flow $\mathbf{Qsto1ToQsto2}$are further digested in the state $\mathbf{Qsto2}$. Carbohydrates that are not going through the extra digestion step is sent into the ODE of the state $\mathbf{Qsto2}$ through the flow **Carbohydate_Liquid_** which is declared in the event segment, see **Eq** **1.4.2.** $\mathbf{Qsto2ToQgut}$ is a function describing the flow of carbohydrates out of the state $\mathbf{Qsto2}$, see **Eq 5.**

$\frac{\mathbf{d}}{\mathbf{dt}}\left( \mathbf{Qsto2} \right)\left[ \mathbf{mg} \right]\mathbf{=}\left( \mathbf{Carbohydrat}\mathbf{e}_{\mathbf{Liquid}}\mathbf{+ Qsto1toQsto2} \right)\mathbf{-}\left( \mathbf{Qsto2ToQgut} \right)$ **Eq 5.**

The carbohydrates in the state $\mathbf{Qsto2}$ are digested and transported from the state with the function $\mathbf{Qsto2ToQgut}$, see **Eq 5.1.**

${\mathbf{Qsto2ToQgut}\left[ \frac{\mathbf{mg}}{\mathbf{min}} \right]\mathbf{=K}}_{\mathbf{empt}} \left[ \frac{\mathbf{1}}{\mathbf{min}} \right]\boldsymbol{*}\mathbf{Qsto2}\left[ \mathbf{mg} \right]$ **Eq 5.1.**

The flow of carbohydrates from the stomach, $\mathbf{Qsto2ToQgut}$, is partly depending on the function **K_empt_,** which is a kept intact from the previous model and describes the gastric emptying of the stomach into the gut, and the amount of carbohydrates in state $\mathbf{Qsto2}$. In the function **K_empt_**, the flow of carbohydrates is dependent on the size of the ingested meal, parameter $\mathbf{D}$, that is declared through an event at the start of the meal, and initially assigned the value of **Meal_Amount_**, which is the total size of carbohydrates in mg. The function value of **K_empt_** is dynamic and decreases over time defined by the function $\mathbf{aa}$, **Eq 5.1.2**, to the minimum rate of **K_Min_**, followed by an increase to **K_Max_** with the rate of the function **cc**, **Eq 5.1.3.** $\mathbf{Q}_{\mathbf{sto}}$is the sum of carbohydrates in the stomach, **Eq 5.1.4.**

The parameter $\mathbf{b}$ declares the decreasing rate of change at $\frac{\boldsymbol{K}_{\boldsymbol{max}}\boldsymbol{-}\boldsymbol{K}_{\boldsymbol{min}}}{\boldsymbol{2}}$ , and vice versa for the parameter $\mathbf{d}$ for the increasing rate of change to **K_Max_**, see **Eq 5.1.1.** for function **K_empt_**.

$\mathbf{K}_{\mathbf{empt}}\left[ \frac{\mathbf{1}}{\mathbf{min}} \right]\mathbf{=}\mathbf{K}_{\mathbf{min}}\mathbf{+}\frac{\mathbf{K}_{\mathbf{max}}\mathbf{-}\mathbf{K}_{\mathbf{min}}}{\mathbf{2}}\mathbf{*tanh}\left( \mathbf{aa*}\left( \mathbf{Q}_{\mathbf{sto}}\mathbf{-b*D} \right) \right)\mathbf{-tanh}\left( \mathbf{cc*}\left( \mathbf{Q}_{\mathbf{sto}}\mathbf{-d*D} \right) \right)\mathbf{+2)}$ **Eq 5.1.1.**

The transition between the two digestion rates, **K_Max_** and **K_Min_,** is calculated through the functions **aa** and **cc**. The transitions are dependent on the parameter $\mathbf{D}$, total size of the meal, and the parameters $\mathbf{b}$ and $\mathbf{d}$, which are the same parameters as in **Eq 5.1.1**, see **Eq 5.1.2** and **Eq 5.1.3.**

$\mathbf{aa}\boldsymbol{=}\frac{\mathbf{2.5*D}}{\mathbf{1-b}}$ **Eq 5.1.2.**

$\mathbf{cc}\boldsymbol{=}\frac{\mathbf{2.5*D}}{\mathbf{d}}$ **Eq 5.1.3.**

In the calculation of **K_empt_**, **Eq** **5.1.1**, a dependency on **Q_sto_** is declared. **Q_sto_** is simply the sum of the amount of carbohydrates in the stomach, states **Q_sto1_** and **Q_sto2_**, see **Eq 5.1.4.**

$\mathbf{Q}_{\mathbf{sto}}\left[ \mathbf{mg} \right]\mathbf{=}\mathbf{Q}_{\mathbf{sto1}}\left[ \mathbf{mg} \right]\mathbf{+}\mathbf{Q}_{\mathbf{sto2}}\left[ \mathbf{mg} \right]$ **Eq 5.1.4*.***

The flow of carbohydrates from the stomach to the gut, flow $\mathbf{Qsto2ToQgut}$, is sent into the state $\mathbf{Q}_{\mathbf{sto}}$, which represents the carbohydrates in the gut from a meal intake. The rate of the metabolism of carbohydrates to glucose is calculated through the function $\mathbf{Qgu}\mathbf{t}_{\mathbf{out}}$. The ODE of state $\mathbf{Qgut}$ is kept unchanged from previous model, see **Eq 6.**

$\frac{\mathbf{d}}{\mathbf{dt}}\left( \mathbf{Qgut} \right)\mathbf{[mg}\boldsymbol{]}\mathbf{=}\left( \mathbf{Qsto2ToQgut} \right)\mathbf{-}\left( \mathbf{Qgu}\mathbf{t}_{\mathbf{out}} \right)$ **Eq 6.**

Carbohydrates in state **Qgut** leaves the gut by the flow $\mathbf{Qgu}\mathbf{t}_{\mathbf{out}}$, defined as the product of the parameter **K_abs_** and the amount of carbohydrates in the gut, state $\mathbf{Qgut}$, see **Eq 6.1.**

$\mathbf{Qgu}\mathbf{t}_{\mathbf{out}}\left[ \frac{\mathbf{mg}}{\mathbf{min}} \right]\mathbf{=}\mathbf{K}_{\mathbf{abs}}\left[ \frac{\mathbf{1}}{\mathbf{min}} \right]\mathbf{*Qgut}\left[ \mathbf{mg} \right]$ **Eq 6.1.**

A substantial portion of the ingested carbohydrates are converted into glucose and transported into the plasma described as state $\mathbf{Gp}$. This rate of appearance of glucose from the meal is defined by the flow $\mathbf{Ra}$. From the state $\mathbf{Gp}$, the glucose uptake of tissue is represented by the flow $\mathbf{GpToGt}$. The insulin independent utilization of glucose, for example by the brain, is described by the parameter $\mathbf{U}_{\mathbf{ii}}$. Glucose uptake of plasma from tissue is defined by the flow $\mathbf{GtToGp}$**.** This ODE is kept intact from previous model, see **Eq 7.**

$\frac{\mathbf{d}}{\mathbf{dt}}\left( \mathbf{Gp} \right)\left[ \frac{\mathbf{mg}}{\mathbf{kg}} \right]\mathbf{=}\left( \mathbf{Ra+}\mathbf{GtToGp} \right)\mathbf{-}\left( \mathbf{U}_{\mathbf{ii}}\mathbf{+}\mathbf{GpToGt} \right)$ **Eq 7.**

In the function $\mathbf{Ra}$ the parameter$\mathbf{f}$ is introduced to account for losses from the ingested carbohydrates and the rate of appearance in plasma. The parameter **f** is defined as a percentage, and it is multiplied with the flow out of the state **Qgut** to get $\mathbf{Ra}$. In addition, the function **Ra** is divided by the total body weight, $\mathbf{BW}$to get the unit of $\mathbf{Gp}$, see **Eq 7.1.**

$\mathbf{Ra}\left[ \frac{\mathbf{mg/kg}}{\mathbf{min}} \right]\mathbf{=f}\left[ \boldsymbol{\%} \right]\mathbf{*}\frac{\mathbf{Qgu}\mathbf{t}_{\mathbf{out}}\left[ \frac{\mathbf{mg}}{\mathbf{min}} \right]}{\mathbf{BW [kg]}}$ **Eq 7.1.**

The flow of glucose from the plasma into tissue is calculated as the product of the parameter $\mathbf{K}_{\mathbf{1}}$ and the amount of glucose in plasma state $\mathbf{Gp}$, see **Eq 7.2.**

$\mathbf{GpToGt}\left[ \frac{\mathbf{mg/kg}}{\mathbf{min}} \right]\mathbf{=}\mathbf{K}_{\mathbf{1}}\left[ \frac{\mathbf{1}}{\mathbf{min}} \right]\mathbf{*Gp}\left[ \frac{\mathbf{mg}}{\mathbf{kg}} \right]$ **Eq 7.2.**

The glucose uptake from the tissue into the plasma is the product of the parameter $\mathbf{K}_{\mathbf{2}}$ and the total glucose in tissue, state $\mathbf{Gt}$, see **Eq 7.3.**

$\mathbf{GtToGp}\left[ \frac{\mathbf{mg/kg}}{\mathbf{min}} \right]\mathbf{=}\mathbf{K}_{\mathbf{2}}\left[ \frac{\mathbf{1}}{\mathbf{min}} \right]\mathbf{*Gt}\left[ \frac{\mathbf{mg}}{\mathbf{kg}} \right]$ **Eq 7.3.**

The insulin independent utilization of glucose is modelled by the parameter **U_ii_**, which represents the constant need of glucose from cells that cannot use other fuel sources, such as ketone bodies, even during longer periods of fasting.

All glucose in tissue is summarized as the state $\mathbf{Gt}$**.** Glucose is transported into tissue from plasma and by the endogenous glucose production, summarized as the flow $\mathbf{EGP}$. Compared to the previous model of $\mathbf{EGP}$, our model has been extended in the following ways: the physical interpretation has been changed from a non-physiological flow of glucose from an inexhaustible source into the model in form of a constant value minus the current external source of glucose, to a physical diffusion-driven flow directly influenced by glucose production from glycogenolysis and gluconeogenesis. $\mathbf{EGP}$is also expanded to include glucose production both from the liver and the kidneys and the total $\mathbf{EGP}$is the sum of glucose produced from both organs. An insulin-dependent utilization of glucose in tissue is represented by the function $\mathbf{U}_{\mathbf{id}}$. The transport of glucose into the liver is defined as $\mathbf{U}_{\mathbf{iil}}$, insulin independent utilization of liver, see the ODE of the state **Gt** in **Eq 8.**

$\frac{\mathbf{d}}{\mathbf{dt}}\left( \mathbf{Gt} \right)\left[ \frac{\mathbf{mg}}{\mathbf{kg}} \right]\mathbf{=}\left( \mathbf{GpToGt+EGP} \right)\mathbf{-}\left( \mathbf{U}_{\mathbf{id}}\mathbf{+}\mathbf{GtToGp+}\mathbf{U}_{\mathbf{iil}} \right)$ **Eq 8.**

The endogenous glucose production, function $\mathbf{EGP}$, is the sum of the production from the liver $\mathbf{EG}\mathbf{P}_{\mathbf{Liver}}$ and the kidneys $\mathbf{EG}\mathbf{P}_{\mathbf{Kidneys}}$, see **Eq 8.1.**

$\mathbf{EGP}\left[ \frac{\mathbf{mg/kg}}{\mathbf{min}} \right]\mathbf{=EG}\mathbf{P}_{\mathbf{Liver}}\boldsymbol{+}\mathbf{EG}\mathbf{P}_{\mathbf{Kidneys}}$ **Eq 8.1.**

The glucose production in the kidneys, function $\mathbf{EG}\mathbf{P}_{\mathbf{Kidneys}}$**,** is dependent on the fed state of the individual via the function $\mathbf{GlyDe}\mathbf{p}_{\mathbf{inflow}}$**.** This factor is multiplied by the parameter$\mathbf{EG}\mathbf{P}_{\mathbf{KidneysK}}$ to determine the magnitude of the gluconeogenesis in the kidneys, see **Eq 8.1.1.**

$\mathbf{EG}\mathbf{P}_{\mathbf{Kidneys}}\left[ \frac{\mathbf{mg/kg}}{\mathbf{min}} \right]\mathbf{=EG}\mathbf{P}_{\mathbf{KidneysK}}\left[ \frac{\mathbf{1}}{\mathbf{min}} \right]\boldsymbol{*}\mathbf{GlyDe}\mathbf{p}_{\mathbf{inflow}}\left[ \frac{\mathbf{mg}}{\mathbf{kg}} \right]$ **Eq 8.1.1.**

The gluconeogenesis is upregulated during time periods of low energy levels. This regulation is driven by the function $\mathbf{GlyDe}\mathbf{p}_{\mathbf{inflow}}$ defined by the quotient between the parameter $\mathbf{GlyDe}\mathbf{pIn}_{\mathbf{K}}$ and state $\mathbf{Gl}\mathbf{y}_{\mathbf{L}}$. As previously motivated for similar homeostatic regulations of hepatic glycogen, this dependency does not need to be a ratio of 1:1, hence the exponent $\mathbf{GlyDepI}\mathbf{n}_{\mathbf{kEGP}}$ is added to ensure model flexibility, see **Eq 8.1.1.1.**

$\mathbf{GlyDe}\mathbf{p}_{\mathbf{inflow}}\left[ \frac{\mathbf{mg/kg}}{\mathbf{min}} \right]\mathbf{=}\left( \frac{\mathbf{GlyDe}\mathbf{pIn}_{\mathbf{K}}}{\mathbf{Gl}\mathbf{y}_{\mathbf{L}}} \right)^{\mathbf{GlyDepI}\mathbf{n}_{\mathbf{kEGP}}}$ **Eq 8.1.1.1.**

In the equations of **EGP**, **Eq 8.1**, the glucose production from the liver is defined as $\mathbf{EG}\mathbf{P}_{\mathbf{Liver}}$. Glucose is transported into and out of the liver by the transport protein GLUT2. The amount of GLUT2 in the liver is limited, and the flow out of the liver, $\mathbf{EG}\mathbf{P}_{\mathbf{Liver}}$**,** and into the liver, **U_iil_**, can therefore be saturated. This saturation is modelled by the maximum flow parameters $\mathbf{EGPLive}\mathbf{r}_{\mathbf{MAX}}$ and $\mathbf{Uii}\mathbf{l}_{\mathbf{MAX}}$. The rate is defined by the parameter $\mathbf{EGPLive}\mathbf{r}_{\mathbf{0}}$. The diffusion rate of glucose from the liver into tissue is also dependent on the amount of glucose in the liver, state $\mathbf{Gl}$, see **Eq 8.1.2.**

$\mathbf{EG}\mathbf{P}_{\mathbf{Liver}}\left[ \frac{\mathbf{mg/kg}}{\mathbf{min}} \right]\mathbf{=}\frac{\mathbf{EGPLive}\mathbf{r}_{\mathbf{MAX}}\left[ \frac{\mathbf{1}}{\mathbf{min}} \right]\mathbf{* Gl}\left[ \frac{\mathbf{mg}}{\mathbf{kg}} \right]}{\mathbf{EGPLive}\mathbf{r}_{\mathbf{0}}\left[ \frac{\mathbf{mg}}{\mathbf{kg}} \right]\mathbf{+ Gl}\left[ \frac{\mathbf{mg}}{\mathbf{kg}} \right]}$ **Eq 8.1.2.**

The diffusion rate of glucose from tissue into liver is dependent on the amount of glucose in tissue, $\mathbf{Gt}$ and the same glucose transporter, GLUT2, as the transport of glucose out of the liver. To take into consideration that the state $\mathbf{Gt}$ is the sum of all glucose in tissue, different sets of parameters must be used to describe the two directions of GLUT2 transport. The maximum flow of glucose uptake of the liver is represented by the parameter $\mathbf{Uii}\mathbf{l}_{\mathbf{MAX}}$and the maximum rate defined by the parameter $\mathbf{Uii}\mathbf{l}_{\mathbf{0}}$, see **Eq 8.3.**

$\mathbf{U}_{\mathbf{iil}}\left[ \frac{\mathbf{mg/kg}}{\mathbf{min}} \right]\mathbf{=}\frac{\mathbf{Uii}\mathbf{l}_{\mathbf{MAX}}\left[ \frac{\mathbf{1}}{\mathbf{min}} \right]\mathbf{* Gt [}\frac{\mathbf{mg}}{\mathbf{kg}}\mathbf{]}}{\mathbf{Uii}\mathbf{l}_{\mathbf{0}}\left[ \frac{\mathbf{mg}}{\mathbf{kg}} \right]\mathbf{+ Gt [}\frac{\mathbf{mg}}{\mathbf{kg}}\mathbf{]}}$  **Eq 8.3.**

In the ODE describing glucose in tissue, state $\mathbf{Gt}$ (**Eq** **8.**), the insulin dependent utilization is defined by the function **U_id_**. This function is composed of a flow of glucose both into muscle tissue, $\mathbf{U}_{\mathbf{idm}}$, and into adipocytes, $\mathbf{U}_{\mathbf{idf}}$**,** see **Eq 8.3.1.**

$\mathbf{U}_{\mathbf{id}}\left[ \frac{\mathbf{mg/kg}}{\mathbf{min}} \right]\mathbf{=}\mathbf{U}_{\mathbf{idf}}\boldsymbol{+}\mathbf{U}_{\mathbf{idm}}$ **Eq 8.3.1.**

The maximum utilization of glucose in adipocytes is determined by the function $\mathbf{V}\mathbf{f}_{\mathbf{MAX}}$ and the maximum utilization of glucose in muscle is determined by the function $\mathbf{V}\mathbf{m}_{\mathbf{MAX}}$. Both utilization functions are dependent on the amount of glucose in tissue, state $\mathbf{Gt}$**,** where the half of the maximum velocity, $\mathbf{V}_{\mathbf{Max}}$, is reached when the value of the state **Gt** is the same value as $\mathbf{K}_{\mathbf{f0}}$ and $\mathbf{K}_{\mathbf{m0}}$ respectively**,** see **Eq 8.3.1.1.** and **8.3.1.2.**

$\mathbf{U}_{\mathbf{idf}}\left[ \frac{\mathbf{mg/kg}}{\mathbf{min}} \right]\mathbf{=}\frac{\mathbf{V}\mathbf{f}_{\mathbf{MAX}}\left[ \frac{\mathbf{1}}{\mathbf{min}} \right]\mathbf{*Gt}\left[ \frac{\mathbf{mg}}{\mathbf{kg}} \right]}{\mathbf{K}\mathbf{f}_{\mathbf{0}}\left[ \frac{\mathbf{mg}}{\mathbf{kg}} \right]\mathbf{+ Gt [}\frac{\mathbf{mg}}{\mathbf{kg}}\mathbf{]}}$ **Eq 8.3.1.1.**

$\mathbf{U}_{\mathbf{idm}}\left[ \frac{\mathbf{mg/kg}}{\mathbf{min}} \right]\mathbf{=}\frac{\mathbf{V}\mathbf{m}_{\mathbf{MAX}}\left[ \frac{\mathbf{1}}{\mathbf{min}} \right]\mathbf{*Gt}\left[ \frac{\mathbf{mg}}{\mathbf{kg}} \right]}{\mathbf{K}\mathbf{m}_{\mathbf{0}}\left[ \frac{\mathbf{mg}}{\mathbf{kg}} \right]\mathbf{+ Gt [}\frac{\mathbf{mg}}{\mathbf{kg}}\mathbf{]}}$ **Eq 8.3.1.2.**
The maximum reaction rates are given by the two functions **Vf_Max_** and **Vm_Max_**. These maximum flow functions are dependent on a basal value, $\mathbf{V}_{\mathbf{f0}}$ and $\mathbf{V}_{\mathbf{m0}}$**,** and a dynamic correlation to the insulin response in tissue, $\mathbf{InsulinResponseTissue}$. This dynamic correlation is calibrated by the parameters $\mathbf{V}_{\mathbf{fX}}$ and $\mathbf{V}_{\mathbf{mX}}$.

One limitation of previous models is that they do not scale well between small (<500 kcal) and big meals (>500 kcal). The reason for this may be that the model neglects glucose homeostatic systems that may play a bigger role during hyperglycaemia, for example renal exclusion, or that the glucose rate of appearance from the intestines is overestimated for big meals that may have a slower rate of appearance. Currently, we lack fundamental data to create models representing such hypotheses at a mechanistic level. To address the scalability problem with the least amount of non-physiological changes to the model, we introduce the parameter $\mathbf{InsulinDe}\mathbf{p}_{\mathbf{exp}}$ to increase the utilization of glucose during big meals by a nonlinear relation between usage of glucose in relation to insulin response in tissue. In future work we propose to solve the scalability problem either by expanding the carbohydrate metabolism, or introduce new systems, for example a dynamic glucose uptake by the brain and kidneys. The function $\mathbf{InsulinResponseTissue}$ represent the insulin response of the individual or population and may thus be decreased to represent individuals with high insulin resistance, thus enabling simulations of individuals with metabolic syndromes like type 2 diabetes, see **Eq** **8.3.1.3** and **Eq 8.3.1.4.**

$\mathbf{V}_{\mathbf{fmax}}\left[ \frac{\mathbf{1}}{\mathbf{min}} \right]\mathbf{=(}\mathbf{V}_{\mathbf{f0}}\mathbf{+}\mathbf{V}_{\mathbf{fX}}\mathbf{*InsulinResponseTissue}\mathbf{)}^{\mathbf{InsulinDe}\mathbf{p}_{\mathbf{exp}}}$ **Eq 8.3.1.3.**

$\mathbf{V}_{\mathbf{mmax}}\left[ \frac{\mathbf{1}}{\mathbf{min}} \right]\mathbf{=(}\mathbf{V}_{\mathbf{m0}}\mathbf{+}\mathbf{V}_{\mathbf{mX}}\mathbf{*InsulinResponseTissue}\mathbf{)}^{\mathbf{InsulinDe}\mathbf{p}_{\mathbf{exp}}}$ **Eq 8.3.1.4.**

A delay between the first appearance of insulin in the blood and the insulin response in tissue is kept unchanged from the previous model [1]. The rate of the delay is determined by the parameter $\mathbf{It}_{\mathbf{delayK}}$. A high value of $\mathbf{It}_{\mathbf{delayK}}$ results in a fast response, while a low value of the parameter $\mathbf{It}_{\mathbf{delayK}}$decreases the amplitude of the insulin in tissue response and lengthens it. The reaction $\mathbf{InsulinResponseTissue}$ is a delay of current insulin in the blood which is described with the state **Ip** [pmol/kg]. The state **Ip** is converted into the unit [pmol/L] through the model variable $\mathbf{Insuli}\mathbf{n}_{\mathbf{Blood}}$**,** declared in **Eq** **15.2,** see **Eq** **8.3.1.5.**

$\frac{\mathbf{d}}{\mathbf{dt}}\mathbf{(InsulinResponseTissue)=}\mathbf{It}_{\mathbf{delayK}}\mathbf{*}\left( \mathbf{Insuli}\mathbf{n}_{\mathbf{Blood}}\mathbf{-InsulinResponseTissue} \right)$ **Eq 8.3.1.5.**

As previously stated, the amount of glucose in tissue is closely linked to glucose in the liver through the flows of glucose into the liver, $\mathbf{U}_{\mathbf{iil}}$, and the flow of glucose from the liver, $\mathbf{EG}\mathbf{P}_{\mathbf{Liver}}$. The amount of glucose in the liver is represented by the state $\mathbf{Gl}$.

In the ODE of the state $\mathbf{Gl}$**,** synthesis of glycogen is represented as the model reaction $\mathbf{Glycogenesis}$ and the breakdown of glycogen into glucose by the model reaction $\mathbf{Glycogenolysis}$. Glucose may be converted into pyruvate through the model reaction $\mathbf{Glycolysis}$ and synthesized from the TCA cycle by the catabolic reaction $\mathbf{Gluconeogenesis}$, see **Eq 9.** for ODE of state $\mathbf{Gl}$.

$\frac{\mathbf{d}}{\mathbf{dt}}\left( \mathbf{Gl} \right)\left[ \frac{\mathbf{mg}}{\mathbf{kg}} \right]\mathbf{=}\left( \mathbf{U}_{\mathbf{iil}}\mathbf{+}\mathbf{Glycogenolysis+Gluconeogenesis} \right)\mathbf{-}\left( \mathbf{Glycogenesis}\mathbf{+ EG}\mathbf{P}_{\mathbf{Liver}}\mathbf{+}\mathbf{Glycolysis} \right)$ **Eq 9.**

Excess energy can be stored in the liver in the form of glycogen. This process, $\mathbf{Glycogenesis}$, is regulated through the anabolic hormone insulin. In the model, $\mathbf{Glycogenesis}$ is therefore dependent on the insulin response in the liver, represented by the function $\mathbf{InsulinResponse}_{\mathbf{L}}$.

The maximum reaction rate of $\mathbf{Glycogenesis}$ is set by the product of the parameter **Vglys_Max_** and the function $\mathbf{InsulinResponse}_{\mathbf{L}}$. The model reaction $\mathbf{Glycogenesis}$ also depends on the amount of glucose in the liver, state **Gl**, where the half of the maximum velocity, is reached when the value of the state $\mathbf{Gl}$ equals the value of the parameter $\mathbf{VglyS}_{\mathbf{0}}$**,** see **Eq** **9.1.**

$\mathbf{Glycogenesis}\left[ \frac{\mathbf{mg/kg}}{\mathbf{min}} \right]\mathbf{=}\frac{\mathbf{VglyS}_{\mathbf{max}}\left[ \frac{\mathbf{1}}{\mathbf{min}} \right]\mathbf{*}\mathbf{InsulinResponse}_{\mathbf{L}}\mathbf{* Gl [}\frac{\mathbf{mg}}{\mathbf{kg}}\mathbf{]}}{\mathbf{VglyS}_{\mathbf{0}}\left[ \frac{\mathbf{mg}}{\mathbf{kg}} \right]\mathbf{+Gl}\left[ \frac{\mathbf{mg}}{\mathbf{kg}} \right]}$  **Eq 9.1.**

$\mathbf{Glycogenesis}$ is regulated by insulin and depends on the function $\mathbf{InsulinResponse}_{\mathbf{L}}$. In the original Dalla man model, a state called $\mathbf{Il}$represents hepatic insulin and was used as an intermediate step between insulin in the portal vein and the blood plasma. In our model we have implemented this to have a physiological meaning with metabolic fluxes directly being dependent on the insulin in the liver. This insulin response therefore both affects the synthesis of glycogen and amino acids.

The function $\mathbf{InsulinResponse}_{\mathbf{L}}$represents the magnitude of the insulin response by the product of the insulin in the liver, the state $\mathbf{Il}$, and the parameter $\mathbf{InsulinLiverRespons}\mathbf{e}_{\mathbf{K}}$, see **Eq** **9.1.1.**

$\mathbf{InsulinResponse}_{\mathbf{L}}\boldsymbol{=}\mathbf{Il *InsulinLiverRespons}\mathbf{e}_{\mathbf{K}}$ **Eq 9.1.1.**

Glycogen is metabolized back into glucose through the model reaction $\mathbf{Glycogenolysis}$. The maximum rate of the reaction $\mathbf{Glycogenolysis}$ is described by the parameter **VglyB_Max_**. The model reaction $\mathbf{Glycogenolysis}$ is dependent on the amount of glycogen in the liver, state $\mathbf{Gl}\mathbf{y}_{\mathbf{L}}$**,** where half of the maximum velocity is reached when the value of the state $\mathbf{Gl}\mathbf{y}_{\mathbf{L}}$equals the parameter $\mathbf{VglyB}_{\mathbf{0}}$**,** see **Eq 9.2.**

$\mathbf{Glycogenolysis}\left[ \frac{\mathbf{mg/kg}}{\mathbf{min}} \right]\mathbf{=}\frac{\mathbf{VglyB}_{\mathbf{max}}\left[ \frac{\mathbf{1}}{\mathbf{min}} \right]\mathbf{*}\mathbf{Gl}\mathbf{y}_{\mathbf{L}} \left[ \frac{\mathbf{mg}}{\mathbf{kg}} \right]}{\mathbf{VglyB}_{\mathbf{0}}\left[ \frac{\mathbf{mg}}{\mathbf{kg}} \right]\mathbf{+}\mathbf{Gl}\mathbf{y}_{\mathbf{L}} \left[ \frac{\mathbf{mg}}{\mathbf{kg}} \right]}$ **Eq 9.2.**

With the two flows describing glycogen and glucose homeostasis, $\mathbf{Glycogenesis}$ and $\mathbf{Glycogenolysis}$, all equations describing the ODE of hepatic glycogen is described, see **Eq 9.2.1.**

$\frac{\mathbf{d}}{\mathbf{dt}}\left( \mathbf{Gl}\mathbf{y}_{\mathbf{L}} \right)\left[ \frac{\mathbf{mg}}{\mathbf{kg}} \right]\mathbf{=}\left( \mathbf{Glycogenesis} \right)\mathbf{-}\left( \mathbf{Glycogenolysis} \right)$ **Eq 9.2.1.**

Glucose in the liver (**Eq 9.**) is also converted into pyruvate through the anabolic process called $\mathbf{Glycolysis}$. This reaction is determined by the product of the parameter $\mathbf{Glycolysi}\mathbf{s}_{\mathbf{k}}$**,** the amount of glucose in liver, state $\mathbf{Gl}$, and the function $\mathbf{InsulinResponse}_{\mathbf{L}}$. The function $\mathbf{InsulinResponse}_{\mathbf{L}}$ makes the metabolic reaction $\mathbf{Glycolysis}$dependent on insulin. $\mathbf{InsulinResponse}_{\mathbf{L}}$ is the same function used to activate other anabolic reactions in the liver like $\mathbf{Glycogenesis}$. To allow the metabolic reactions to have different dependency on insulin, the reaction-specific exponent $\mathbf{GlycolysisEX}\mathbf{P}_{\mathbf{K}}$ is added. This is one of many parameters that can be set to calibrate the model to individuals with metabolic syndrome that alters the insulin responsiveness, see **Eq 9.3.**

$\mathbf{Glycolysis}\left[ \frac{\mathbf{mg/kg}}{\mathbf{min}} \right]\mathbf{= Glycolysi}\mathbf{s}_{\mathbf{k}}\left[ \frac{\mathbf{1}}{\mathbf{min}} \right]\mathbf{*Gl}\left[ \frac{\mathbf{mg}}{\mathbf{kg}} \right]\mathbf{*}\mathbf{InsulinResponse}_{\mathbf{L}}^{\mathbf{GlycolysisEX}\mathbf{P}_{\mathbf{K}}}$ **Eq 9.3.**

$\mathbf{InsulinResponse}_{\mathbf{L}}$ has previously been declared in **Eq 9.1.1.**

When glycogen stores are depleted the insulin-dependent utilization of glucose is lowered and the process of breaking down protein to glucose, $\mathbf{Gluconeogenesis}$, is upregulated. $\mathbf{Gluconeogenesis}$ represents the catabolic reaction of synthesizing glucose from amino acids in the TCA cycle. In the TCA cycle, the component oxaloacetate can be metabolized into phosphoenolpyruvic acid (PEP) and finally be metabolized into glucose. In the model, $\mathbf{Gluconeogenesis}$ in the liver is hence represented by a flow between a state describing the TCA cycle, state $\mathbf{TCAcycl}\mathbf{e}_{\mathbf{L}}$, into glucose in the liver, state $\mathbf{Gl}$, see **Eq 9.4.**

$\mathbf{Gluconeogenesis}\left[ \frac{\mathbf{mg/kg}}{\mathbf{min}} \right]\mathbf{= TCAcycl}\mathbf{e}_{\mathbf{L}}\left[ \frac{\mathbf{mg}}{\mathbf{kg}} \right]\mathbf{*Gluconeogenesi}\mathbf{s}_{\mathbf{TCAk}}\left[ \frac{\mathbf{1}}{\mathbf{min}} \right]\mathbf{*GlyDe}\mathbf{p}_{\mathbf{GluconeogenesisNegative}}$ **Eq 9.4.**

The model reaction $\mathbf{Gluconeogenesis}$depends on the previously declared global homeostatic regulator hepatic glycogen, state **GlyL**. This dependency is implemented through the multiplication with the function $\mathbf{GlyDe}\mathbf{p}_{\mathbf{GluconeogenesisNegative}}\mathbf{,}$defined in **Eq 9.4.1.**

$\mathbf{GlyDe}\mathbf{p}_{\mathbf{GluconeogenesisNegative}}\boldsymbol{=}\left( \frac{\mathbf{GlyDe}\mathbf{p}_{\mathbf{Gluconeogenesis}}}{\mathbf{Gl}\mathbf{y}_{\mathbf{L}}} \right)^{\mathbf{GlyDepEX}\mathbf{P}_{\mathbf{Gluconeogenesis}}}$ **Eq 9.4.1.**

The liver is responsible for a large portion of the synthesis of amino acids in the body. The process of converting glucose into pyruvate, $\mathbf{Glycolysis}$ (**Eq** **9.3**), is hence sent into the state $\mathbf{Pyruvat}\mathbf{e}_{\mathbf{L}}$, that represents the amount of pyruvate in the body. Pyruvate in the liver is produced either from **Glycolysis**, a flow from the body called $\mathbf{PyruvateTranslocase}$**,** or from a meal, flow $\mathbf{aaIntoPyruvat}\mathbf{e}_{\mathbf{Meal}}$. Pyruvate can be oxidized to Acetyl-CoA through the function $\mathbf{PyruvateOxi}_{\mathbf{L}}$ and be metabolized into glucose through the function $\mathbf{Gluconeogenesi}\mathbf{s}_{\mathbf{Pyruvate}}$, see **Eq** **10.** for ODE of the model state $\mathbf{Pyruvat}\mathbf{e}_{\mathbf{L}}$**.**

$\frac{\mathbf{d}}{\mathbf{dt}}\left( \mathbf{Pyruvat}\mathbf{e}_{\mathbf{L}} \right)\left[ \frac{\mathbf{mg}}{\mathbf{kg}} \right]\mathbf{=}\left( \mathbf{aaIntoPyruvat}\mathbf{e}_{\mathbf{Meal}}\mathbf{+Glycolysis+PyruvateTranslocase} \right)\mathbf{-}\left( \mathbf{PyruvateOxi}_{\mathbf{L}} \right)$ **Eq 10.**

The flow of pyruvate from the body into the liver is described through the function $\mathbf{PyruvateTranslocase}$**.** The function $\mathbf{PyruvateTranslocase}$ is the product of the parameter $\mathbf{PyruvateTranslocas}\mathbf{e}_{\mathbf{K}}$ and a dynamic relation to our global homeostatic regulator, function $\mathbf{GlyDe}\mathbf{p}_{\mathbf{inflow}}$. This relation to glycogen is set to the same relation as the other flow into the model used in the calculation of glucose from kidneys. This was done to reduce the amount of model parameters, so that the model does not get more complicated then needed. The previous declared function $\mathbf{GlyDe}\mathbf{p}_{\mathbf{inflow}}$(**Eq** **8.1.1.1**) is thus reused, see **Eq** **10.1.**

$\mathbf{PyruvateTranslocase}\left[ \frac{\mathbf{mg/kg}}{\mathbf{min}} \right]\mathbf{=PyruvateTranslocas}\mathbf{e}_{\mathbf{K}}\mathbf{*GlyDe}\mathbf{p}_{\mathbf{inflow}}$ **Eq 10.1.**

The oxidation of pyruvate into Acetyl-CoA, $\mathbf{PyruvateOxi}_{\mathbf{L}}$, is modelled by the product of the parameter $\mathbf{PyruvateOx}\mathbf{i}_{\mathbf{K}}$ and the total amount of pyruvate in the liver, state $\mathbf{Pyruvat}\mathbf{e}_{\mathbf{L}}$. This means that the oxidation is directly proportional to the amount of pyruvate in liver with the magnitude of the parameter $\mathbf{PyruvateOx}\mathbf{i}_{\mathbf{K}}$. This is a simplification of the true biology, but a more complex relation would be hard to justify without access to more data on pyruvate and Acetyl-CoA. The relationship of this oxidation on pyruvate into Acetyl-CoA is given in **Eq** **10.2.**

$\mathbf{PyruvateOx}\mathbf{i}_{\mathbf{L}}\left[ \frac{\mathbf{mg/kg}}{\mathbf{min}} \right]\mathbf{=Pyruvat}\mathbf{e}_{\mathbf{L}}\left[ \frac{\mathbf{1}}{\mathbf{min}} \right]\mathbf{* PyruvateOx}\mathbf{i}_{\mathbf{K}}\left[ \frac{\mathbf{mg}}{\mathbf{kg}} \right]$ **Eq 10.2.**

The model rection $\mathbf{PyruvateOx}\mathbf{i}_{\mathbf{L}}$ describes the oxidation of pyruvate into AcetylCoa. AcetylCoA is a component of the TCA cycle and to avoid unnecessary model complexity the TCA cycle is represented by a single state, $\mathbf{TCAcycl}\mathbf{e}_{\mathbf{L}}$. This simplification may impact the model behaviour where now all components of the cycle behave the same and amino acids that enter early in the cycle will either be utilized, function $\mathbf{TC}\mathbf{A}_{\mathbf{usage}}$, or metabolized into glucose, $\mathbf{Gluconeogenesis}$ (**Eq** **9.4.**), with the same rate and timing as amino acids enter the cycle in a later phase. We note that this model part must be expanded to represent alternative fuel sources such as ketones and fatty acids. However, for the use of simulating glucose/insulin/glycogen dynamics the simple representation is useful.

In the ODE of the state $\mathbf{TCAcycl}\mathbf{e}_{\mathbf{L}}$ components enter either from a meal, $\mathbf{aaIntoTCAcycl}\mathbf{e}_{\mathbf{Meal}}$, or from pyruvate$\mathbf{PyruvateOx}\mathbf{i}_{\mathbf{L}}$, see **Eq** **11.** for ODE of $\mathbf{TCAcycl}\mathbf{e}_{\mathbf{L}}$.

$\frac{\mathbf{d}}{\mathbf{dt}}\left( \mathbf{TCAcycl}\mathbf{e}_{\mathbf{L}} \right)\left[ \frac{\mathbf{mg}}{\mathbf{kg}} \right]\mathbf{=}\left( \mathbf{aaIntoTCAcycl}\mathbf{e}_{\mathbf{Meal}}\mathbf{+PyruvateOx}\mathbf{i}_{\mathbf{L}} \right)\mathbf{-}\left( \mathbf{Gluconeogenesi}\boldsymbol{s}\mathbf{+TC}\mathbf{A}_{\mathbf{usage}} \right)$ **Eq 11.**

The utilization of components of the TCA cycle is defined by the function $\mathbf{TC}\mathbf{A}_{\mathbf{usage}}$. The function $\mathbf{TC}\mathbf{A}_{\mathbf{usage}}$ is a product of the parameter $\mathbf{TCAusag}\mathbf{e}_{\mathbf{K}}$, the amount of components in the TCA cycle, state $\mathbf{TCAcycl}\mathbf{e}_{\mathbf{L}}$ and function $\mathbf{GlyDe}\mathbf{p}_{\mathbf{UtilizationPositive}}$, see **Eq** **11.1.**

$\mathbf{TC}\mathbf{A}_{\mathbf{usage}}\left[ \frac{\frac{\mathbf{mg}}{\mathbf{kg}}}{\mathbf{min}} \right]\mathbf{=TCAcycl}\mathbf{e}_{\mathbf{L}}\left[ \frac{\mathbf{mg}}{\mathbf{kg}} \right]\mathbf{*TCAusag}\mathbf{e}_{\mathbf{K}}\left[ \frac{\mathbf{1}}{\mathbf{min}} \right]\mathbf{* GlyDe}\mathbf{p}_{\mathbf{UtilizationPositive}}$ **Eq 11.1.**

The function $\mathbf{GlyDe}\mathbf{p}_{\mathbf{UtilizationPositive}}$ reduces wasted energy during catabolism and is constructed like all other functions regulated by the global energy homeostatic regulator glycogen. The physiological reason for this function is to preserve energy when glycogen stores in the liver are low. When this is the case, the body prioritize vital processes for survival, *i.e.* energy for the brain, that cannot use ketone bodies as fuel, and slowly deprioritizes the lesser important functions, such as muscle synthesis. To represent this dependency in the model, the hepatic glycogen, state $\mathbf{Gl}$, is divided by the parameter $\mathbf{GlyDe}\mathbf{p}_{\mathbf{Utilization}}$and the dependency to glycogen is calibrated through the parameter$\mathbf{GlyDepEXP}_{\mathbf{Utilization}}$, see **Eq 11.1.1.**

${\mathbf{GlyDe}\mathbf{p}_{\mathbf{UtilizationPositive}}\mathbf{=}\left( \frac{\mathbf{Gl}}{\mathbf{GlyDe}\mathbf{p}_{\mathbf{Utilization}}} \right)}^{\mathbf{GlyDepEXP}_{\mathbf{Utilization}}}$ **Eq 11.1.1.**

All other reactions in the ODE of state $\mathbf{TCAcycl}\mathbf{e}_{\mathbf{L}}$ (**Eq** **11**), has previously been defined; $\mathbf{aaIntoTCAcycl}\mathbf{e}_{\mathbf{Meal}}$ (**Eq** **3.1.2.2.**) and $\mathbf{PyruvateOx}\mathbf{i}_{\mathbf{L}}$ (**Eq 10.1.**).

When the body has excess glucose in the blood, hyperglycemia, the production of insulin in the pancreas is upregulated and is represented in model as function $\mathbf{Spo}$. From the pancreas the insulin is transported into the portal vein. Insulin concentration in the portal vein is represented by the state $\mathbf{Ipo}$. From the portal vein insulin is secreted through the function $\mathbf{S}$, see **Eq** **12** for the ODE of the state $\mathbf{Ipo}$.

$\frac{\mathbf{d}}{\mathbf{dt}}\left( \mathbf{Ipo} \right)\left[ \frac{\mathbf{pmol}}{\mathbf{kg}} \right]\mathbf{=Spo}\mathbf{-S}$ **Eq 12.**

The model function $\mathbf{S}_{\mathbf{po}}$ describes the rate of appearance of insulin in the portal vein. $\mathbf{S}_{\mathbf{po}}$ depends on three different parts: i) Rate of change of glucose in plasma, which is function $\mathbf{ChangeInGlucose}$ ii) glucose relation to a basal value which is $\mathbf{InsulinStabilization}$, and iii) a basal insulin production, which is parameter $\boldsymbol{S}_{\boldsymbol{b}}$**_,_** see **Eq** **12.1.**

$\mathbf{S}_{\mathbf{po}}\left[ \frac{\mathbf{pmol/kg}}{\mathbf{min}} \right]\mathbf{=ChangeInGlucose+}\mathbf{InsulinStabilization+S}_{\boldsymbol{b}}$ **Eq 12.1.**

The function $\mathbf{InsulinStabilization}$ represents production based on the difference between glucose in plasma, state $\mathbf{Gp}$, and the parameter $\mathbf{G}_{\mathbf{b}}$, which is the basal value of glucose between meals. The function $\mathbf{InsulinStabilization}$ is implemented as an ODE which causes a delay between an increase in glucose and the insulin production response. The size of the insulin response is determined by the parameter $\mathbf{beta}$, and the delay is determined by the parameter $\mathbf{alp}\mathbf{h}\mathbf{a}$. Lowering and increasing the parameter $\mathbf{beta}$ can henceforth be a part of calibrating insulin production response between individuals, enabling simulations of people with metabolic syndromes such as type 2 diabetes. The glucose in plasma is represented by the state $\mathbf{Gp}$ and because the unit of $\mathbf{Gp}$ is [mg/kg], the function $\mathbf{S}_{\mathbf{po}}$is divided by the molecular weight of glucose to change unit to [mmol/kg]. The parameter $\mathbf{beta}$ determines the amplitude of the response, and the unit conversion from ‘milli’ to ‘pico’ is implicitly part of the parameter value. The function $\mathbf{InsulinStabilization}$tries to balance the glucose level to the setpoint $\mathbf{G}_{\mathbf{b}}$between meals, see **Eq 12.1.1.**

$\frac{\mathbf{d}}{\mathbf{dt}}\mathbf{InsulinStabilization} \left[ \frac{\mathbf{pmol}}{\mathbf{kg}} \right]\mathbf{=}\frac{\mathbf{alp}\mathbf{h}\mathbf{a}}{\mathbf{180}\mathbf{.}\mathbf{16}}\mathbf{*}\left( \mathbf{beta}\mathbf{*}\left( \mathbf{Gp-}\mathbf{G}_{\mathbf{b}} \right)\mathbf{-InsulinStabilization} \right)$ **Eq 12.1.1.**

Balancing glucose is of high importance for the body and regulation of energy. Too high glucose levels increase the risk of haemoglobin, Hb, spontaneously binding to glucose creating HbA1C. HbA1C binds to glucose with high affinity and makes hemoglobin unable to transport oxygen over its lifetime. A high stabilization force, meaning a high value of the parameter $\mathbf{beta}$, results in an effective control of both insulin and glucose. Naturally this parameter is expected to be lower in a diabetic patient.

The second part of the $\mathbf{S}_{\mathbf{po}}$calculations is the function $\mathbf{ChangeInGlucose}$, which calculates the insulin production based on change of plasma glucose, the time derivative of the state $\mathbf{Gp}$. Model reaction $\mathbf{GpToGt}$(**Eq** **7.2.)**, and parameter $\mathbf{U}_{\mathbf{ii}}$, which is the insulin independent utilization of glucose, is the sum of all negative rates of change. Model flows $\mathbf{Ra}$ (**Eq** **7.1.**), and $\mathbf{GtToGp}$ (**Eq** **7.3.**) is the positive rate of changes of glucose. The amplitude of the insulin production response that is dependent to this previously mentioned rate of change is ultimately scaled by the parameter $\mathbf{K}$, which is divided by the molecular weight of glucose to change unit from [mg/kg] to [mmol/kg], see **Eq** **12.1.2.**

$\mathbf{Change in Glucose}\left[ \frac{\mathbf{mg/kg}}{\mathbf{min}} \right]\mathbf{=}\frac{\mathbf{K}}{\boldsymbol{180.16}}\boldsymbol{*}\left( \mathbf{Ra}\mathbf{+GtToGp-}\mathbf{U}_{\mathbf{ii}}\mathbf{-GpToGt} \right)$ **Eq 12.1.2.**

As previously mentioned, the third and final part of the insulin production is based on a basal insulin production that is determined by the parameter $\boldsymbol{S}_{\boldsymbol{b}}$.

The rate of disappearance of insulin from the portal vein, $\mathbf{S}$ (**Eq** **12.**), depends on the secretion of insulin from portal vein into the liver. This secretion is defined by the product of the parameter $\mathbf{gamma}$and the total insulin in the portal vein, state $\mathbf{Ipo}$, see **Eq** **12.2.**

$\mathbf{S}\left[ \frac{\mathbf{pmol/kg}}{\mathbf{min}} \right]\mathbf{=gamma}\left[ \frac{\mathbf{1}}{\mathbf{min}} \right]\mathbf{*Ipo}\left[ \frac{\mathbf{pmol}}{\mathbf{kg}} \right]$ **Eq 12.2.**

The flow **S** goes from the state $\mathbf{Ipo}$, insulin in portal vein, to the state $\mathbf{Il}$, insulin in liver. The ODE of $\mathbf{Il}$ was present in the previous model but did not affect any metabolic reactions in the model and simply worked as a delay between insulin production and the observed insulin concentration in circulation. The ODE of state $\mathbf{Il}$ is kept intact from the previous model, but the parameter values have been revised to reflect that the state $\mathbf{Il}$ now affects hepatic metabolic reactions such as synthesis of both glycogen and pyruvate. The flow $\mathbf{IpToIl}$describes the diffusion of insulin from the blood plasma to the liver and the flow $\mathbf{IlToIp}$ describes the flow back into the blood. $\mathbf{InsulinDegradation}_{\mathbf{Liver}}$ describes insulin degradation in the liver, see **Eq** **13.** for ODE of state $\mathbf{Il}$.

$\frac{\mathbf{d}}{\mathbf{dt}}\left( \mathbf{Il} \right)\left[ \frac{\mathbf{pmol}}{\mathbf{kg}} \right]\mathbf{=}\left( \mathbf{S+}\mathbf{IpToIl} \right)\mathbf{-}\left( \mathbf{IlToIp}\mathbf{+}\mathbf{InsulinDegradation}_{\mathbf{Liver}} \right)$ **Eq 13.**
The transport of insulin between the liver and the blood, $\mathbf{LiverToBloo}\mathbf{d}_{\mathbf{Insulin}}$, is defined by the product of the parameter $\mathbf{m}_{\mathbf{1}}$ and the total current amount of insulin in the liver, state $\mathbf{Il}$, see **Eq** **13.1.**

$$\mathbf{IpToIl}\left[ \frac{\mathbf{pmol/kg}}{\mathbf{min}} \right]\mathbf{=}\mathbf{m}_{\mathbf{1}}\left[ \frac{\mathbf{1}}{\mathbf{min}} \right]\mathbf{*Il}\left[ \frac{\mathbf{pmol}}{\mathbf{kg}} \right]$$

  **Eq 13.1.**

The transportation from the blood plasma to the liver is described by the flow $\mathbf{IlToIp}$. The function $\mathbf{IlToIp}$ is defined by the product of the parameter $\mathbf{m}_{\mathbf{2}}$and the amount of insulin in plasma, state $\mathbf{Ip}$, see **Eq** **13.2.**

$\mathbf{IlToIp}\left[ \frac{\mathbf{pmol/kg}}{\mathbf{min}} \right]\mathbf{=}\mathbf{m}_{\mathbf{2}}\left[ \frac{\mathbf{1}}{\mathbf{min}} \right]\mathbf{*Ip}\left[ \frac{\mathbf{pmol}}{\mathbf{kg}} \right]$ **Eq 13.2.**

The liver is the main organ for insulin degradation and approximately 50% of insulin from the portal vein never leaves the liver. This degradation is modelled by the function **InsulinDegradation_Liver_** defined by the product of the model function $\mathbf{M}_{\mathbf{3}}$and the insulin in the liver, state $\mathbf{Il}$. Function **InsulinDegradation_Liver_** and the subfunctions, $\mathbf{M}_{\mathbf{3}}$ and $\mathbf{HepaticExtraction}$are kept intact from the previous model, see **Eq** **13.3.**

$\mathbf{InsulinDegradation}_{\mathbf{Liver}}\left[ \frac{\mathbf{pmol/kg}}{\mathbf{min}} \right]\mathbf{=}\mathbf{M}_{\mathbf{3}}\left[ \frac{\mathbf{1}}{\mathbf{min}} \right]\mathbf{*Insuli}\mathbf{n}_{\mathbf{Liver}}\mathbf{[}\frac{\mathbf{pmol}}{\mathbf{kg}}\mathbf{]}$ **Eq 13.3.**

The insulin degradation in the liver, function $\mathbf{M}_{\mathbf{3}}$, depends on the parameter $\mathbf{m}_{\mathbf{1}}$and the function $\mathbf{HepaticExtraction}$, see **Eq** **13.3.1.**

$\mathbf{M}_{\mathbf{3}} \left[ \frac{\mathbf{1}}{\mathbf{min}} \right]\mathbf{=}\frac{\mathbf{HepaticExtraction} \mathbf{*}\mathbf{m}_{\mathbf{1}}}{\mathbf{1 -}\mathbf{HepaticExtraction}}$ **Eq 13.3.1.**

The model function $\mathbf{HepaticExtraction}$decreases the degradation during increased secretion of insulin into the liver. This results in a short delay between the increased rate of change of insulin into the liver and the insulin degradation. $\mathbf{HepaticExtraction}$is separated into a dependency on secretion of insulin, $\mathbf{S}$, and a basal value determined by the parameter $\mathbf{m}_{\mathbf{6}}$. The dependency on the secretion of insulin, **S**, is determined by the parameter $\mathbf{m}_{\mathbf{5}}$, see **Eq** **13.3.2.**

$\mathbf{HepaticExtraction} \mathbf{=-}\mathbf{m}_{\mathbf{5}}\mathbf{*S+}\mathbf{m}_{\mathbf{6}}$  **Eq 13.3.2.**

The transport of insulin from the liver into the blood plasma, $\mathbf{IlToIp}$ (**Eq 13.2**), is sent into the state $\mathbf{Ip}$which is the total amount of insulin in plasma. The ODE of state $\mathbf{Ip}$is kept intact from the previous model, see **Eq 14.** for ODE of state $\mathbf{Ip}$**.**

$\frac{\mathbf{d}}{\mathbf{dt}}\left( \mathbf{Ip} \right)\left[ \frac{\mathbf{pmol}}{\mathbf{kg}} \right]\mathbf{=}\left( \mathbf{IlToIp} \right)\mathbf{-}\left( \mathbf{IpToIl}\mathbf{+}\mathbf{InsulinDegradation}_{\mathbf{Blood}} \right)$ **Eq 14.**

Insulin degradation in plasma is calculated by the function $\mathbf{InsulinDegradation}_{\mathbf{Blood}}$, defined by product of the parameter $\mathbf{m}_{\mathbf{4}}$ and the amount of insulin in the blood, state $\mathbf{Ip}$, see **Eq 14.1.**

$\mathbf{InsulinDegradation}_{\mathbf{Blood}}\left[ \frac{\mathbf{pmol/kg}}{\mathbf{min}} \right]\mathbf{=}\mathbf{m}_{\mathbf{4}}\left[ \frac{\mathbf{1}}{\mathbf{min}} \right]\mathbf{*Ip}\left[ \frac{\mathbf{pmol}}{\mathbf{kg}} \right]$ **Eq 14.1.**

The final part of the model consists of model variables.

### **Model variables**

The glycogen concentration in the liver is often measured in the unit [mmol/L]. However, meals are often defined by the unit mg per kg bodyweight, and the previous model used the same unit for *e.g.* glycogen. To minimize unnecessary changes from the previous model and to keep most of the parameters with similar starting guesses, we have used the same unit here in our model as well. In future model development, one should consider changing the unit to mol or kg, without the bodyweight scaling. In the model, the variable $\mathbf{Glycogen}_{\mathbf{Liver}}$converts the unit from [mg/kg] to [mmol/L]. This is done through using the model input $\mathbf{BW}$, which is the total body weight, the molecular weight of glycogen, $\mathbf{MolecularWeight}_{\mathbf{Glycogen}}$**,** and the blood volume of the liver, $\mathbf{VolumeBloo}\mathbf{d}_{\mathbf{Liver}}$**,** see **Eq** **15.1.**

$\mathbf{Glycogen}_{\mathbf{Liver}} \left[ \frac{\mathbf{mmol}}{\mathbf{L}} \right]\mathbf{=}\frac{\mathbf{Gl}\mathbf{y}_{\mathbf{L}}\left[ \frac{\mathbf{mg}}{\mathbf{kg}} \right]\mathbf{* BW}\left[ \mathbf{kg} \right]}{\mathbf{VolumeBloo}\mathbf{d}_{\mathbf{Liver}} \left[ \mathbf{L} \right]\mathbf{*}\mathbf{MolecularWeight}_{\mathbf{Glycogen}}\left[ \frac{\mathbf{g}}{\mathbf{mol}} \right]}$ **Eq 15.1.**

The function $\mathbf{VolumeBloo}\mathbf{d}_{\mathbf{Liver}}$ estimates blood volume in the liver. This estimation assumes that the amount of blood in the liver is 13% of the total volume of blood in the body, calculated by function $\mathbf{VolumeBloodPeripheral}$. There are uncertainties both in the function estimating total blood volume, $\mathbf{VolumeBloodPeripheral}$, in data, and the assumption that the volume in the blood is 13% of the total blood volume. In several studies, the genders in populations are not homogenous or body weight and height are not documented, or both of those. To account for this uncertainty, the parameter **BloodLiver_Uncertainty_** is introduced to calibrate volume, see **Eq** **15.1.1.**

$\mathbf{VolumeBloodLiver}\left[ \mathbf{L} \right]\mathbf{=}$ $\mathbf{VolumeBloodPeripheral*0.13*}\mathbf{BloodLiver}_{\mathbf{uncertainty}}$ **Eq 15.1.1.**

Model variable $\mathbf{VolumeBloodLiver}$ is linked with the total blood volume in the body estimated by the model variable $\mathbf{VolumeBloodPeripheral}$. The function **VolumeBloodPeripheral** estimates the total amount of blood based on the model inputs $\mathbf{height}$, $\mathbf{BW}$ and the gender of the simulated person. The function **VolumeBlood_Peripheral_**, established by Samuel B. Nadler [2], is a well-documented linear function estimating total blood in a human based on information of gender, length, and bodyweight. **Boolean_Male_** and **Boolean_Female_** is true and false statements declared in model inputs in the beginning of the simulation. The blood estimation function is kept intact from Nadler *et al*. 1962 [2], see **Eq** **15.1.2.**

$\mathbf{VolumeBloo}\mathbf{d}_{\mathbf{Peripheral}} \left[ \mathbf{L} \right]\mathbf{=}$ $\mathbf{Boolean}_{\mathbf{male}}\left( \mathbf{0.3669*}\left( \frac{\mathbf{height}\left[ \mathbf{cm} \right]}{\mathbf{100}} \right)^{\mathbf{3}}\mathbf{+0.3219*BW}\left[ \mathbf{kg} \right]\mathbf{+0.6041} \right)\mathbf{*}\mathbf{BloodVolume}_{\mathbf{uncertainty}}\mathbf{+}\mathbf{Boolean}_{\mathbf{female}}\left( \mathbf{0.3561*}\left( \frac{\mathbf{height}\left[ \mathbf{cm} \right]}{\mathbf{100}} \right)^{\mathbf{3}}\mathbf{+0.3308*BW}\left[ \mathbf{kg} \right]\mathbf{+0.1833} \right)\mathbf{*}\mathbf{BloodVolume}_{\mathbf{uncertainty}}$ **Eq 15.1.2.**

The amount of insulin in the blood is often measured in the unit [pmol/L] and not the unit of the state representing insulin in blood plasma $\mathbf{Ip}$ [pmol/kg]. The unit of state $\mathbf{Ip}$ is unchanged from previous model. The model variable $\mathbf{Insuli}\mathbf{n}_{\mathbf{Blood}}$ is used to converts the unit from [pmol/kg] to [pmol/L], see **Eq** **15.2.**

$\mathbf{Insuli}\mathbf{n}_{\mathbf{Blood}}\mathbf{[}\frac{\mathbf{pmol}}{\mathbf{L}}\mathbf{]=}\frac{\mathbf{Ip}\left[ \frac{\mathbf{pmol}}{\mathbf{kg}} \right]\mathbf{* BW[kg]}}{\mathbf{VolumeBloodPeripheral}\left[ \mathbf{L} \right]}$ **Eq 15.2.**

The amount of insulin in the blood is often measured in the unit [mg/dL] and not the unit of the state representing glucose in blood plasma $\mathbf{Gp}$ [mg/kg]. Glucose in plasma is converted from [mg/kg] to [mg/dl] with the use of model variable $\mathbf{Glucos}\mathbf{e}_{\mathbf{Blood}}$, see **Eq 15.3.**

$\mathbf{Glucos}\mathbf{e}_{\mathbf{Blood}} \left[ \frac{\mathbf{mg}}{\mathbf{dL}} \right]\mathbf{=}\frac{\mathbf{Gp}\left[ \frac{\mathbf{mg}}{\mathbf{kg}} \right]\mathbf{* BW[kg]}}{\mathbf{VolumeBloodPeripheral}\left[ \mathbf{dL} \right]}$ **Eq 15.3.**

### **Data**

Data from a total of 7 existing clinical studies were used to evaluate model (Table C).

***Table C, Summary of clinical studies* used to evaluate model.**

| **Article** | **Glucose metabolic flexibility status** | **Type of study** | **Population** | **Age [years]** | **BMI [kg/m^2^]** | **Used for** |
| --- | --- | --- | --- | --- | --- | --- |
| Krssak *et al*. 2004 [4] | Healthy | Mixed meal | 5m/2f | 49 ± 2 | 25.8 ± 0.9 | Training |
|  | T2DM | Mixed meal | 5m/2f | 56 ± 3 | 26.9 ± 0.6 | Training |
| Rothman *et al*. 1991 [5] | Healthy | Fasting | N=7 |  |  | Validation |
| Magnusson *et al*. 1992 [6] | Healthy | Fasting | 4m/1f | 61 ± 5 | 25 ± 2 | Training |
|  | T2DM | Fasting | 5m/2f | 57 ± 4 | 28 ± 1 | Training |
| Firth *et al.* 1986 [7] | Healthy | OGTT | 1m/6f | 51±4 | 32.1 ± 2.0 | Validation |
| Lerche *et al.* 2009 [8] | Healthy | Fasting + OGTT | N=8​ | 24 ± 2 | 24.4 ± 0.7 | Training |
| Taylor *et al.* 1996 [3] | Healthy | Mixed meal | 6m/2f | mean 23.6 | mean 23.1 | Validation |
| Dalla Man *et al.* (2007) [1] | Healthy | OGTT | N=204 | 56 ± 2 | 78 ± 1 kg | Compare previous ‘Dalla Man model’ [1] with new model |

### **Parameter fitting**

Parameter values were set by a global optimization algorithm to fit model simulation to estimation data. The parameter values were restricted within a set interval from an expected value defined as between **Θ_low_** and **Θ_high_** value. Due to fundamental expected differences between healthy and diabatic population (T2DM) the bounds for corresponding optimization were set differently, see Table D.

***Table D, List of restriction bounds for optimization.***
***Θ_low_Healthy_*** *is the lowest bound and* ***Θ_high_Healthy_*** *is the highest bound when optimizing parameters for the healthy population.* ***Θ_low_T2DM_*** *is the lowest bound and* ***Θ_high_T2DM_*** *is the highest bound when optimizing parameters for the diabetic population.*

| **Name** | **Θ_low_Healthy_** | **Θ_high_Healthy_** | **Θ_low_T2DM_** | **Θ_high_T2DM_** |
| --- | --- | --- | --- | --- |
| **k_gri_** | 0.06 | 0.083 | 0.045 | 0.07 |
| **k_min_** | 0.008 | 0.01 | 0.007 | 0.01 |
| **k_max_** | 0.08 | 0.15 | 0.07 | 0.1 |
| **b** | 0.8 | 1 | 0.9 | 1 |
| **d** | 0.01 | 0.016 | 0.01 | 0.03 |
| **k_abs_** | 0.018 | 0.035 | 0.02 | 0.035 |
| **ProteinBreakdown** | 0.001 | 0.05 | 0.001 | 0.05 |
| **aaTransportion_K_** | 0.01 | 0.03 | 0.005 | 0.02 |
| **f** | 0.7 | 0.99 | 0.7 | 0.99 |
| **k_1_** | 0.05 | 0.07 | 0.04 | 0.07 |
| **U_ii_** | 0.5 | 1 | 0.5 | 1 |
| **m_2_** | 0.7 | 1 | 0.4 | 0.8 |
| **m_4_** | 0.047 | 0.075 | 0.06 | 0.2 |
| **K** | 400 | 510 | 400 | 500 |
| **S_b_** | 0.01 | 0.15 | 0.01 | 0.15 |
| **gamma** | 230 | 550 | 100 | 300 |
| **K_m0_** | 140 | 250 | 300 | 600 |
| **V_m0_** | 2 | 3.5 | 1.1 | 4 |
| **V_mX_** | 0.08 | 0.2 | 0.08 | 1 |
| **K_f0_** | 250 | 360 | 300 | 470 |
| **V_f0_** | 0.04 | 0.5 | 0.05 | 3 |
| **V_fX_** | 0.1 | 0.2 | 0.1 | 0.5 |
| **EGPLiver_diffusionMax_** | 2 | 6 | 1 | 6 |
| **EGPLiver_diffusion0_** | 1 | 15 | 1 | 15 |
| **Uidl_diffusionMax_** | 0.3 | 2 | 0.5 | 2 |
| **Uidl_diffusion0_** | 0.8 | 5 | 1 | 15 |
| **EGP_KidneysK_** | 0.3 | 1 | 0.3 | 1.3 |
| **k_2_** | 0.05 | 0.6 | 0.05 | 0.6 |
| **It_delayK_** | 0.1 | 1 | 0.02 | 1.5 |
| **G_b_** | 10 | 50 | 30 | 150 |
| **V_glyBmax_** | 1 | 5 | 1 | 6 |
| **GlyB** | 50 | 110 | 25 | 120 |
| **V_glySmax_** | 3 | 7 | 1 | 5 |
| **GlyS** | 0.4 | 1 | 0.4 | 1 |
| **TCAusage_K_** | 0.3 | 1.2 | 0.3 | 1.2 |
| **Gluconeogenesis_K_** | 1 | 2 | 1 | 2.5 |
| **PyruvateTranslocase_K_** | 0.005 | 0.02 | 0.005 | 0.02 |
| **PyruvateOxi_K_** | 0.022 | 1 | 0.05 | 3 |
| **Aminoprofile_K_** | 0.3 | 0.9 | 0.2 | 0.9 |
| **Glycolysis_k_** | 0.05 | 2 | 0.05 | 2 |
| **Glycolysis_EXP_** | 0.004 | 0.1 | 0.004 | 0.05 |
| **InsulinDep_EXP_** | 1 | 1.4 | 1 | 1.3 |
| **m_1_** | 0.1 | 0.3 | 0.1 | 1 |
| **m_5_** | 0.04 | 0.13 | 0.03 | 0.13 |
| **m_6_** | 0.15 | 0.5 | 0.35 | 0.7 |
| **alpha** | 2 | 8 | 2 | 5 |
| **beta** | 0.04 | 0.2 | 0.04 | 0.1 |
| **InsulinLiverResponseK** | 0.08 | 1.4 | 0.04 | 1.4 |
| **GlyDep_Meal_** | 320 | 500 | 200 | 450 |
| **GlyDep_TCA_** | 200 | 300 | 200 | 350 |
| **GlyDep_Gluconeogenesis_** | 650 | 1000 | 550 | 750 |
| **GlyDepInFlow_K_** | 30 | 80 | 50 | 100 |
| **GlyDepEXP_Meal_** | 1 | 1.4 | 0.6 | 1.2 |
| **GlyDepEXP_TCA_** | 0.65 | 1.2 | 0.2 | 0.5 |
| **GlyDepEXP_Gluconeogenesis_** | 0.7 | 1.61 | 0.3 | 0.9 |
| **GlyDepIn_kEXP_** | 0.03 | 0.1 | 0.02 | 0.15 |

Within the groups of “healthy” and “diabetic” there are still individual differences when it comes to insulin response, production, and clearance. To further evolve the model from predicting a general metabolism, defined as the mean metabolism based on the estimation data, to instead making predictions that are person or individual specific a model calibration has to be made.

The model may scale blood volumes based on the declared information (gender, weight, length). The total blood volume is estimated using equations published in Nadler et al. 1962 [2] (**Eq 15.1.2**) and the blood in the liver is assumed to be 13% of the total blood volume (**Eq 15.1.1**). Due to individual differences and fluctuations of blood during for example a meal responses, both blood volume estimations: i) total blood volume, and ii) liver blood volume, are assumed to be within a 30% uncertainty of the blood estimation equations ($\mathbf{BloodVolume}_{\mathbf{uncertainty}}$ and $\mathbf{BloodLiver}_{\mathbf{uncertainty}}$). Furthermore, in the declaration of anthropometry, the user also declares if the prediction should be of a diabetic or a healthy user, where the parameters used for the prediction will either be using the parameters derived from the diabetic population in the estimation data (Table B, **θ_0_Healthy_**), or of the parameters derived from the healthy population in the estimation data (Table B, **θ_0_T2DM_**).

When making personalized model predictions the initial values can change between individuals, and thus needs to be calibrated. The calibration of basal insulin is done by calibrating the parameter **S_b_**, calibration of basal glucose is calibrated through the parameter **G_b_**. Other initial values such as hepatic glycogen is not determined via changing a parameter, but is instead achieved through a steady state simulation, where the model is simulated for 5 days, using a calibration diet, prior of the main simulation start. This steady state simulation also ensures that the dynamics of the main simulation is not due to any transient behaviour caused by the model not being in equilibrium. The plausible calibration diet prior of the main simulation is declared exactly like all other meals, see **Eq 1.1.1 – 1.1.3**, where the bounds constrict the diet to be within 20-250 g of carbohydrates and 20-150 g of protein per meal.

Three model parameters are used to calibrate: insulin resistance, insulin clearance and insulin response, to account for both differences between individuals and populations. Firstly, parameters **K** and **beta** are calibrated with the same magnitude in order to personify insulin production. Secondly, parameters **m_5_** and **m_4_** are calibrated with the same magnitude in order to personify insulin clearance. Lastly, parameters **V_mx_** and **V_fx_** are calibrated with the same magnitude to personify differences in insulin response in the liver, muscle, and adipocytes. All populations within the group “healthy” and “diabetic” were assumed to be within 30% of the mean insulin response, clearance, and production (determined by the estimation to data) and thus the magnitude of the three parameters that scales all three were bound to be within the 30% interval.

***Table E, Variation of model parameters***
**Θ_min_Healthy_** *is the lowest parameter value in the fitting to healthy population and* ***Θ_max_Healthy_*** *is the highest.* ***Θ_min_T2DM_*** *is the lowest parameter value in the fitting to diabetic population and* ***Θ_max_Healthy_*** *is the highest.*

| **Name** | **Θ_min_Healthy_** | **Θ_max_Healthy_** | **Θ_min_T2DM_** | **Θ_max_T2DM_** |
| --- | --- | --- | --- | --- |
| **k_gri_** | 0.06 | 0.083 | 0.045 | 0.06 |
| **k_min_** | 0.008 | 0.009 | 0.007 | 0.007 |
| **k_max_** | 0.081 | 0.12 | 0.077 | 0.1 |
| **b** | 0.919 | 0.958 | 0.928 | 1 |
| **d** | 0.014 | 0.016 | 0.028 | 0.03 |
| **k_abs_** | 0.031 | 0.032 | 0.02 | 0.024 |
| **ProteinBreakdown** | 0.001 | 0.005 | 0.001 | 0.01 |
| **aaTransportion_K_** | 0.016 | 0.022 | 0.005 | 0.02 |
| **f** | 0.837 | 0.865 | 0.957 | 0.99 |
| **k_1_** | 0.062 | 0.063 | 0.04 | 0.04 |
| **U_ii_** | 0.669 | 0.69 | 0.701 | 0.873 |
| **m_2_** | 0.915 | 0.942 | 0.455 | 0.8 |
| **m_4_** | 0.066 | 0.072 | 0.06 | 0.114 |
| **K** | 461.221 | 504.261 | 407.151 | 449.984 |
| **S_b_** | 0.009 | 0.011 | 0.01 | 0.011 |
| **gamma** | 238.519 | 357.233 | 113.835 | 299.758 |
| **K_m0_** | 240.867 | 243.215 | 324.017 | 559.997 |
| **V_m0_** | 2.761 | 2.938 | 1.1 | 3.631 |
| **V_mX_** | 0.114 | 0.114 | 0.219 | 0.794 |
| **K_f0_** | 344.417 | 360 | 399.73 | 469.503 |
| **V_f0_** | 0.04 | 0.05 | 0.593 | 3 |
| **V_fX_** | 0.141 | 0.148 | 0.156 | 0.449 |
| **EGPLiver_diffusionMax_** | 3.299 | 3.6 | 1.907 | 2.601 |
| **EGPLiver_diffusion0_** | 6.701 | 7.849 | 1.74 | 1.905 |
| **Uidl_diffusionMax_** | 0.414 | 0.433 | 0.534 | 1.703 |
| **Uidl_diffusion0_** | 0.805 | 1.071 | 1 | 14.996 |
| **EGP_KidneysK_** | 0.64 | 0.684 | 0.938 | 1.299 |
| **k_2_** | 0.226 | 0.251 | 0.391 | 0.547 |
| **It_delayK_** | 0.291 | 0.387 | 0.043 | 1.5 |
| **G_b_** | 32.682 | 34.222 | 34.599 | 85.93 |
| **V_glyBmax_** | 3.963 | 4.105 | 4.383 | 6 |
| **GlyB** | 65.197 | 79.16 | 26.95 | 120 |
| **V_glySmax_** | 4.909 | 5 | 1.623 | 4.031 |
| **GlyS** | 0.781 | 0.857 | 0.4 | 0.829 |
| **TCAusage_K_** | 1.138 | 1.197 | 0.3 | 0.509 |
| **Gluconeogenesis_K_** | 1.367 | 1.57 | 1.334 | 2.5 |
| **PyruvateTranslocase_K_** | 0.01 | 0.011 | 0.005 | 0.011 |
| **PyruvateOxi_K_** | 0.04 | 0.043 | 0.05 | 2.993 |
| **Aminoprofile_K_** | 0.616 | 0.824 | 0.233 | 0.821 |
| **Glycolysis_k_** | 0.083 | 0.109 | 0.393 | 0.639 |
| **Glycolysis_EXP_** | 0.051 | 0.06 | 0.004 | 0.015 |
| **InsulinDep_EXP_** | 1.221 | 1.231 | 1 | 1.131 |
| **m_1_** | 0.216 | 0.22 | 0.383 | 1 |
| **m_5_** | 0.074 | 0.099 | 0.034 | 0.094 |
| **m_6_** | 0.255 | 0.288 | 0.395 | 0.603 |
| **alpha** | 4.05 | 4.373 | 2.618 | 3.205 |
| **beta** | 0.077 | 0.083 | 0.045 | 0.058 |
| **InsulinLiverResponseK** | 0.118 | 0.12 | 0.87 | 1.394 |
| **GlyDep_Meal_** | 434.097 | 466.701 | 200.029 | 415.016 |
| **GlyDep_TCA_** | 271.367 | 291.626 | 263.174 | 349.302 |
| **GlyDep_Gluconeogenesis_** | 647.722 | 861.522 | 553.885 | 750 |
| **GlyDepInFlow_K_** | 59.482 | 60.495 | 50 | 99.913 |
| **GlyDepEXP_Meal_** | 0.896 | 0.963 | 0.6 | 0.804 |
| **GlyDepEXP_TCA_** | 0.676 | 0.756 | 0.2 | 0.22 |
| **GlyDepEXP_Gluconeogenesis_** | 0.814 | 1.034 | 0.333 | 0.9 |
| **GlyDepIn_kEXP_** | 0.05 | 0.067 | 0.15 | 0.267 |

### **Model improvements**

The starting point for our model development has been the Herrgårdh *et al*. 2021 [9] model, which in turn is an update of the Nyman *et al*. 2011 [10] model, which was one of the first sub-division of the glucose uptake fluxes in the original Dalla Man *et al*. (2007) model into specific organ fluxes. More specifically, we have taken the updated distributions between the organ fluxes from Herrgårdh *et al.* (2021), but used the simplest version for the adipose tissue glucose uptake. In the full version of the Herrgårdh model, the adipose tissue glucose uptake consists of >20 ODEs, but since we are herein not considering details in the adipose tissue, we have replaced all of those with a single rate expression. This rate expression is the same as in the simplest adipose tissue sub-model in [10].

We have extended and improved the Herrgårdh model in different ways. Some of the additions that we have made to the model concerns *e.g.* the role of proteins and glycogen in glucose homeostasis at different time-scales. Another addition to the model is that we now, unlike in the previous Herrgårdh model, consider both postprandial (minutes to hours) and longer (<4 weeks) dynamic responses. These new multi-timescale capabilities of the model are all made possible by the most important addition to the model: intracellular details in the liver.

In the model, we introduce the main intracellular metabolic fluxes in the liver, which were not present in the previous models. In the original Dalla Man model, EGP was included as a separate phenomenological expression, but did not depend on the intracellular glucose concentration in the liver. In the later Herrgårdh model, the liver was introduced, in principle, but only with a single term, describing insulin-regulated glucose uptake. In our new updated liver model, we have introduced the major glucose and protein fluxes in the liver, which involve the intracellular glucose concentration, storage and breakdown of glycogen, glycolysis, and gluconeogenesis converting between glucose and pyruvate, and uptake, usage, and release of both glucose and amino acids (Fig 1D). All of these fluxes are changing both in response to a meal, and in response to more long-term changes seen for example in fasting conditions.

In the model, the main regulator of these long-term changes is glycogen, which concentration serves as a proxy for the overall energy status in the body. In other words, when glycogen concentration is high in the model, anabolic processes are upregulated and catabolic processes are downregulated, and *vice versa* when glycogen levels are low (Figure 1D, blue dashed arrow). These glycogen-dependent changes in the metabolic fluxes imply that the model can produce hypoglycaemic conditions, with plasma glucose concentration below 3.9 mM. In these hypoglycaemic conditions, the previous expression for insulin production did not work properly; therefore, that expression had to be amended.

Also on the short-term meal response level, we have improved the model, especially by adding protein dynamics (Fig 2). In the previous Herrgårdh model (9), only glucose and insulin were included, and any meal consisting of e.g. proteins, had to either ignore the protein content (Fig 2Ai), or convert it to glucose equivalents using a phenomenological conversion rate (Fig 2Aii). These are two unsatisfactory options, and we have therefore added protein states which enter the gut, from which it is transported to the intestines, where proteins are digested to amino acids, which then are transported to other organs including the liver. In the other organs, amino acids are simply consumed. In the liver, amino acids are further broken down to pyruvate and other substances which enter the tricarboxylic acid (TCA) cycle, which then leads to metabolic consumption or gluconeogenesis.

### **References**

1. C. Dalla Man, R. A. Rizza and C. Cobelli, "Meal Simulation Model of the Glucose-Insulin System," in IEEE Transactions on Biomedical Engineering, vol. 54, no. 10, pp. 1740-1749, Oct. 2007
2. Nadler SB, Hidalgo JH, Bloch T. Prediction of blood volume in normal human adults. Surgery. 1962 Feb;51(2):224-32. PMID: 21936146.
3. Taylor R, Magnusson I, Rothman DL, Cline GW, Caumo A, Cobelli C, Shulman GI. Direct assessment of liver glycogen storage by 13C nuclear magnetic resonance spectroscopy and regulation of glucose homeostasis after a mixed meal in normal subjects. J Clin Invest. 1996 Jan 1;97(1):126-32. doi: 10.1172/JCI118379. PMID: 8550823; PMCID: PMC507070.
4. Krssak M, Brehm A, Bernroider E, Anderwald C, Nowotny P, Dalla Man C, Cobelli C, Cline GW, Shulman GI, Waldhäusl W, Roden M. Alterations in postprandial hepatic glycogen metabolism in type 2 diabetes. Diabetes. 2004 Dec;53(12):3048-56. doi: 10.2337/diabetes.53.12.3048. PMID: 15561933.
5. Rothman DL, Magnusson I, Katz LD, Shulman RG, Shulman GI. Quantitation of hepatic glycogenolysis and gluconeogenesis in fasting humans with 13C NMR. Science. 1991 Oct 25;254(5031):573-6. doi: 10.1126/science.1948033. PMID: 1948033.
6. Magnusson I, Rothman DL, Katz LD, Shulman RG, Shulman GI. Increased rate of gluconeogenesis in type II diabetes mellitus. A 13C nuclear magnetic resonance study. J Clin Invest. 1992 Oct;90(4):1323-7. doi: 10.1172/JCI115997. PMID: 1401068; PMCID: PMC443176.
7. Firth RG, Bell PM, Marsh HM, Hansen I, Rizza RA. Postprandial hyperglycemia in patients with noninsulin-dependent diabetes mellitus. Role of hepatic and extrahepatic tissues. J Clin Invest. 1986 May;77(5):1525-32. doi: 10.1172/JCI112467. PMID: 3517067; PMCID: PMC424555.
8. Lerche S, Soendergaard L, Rungby J, Moeller N, Holst JJ, Schmitz OE, Brock B. No increased risk of hypoglycaemic episodes during 48 h of subcutaneous glucagon-like-peptide-1 administration in fasting healthy subjects. Clin Endocrinol (Oxf). 2009 Oct;71(4):500-6. doi: 10.1111/j.1365-2265.2008.03510.x. Epub 2008 Dec 15. PMID: 19094067.
9. Herrgårdh T, Li H, Nyman E, Cedersund G. An Updated Organ-Based Multi-Level Model for Glucose Homeostasis: Organ Distributions, Timing, and Impact of Blood Flow. Front Physiol. 2021 Jun 1;12:619254. doi: 10.3389/fphys.2021.619254. PMID: 34140893; PMCID: PMC8204084.
10. Nyman E, Brännmark C, Palmér R, Brugård J, Nyström FH, Strålfors P, Cedersund G. A hierarchical whole-body modeling approach elucidates the link between in Vitro insulin signaling and in Vivo glucose homeostasis. J Biol Chem. 2011 Jul 22;286(29):26028-41. doi: 10.1074/jbc.M110.188987. Epub 2011 May 13. PMID: 21572040; PMCID: PMC3138269.
